# One Dimensional Energy Cascades in a Fractional Quasilinear NLS

**DOI:** 10.1007/s00205-025-02159-z

**Published:** 2025-12-12

**Authors:** Alberto Maspero, Federico Murgante

**Affiliations:** 1 International School for Advanced Studies (SISSA), Via Bonomea 265, 34136 Trieste, Italy; 2 Department of Mathematics Federigo Enriques, Università degli studi di Milano, Via Saldini 50, 20133 Milan, Italy

## Abstract

We consider the problem of transfer of energy to high frequencies in a quasilinear Schrödinger equation with sublinear dispersion, on the one dimensional torus. We exhibit initial data undergoing finite but arbitrary large Sobolev norm explosion: their initial norm is arbitrary small in Sobolev spaces of high regularity, but at a later time becomes arbitrary large. We develop a novel mechanism producing instability, which is based on extracting, via paradifferential normal forms, an effective equation driving the dynamics whose leading term is a non-trivial transport operator with non-constant coefficients. We prove that such an operator is responsible for energy cascades via a positive commutator estimate inspired by Mourre’s commutator theory.

## Introduction

A fundamental question in physics and mathematical analysis is to study how energy is transferred and redistributed from macro to micro scales in deterministic systems; this is central to understanding the emergence of turbulent dynamics, especially in fluids. Formal computations of energy transfers have been performed since the 1960s, first by Hasselmann for the pure gravity water waves [43, 44], that by Longuet-Higgins and Gill for the $\beta$-plane equation [56], and more recently for the dispersive surface quasi-geostrophic equation (SQG) [68]; these, however, still lack rigorous mathematical justification.

A rigorous way to effectively capture energy transfers is to construct solutions exhibiting growth of Sobolev norms, as pointed out for example by Bourgain [18] in the context of nonlinear Hamiltonian PDEs. Whereas an active line of research—starting from the breakthrough work by Colliander-Keel-Staffilani-Takaoka-Tao [19]—has rigorously proved that growth of Sobolev norms for certain *semilinear* Schrödinger equations [36, 38–42, 45], there are no rigorous results for *quasilinear dispersive* equations, even though the most relevant dispersive models in fluid dynamics—such as those mentioned at the very beginning—are of quasilinear type.

There are several resons for that difficulties noted above. The first one, common for all dispersive equations, is that the linearized waves merely oscillate over time and consequently any growth in Sobolev norms is a purely nonlinear mechanism, making the analysis particularly challenging. A further difficulty, specific to quasilinear PDEs on compact manifolds, is that global well posedness is (usually) not known, in contrast with the (subcritical) semilinear setting. In addition, growth of Sobolev norms happens on time scales longer than those predicted by the long-time Cauchy theory (obtained via modern quasi-linear normal forms and modified energy methods), posing the problem of constructing solutions with a lifespan longer than the expected one.

This paper aims to initiate a rigorous study of energy transfers in *quasilinear dispersive PDEs* by proposing a new paradigm for constructing solutions that exhibit growth of Sobolev norms, and we believe that it could serve as a foundational framework to rigorously study energy transfers in dispersive fluid equations, such as those mentioned at the beginning. Note that the pure gravity water waves, the $\beta$-plane equation and the dispersive SQG share two common features: a nonlinear transport term and a sublinear dispersion relation. We propose a simplified model retaining exactly these features, and employ it as a theoretical test-bed to explore our new mechanism.

Specifically, we consider the fractional quasilinear NLS (nonlinear Schrödinger) equation

1.1 $$\begin{aligned} \partial _t u = - \textrm{i}|D|^\alpha u + |u|^2 u_x , \quad x \in {{\mathbb {T}}}:= {{\mathbb {R}}}/2\pi {{\mathbb {Z}}}\ , \quad \alpha \in (0,1) \ , \end{aligned}$$

with $|D|^\alpha$ the Fourier multiplier defined by $|D|^\alpha e^{\textrm{i}k x} = |k|^\alpha e^{\textrm{i}k x}$, $k \in {{\mathbb {Z}}}$. Note that, by energy methods and in view of the hyperbolic structure of the nonlinearity, equation (1.1) is locally wellposed^1^ in $H^s({{\mathbb {T}}}, {{\mathbb {C}}})$ for any $s>\frac{3}{2}$; see Remark 4.3. Here $H^s:=H^s({{\mathbb {T}}}, {{\mathbb {C}}})$, $s \in {{\mathbb {R}}}$, is the Sobolev space with norm

$$\begin{aligned} \Vert u(t) \Vert _s^2:= \sum _{k \in {{\mathbb {Z}}}} \langle k \rangle ^{2s} \, | u_k(t)|^2 \ , \quad \langle k \rangle :=\max (1, |k|) \ , \end{aligned}$$

and $u_k(t):= \frac{1}{2\pi }\int _{\mathbb {T}}u(x) e^{- \textrm{i}k x }\,\textrm{d}x$ is the *k*-th Fourier coefficient.

Equation (1.1) is also gauge invariant, so the $L^2$-norm is constant in time. Therefore, a growth in time of the $H^s$ norm, $s \gg 1$, indicates a transfer of energy to high frequencies. Our main result is the construction of a solution with Sobolev norm arbitrary small at initial time, but arbitrarily large at a later one. More precisely we prove

### Theorem 1.1

There exists $s_0> \frac{3}{2}$ such that given any $s > 3 s_0$, $0< \delta \le 1$ and $K \ge 1$, there exists a solution $u(t) \in H^s({{\mathbb {T}}}, {{\mathbb {C}}})$ of (1.1) and a time $T>0$ such that

$$\begin{aligned} \Vert u(0) \Vert _s \le \delta \quad \text{ and } \quad \Vert u(T) \Vert _s \ge K \ . \end{aligned}$$

Moreover

$$\begin{aligned}\sup \limits _{0 \le t \le T}\Vert u(t) \Vert _{s_0} \le 2 \delta \ . \end{aligned}$$

Theorem 1.1 guarantees the existence of a solution of (1.1) with smooth and arbitrary small initial datum undergoing finite but arbitrary large Sobolev norm explosion. Such a solution has a constant $L^2$-norm and stays small in the “low” $H^{s_0}$-norm. Local Cauchy theory, given by energy methods, implies that $\Vert u(t) \Vert _s \le 2 \delta$ for all times $|t| \le C\delta ^{-2}$; see Remark 4.3. We show that Sobolev norm explosion happens on the just longer timescale $T \sim \delta ^{-2} \log (\delta ^{-1})$. Of course, one of the crucial difficulties is to ensure existence of the solution over this longer timescale. We do not know the fate of such solution after time *T*, and since global existence for (1.1) is not established, we cannot exclude the possibility that, after time *T*, energy cascades trigger a finite-time singularity formation. We remark that, in similar models, such as the fractional KdV equation, solutions with large initial data can develop shocks [20, 48–51, 65, 72], resulting in the $H^1$ norm exploding while the $L^\infty$ one stays bounded. However, these shock solutions appear distinct from those described in our Theorem1.1, for which we ensure that low Sobolev norms stay small.

On the other end of things, not every initial data gives rise to turbulent solutions of (1.1): consider for example the plane waves $a e^{\textrm{i}(k x - \omega t)}$ with $\omega = |k|^\alpha - a^2 k$, which can be made of arbitrary small size. We also expect that KAM methods, like those developed in [6, 12, 27], would enable the construction of globally defined, small-amplitude, time quasi-periodic solutions, demonstrating the coexistence of stable and unstable dynamics.

As mentioned earlier, the primary novelty of this paper is the introduction of a new mechanism for generating energy cascades, tailored to quasilinear dispersive PDEs with a sublinear dispersion relation and a nonlinear transport term. In brief, such structure allows us to extract, via a novel quasilinear normal form, a transport operator with *absolutely continuous spectrum*, that drives the dynamics of (1.1), inducing dispersive effects in frequency space and resulting in the growth of Sobolev norms.

Such a mechanism is entirely distinct from the *only two* existing ones developed for semilinear Hamiltonian PDEs: the first one, pioneered by Colliander-Keel-Staffilani-Takaoka-Tao [19], exploits the dynamics of the so-called “toy model” and works for semilinear NLS on ${{\mathbb {T}}}^d$, $d \ge 2$, and some related models [19, 36, 38–42, 45]. The second one, discovered by Gérard-Grellier [32], leverages the peculiar integrable structure of the Szegő equation. We stress again that, in all these models, the nonlinearity is semilinear, in contrast to all relevant dispersive PDEs coming from fluids which are quasilinear.

Let us now describe our mechanism in more better. After a paradifferential normal form à-la Berti-Delort [9], we conjugate equation (1.1) to

1.2 $$\begin{aligned} \partial _t w = -\textrm{i}|D|^\alpha w + \textrm{Op}^{\!\scriptscriptstyle BW}\!\left( \textrm{i}\langle \underline{{\mathtt V}}\rangle (u(t);x) \xi \right) w {+ \text{ quasilinear } \text{ remainders } }, \end{aligned}$$

where $\textrm{Op}^{\!\scriptscriptstyle BW}\!\left( \cdot \right)$ is a Bony-Weyl paradifferential operator (see (2.22)) of order one, coming from the nonlinearity of (1.1), and with the transport term having *non-constant coefficient*

1.3 $$\begin{aligned} \langle \,\underline{{\mathtt V}}\, \rangle (u(t);x) := 2\, \textrm{Re }\Big (\sum _{n \in {{\mathbb {N}}}} u_{n}(t) \, \overline{u_{-n}(t)} \, e^{\textrm{i}2n x} \Big ) \ . \end{aligned}$$

This normal form is significantly different from the one of Berti-Delort [9] and of [10, 11, 13, 29, 63], where the symbol of the paradifferential operator has *constant* coefficients (at least at low homogeneity). It is also very different from the normal form of [19]: indeed the nonlinear vector field in (1.2) is *not Birkhoff-resonant*, since the main term $\textrm{Op}^{\!\scriptscriptstyle BW}\!\left( \textrm{i}\langle \underline{{\mathtt V}}\rangle (u(t);x) \xi \right) w$ has phases of oscillations given by

$$\begin{aligned} |n|^\alpha - |-n|^\alpha + |j+2n|^\alpha - |j|^\alpha \ne 0 \ , \quad \forall n \in {{\mathbb {N}}}\ , j \in {{\mathbb {Z}}}\ ; \end{aligned}$$

in principle it might be eliminated by a (formal) Birkhoff normal form procedure, but the required transformation is unbounded and not well defined in $H^s$, due to the quasi-linear nature of the problem. Actually, it will be exactly this term to drive the instability: energy cascades are due to quasi-resonant interactions rather than exact resonances; this is reminiscent, in wave turbulence, to the fact that are quasi-resonances (rather than resonances) to play a fundamental role in the rigorous derivation of the wave kinetic equation [24].

Note that the normal form (1.2) guarantees only a cubic lifespan $\sim \delta ^{-2}$ for initial data of size $\delta \ll 1$, which is too short to observe any energy transfers phenomena. Here come the first novelty of our method. We give up the control of *any* solution for times longer than $\sim \delta ^{-2}$, and restrict to particular solutions whose initial data is mostly concentrated on the two Fourier modes $\Lambda :=\{-1, 1\}$. Via an ad-hoc normal form, we decouple the dynamics of the modes in $\Lambda$ and in $\Lambda ^c$, and prove that such special solutions are long-time controlled: with this we mean that, on the enhanced timescale $\delta ^{-2}\log \delta ^{-1}$, the modes in $\Lambda$ evolve essentially as rotations, whereas the modes on $\Lambda ^c$ remain of very small size in a low $H^{s_0}$ norm. In addition, we prove that long-time controlled solutions fulfill an effective system of the form

1.4 $$\begin{aligned} \partial _t \zeta = - \textrm{i}|D|^\alpha \zeta + \textrm{i}\textrm{Op}^{\!\scriptscriptstyle BW}\!\left( {({\mathtt J}_1+\mathfrak {v}(x ))} \xi \right) \zeta {+ \text{ quasilinear } \text{ remainders } } \end{aligned}$$

Here ${\mathtt J}_1$ is a real number and $\mathfrak {v}(x)$ a real valued function, both depending nonlinearly on the initial data *u*(0) (see (5.25) and (5.26)). We develop a new robust way to prove that (1.4) has solutions undergoing growth of Sobolev norms. To do this, we extend to the nonlinear setting a positive commutator method, inspired by Mourre’s theory [64]. More precisely, we construct a paradifferential operator $\textsf{A}$, see (6.6), such that the commutator

$$\begin{aligned}\textrm{i}[\textsf{A}, \textrm{Op}^{\!\scriptscriptstyle BW}\!\left( {({\mathtt J}_1+\mathfrak {v}(x ))} \xi \right) ]\end{aligned}$$

is strictly positive on large frequencies up to a small remainder. This is possible provided that the function ${\mathtt J}_1 + \mathfrak {v}(x)$ does not have sign, a condition that we force by tuning the initial datum. This condition carries significant meaning: it ensures that the operator $\textrm{Op}^{\!\scriptscriptstyle BW}\!\left( {({\mathtt J}_1+\mathfrak {v}(x ))} \xi \right)$ has non-trivial *absolutely continuous spectrum.* This feature is the key factor driving energy transport to high frequencies: it induces a dispersive effect in the energy space that is directly analogous, in frequency variables, to the classical mechanism of spatial mass transport to infinity in Schrödinger equations on Euclidean spaces.

A further benefit of our method is that it allows us to prove that $\zeta (t)$ grows at an exponentially fast rate. This is due to the quasilinear nature of equation (1.1): for semilinear NLS, polynomial upper bounds in time are known (see e.g. [15, 66, 69, 70]), which become subpolynomial in time for linear time-dependent Schrödinger equations (see e.g. [2, 4, 5, 17, 22, 61]).

**Related literature:** Whereas for linear time dependent equations several results are known [1, 3, 16, 23, 26, 47, 54, 55, 57–60], for nonlinear systems, as we already mentioned, the results are scarce and limited to essentially two models: the semilinear Schrödinger equation (NLS) and certain integrable equations. Regarding the first, after the seminal works by Kuksin [52, 53], the breakthrough result by Colliander-Keel-Staffilani-Takaoka-Tao [19] for the NLS on ${{\mathbb {T}}}^d$, $d \ge 2$, identified the first mechanism of growth, based on the toy-model construction. Such mechanism was further exploited by Guardia-Kaloshin [41], Haus-Procesi [45], Guardia-Haus-Procesi [40], Guardia-Giuliani [36] and Giuliani [38]. All of these results construct solutions starting with norm arbitrally small and becoming arbitrarily large at a later time. We also mention Hani [42] and Guardia-Haus-Hani-Maspero-Procesi [39] that construct solutions undergoing Sobolev norm inflation and starting arbitrary close to periodic or quasi-periodic orbits. Solutions with unbounded paths have been constructed by Hani-Pausader-Tzvetkov-Visciglia [46] for the NLS on ${{\mathbb {R}}}\times {{\mathbb {T}}}^2$, combining dispersive effects and the resonant toy-model construction.

The second known mechanism ensuring growth of Sobolev norms was pioneered by Gérard-Grellier [32] for the Szegő equation, exploiting its peculiar integrable structure [31]. We also mention Biasi-Evnin [7] for a truncated Szegő systems, Gérard-Lenzmann [34] for the integrable Calogero-Moser derivative NLS, and long time instability results for the cubic half-wave equation obtained by Gérard-Grellier [33] on ${{\mathbb {T}}}$ and Gérard-Lenzmann-Popovnicu-Raphael [35] on ${{\mathbb {R}}}$ (exploiting resonant approximations with the Szegő equation). Furthermore, we mention Guardia-Giuliani [37] for chains of infinite pendula, the recent numerical result by Gallone-Marian-Ponno-Ruffo [30] for the FPUT chain and Elgindi-Shikh Khalil [25] for a completely different norm inflation mechanism in $L^\infty$.

### Scheme of the proof

We shall now describe in more detail the methods of the proof and the plan of the paper.

**Step 1: paradifferential normal form.** The first step is to transform equation (1.1) via the paradifferential normal form pioneered by Berti-Delort [9], further developed and extended in [8, 10, 11, 13, 28, 29, 63]. While previous applications of the Berti-Delort method aimed primarily at constructing a modified energy to establish *upper bounds* on the Sobolev norms of solutions, our approach leverages the method to extract an effective equation that has unstable solutions.

In Section 4, we perform two paradifferential transformations to conjugate the original equation (1.1) to the normal form system (4.23), whose cubic component has the form

1.5 $$\begin{aligned} \partial _t w= &   -\text {i}|D|^\alpha w + \text {Op}^{\!\scriptscriptstyle BW}\!\left( \text {i}\langle \underline{{\mathtt V}}\rangle (u(t);x) \xi + \text {i}a_2^{(\alpha )}(u(t);x, \xi ) \right) w \nonumber \\    &   + R_2(u(t))w + h.o.t, \end{aligned}$$

with $\langle \,\underline{{\mathtt V}}\, \rangle (u(t);x)$ in (1.3), $a_2^{(\alpha )}$ a symbol of order $\alpha$ and quadratic in *u*(*t*), and $R_2(u(t))$ a smoothing operator again quadratic in *u*. This normal form is significantly different from the one of [9] and of [10, 11, 13, 29, 63], where the symbol of the paradifferential operator has *constant* coefficients (at least at low homogeneity). On the contrary, in (1.5), $\langle \,\underline{{\mathtt V}}\, \rangle (u;x)$ has *non-constant coefficients*, and additionally it depends on time through *u*(*t*). This is the term that will give rise to the paradifferential operator in (1.4). To do this, we need to remove (or at least simplify) such time dependence. The first natural attempt, i.e. replace in $\langle \,\underline{{\mathtt V}}\, \rangle (u(t);x)$ the function *u*(*t*) with its linear evolution $e^{- \textrm{i}|D|^\alpha t}u(0)$, fails because it produces an error that we cannot bound on the long time scales needed to see growth. Therefore, we need to study the nonlinear dynamics of at least two modes $u_n(t)$, $u_{-n}(t)$. This we fix the modes in $\Lambda := \{-1, 1 \}$ and study the nonlinear dynamics of $u_1(t)$, $u_{-1}(t)$.

**Step 2: the** $\Lambda$-**normal form.** We decompose the solution as follows.

$$\begin{aligned} u(t) = u^\top (t) + u^\perp&(t)\quad \text {where}\\ u^\top (t):= u_1(t) e^{\textrm{i}x} + u_{-1}(t) e^{- \textrm{i}x} \ ,&\quad u^\perp (t) := \sum _{k \ne \pm 1} u_k(t) e^{\textrm{i}k x}. \end{aligned}$$

This decomposition separates the *tangential modes* $$u^\top (t)$$ from the *normal modes* $$u^\perp (t)$$. To decouple the dynamics of these modes, we use a weak-normal form. The paradifferential operator in equation (1.5) vanishes when restricted to $$\Lambda $$ (see (5.9)). Therefore, the dynamics of $$ u^\top (t) $$ is governed by the smoothing operator $$ R_2(u)w$$.

We decouple the dynamics of the tangential and normal modes in $R_2(u)w$ by removing from this term two types of monomials $u_{j_1}^{\sigma _1} u_{j_2}^{\sigma _2} u_{j_3}^{\sigma _3} e^{\textrm{i}k x}$:

(i) **Monomials with** $(j_1, j_2, j_3) \in \Lambda$ **and** $k \in \Lambda ^c$: $\bullet$ This ensures that the set $\Lambda$ remains invariant under the cubic part dynamics of (1.5); $\bullet$ It also requires first-order Melnikov conditions
  $$\begin{aligned} |j_1|^\alpha - |j_2|^\alpha + |j_3|^\alpha - |k|^\alpha \ne 0 \ , \quad j_1-j_2+j_3-k=0 \ , \end{aligned}$$
  that we verify whenever one and only one among $(j_1, j_2, j_3, k)$ lies in $\Lambda ^{{c}}$.
(ii) **Monomials with exactly two indexes among** $(j_1, j_2, j_3)$ **in** $\Lambda$ **and the remaining one and** *k* **in** $\Lambda ^c$: $\bullet$ This is needed so that the leading term in equation (1.5) is given by the skewadjoint paradifferential term $\textrm{Op}^{\!\scriptscriptstyle BW}\!\left( \textrm{i}\langle \underline{{\mathtt V}}\rangle (u_1 \overline{u_{-1}}; x) \xi \right) w$ (whose monomials have exactly 2 indexes inside $\Lambda$ and 2 outside); $\bullet$ It also requires second-order Melnikov conditions
  $$\begin{aligned} |j_1|^\alpha - |j_2|^\alpha + |j_3|^\alpha - |k|^\alpha \ne 0 \ , \quad j_1-j_2+j_3-k=0 \end{aligned}$$
  when two indexes among $(j_1, j_2, j_3, k)$ are in $\Lambda$ and the other two in $\Lambda ^c$, provided that $j_1 \ne j_2$ or $j_1 \ne k$.

As a result, only integrable monomials of the form $|u_{j_1}|^2 u_{j_3} e^{\textrm{i}j_3 x}$, with either $j_1, j_3 \in \Lambda$ or $j_1 \in \Lambda ,\, j_3 \in \Lambda ^c$ or viceversa are left in the smoothing operator $R_2(u)w$. Finally, in Proposition 4.11, we identify the remaining resonant integrable monomials via an a-posteriori identification argument à la Berti-Feola-Pusateri [11] (see also [10]), obtaining the explicit form (4.10).

**Step 3: The effective equation.** The variables $z^\top (t)$ and $z^\perp (t)$ solve system (5.3)–(5.4), which has roughly the form

1.6 $$\begin{aligned} {\left\{ \begin{array}{ll} \partial _t z^\top = - \textrm{i}|D|^\alpha z^\top + Y^{(\Lambda )}_3(z^\top (t)) + {{\mathcal {O}}}(\Vert z^\perp \Vert _{s_0}^3, \Vert z \Vert _{s_0}^5)\\ \partial _t z^\perp = \textrm{i}|D|^\alpha z^\perp + \textrm{Op}^{\!\scriptscriptstyle BW}\!\left( \textrm{i}\langle \underline{{\mathtt V}} \rangle (z^\top (t);x) \xi + \textrm{i}a^{(\alpha )}_2(z(t); x, \xi )\right) z^\perp \\ \quad + {{\mathcal {O}}}(\Vert z^\top \Vert _{s_0} \Vert z^\perp \Vert _{s_0} \Vert z^\perp \Vert _s), \end{array}\right. } \end{aligned}$$

where $Y^{(\Lambda )}_3(z^\top )$ is the explicit *integrable* vector field (5.5), and the symbol of the transport operator in the equation for $z^\perp$ is evaluated only on the tangential modes $z^\top (t)$.

To further understand the dynamics of system (1.6) and to extract from it the effective equation (1.4), we introduce a small parameter ${\epsilon }\ll \delta \le 1$ and we consider special solutions of system (1.6), that we call *long-time controlled* (see Definition 5.2). They are characterized by two properties:

(i) Their initial data are small in $L^2$, with most mass on the modes $z_1(0), z_{-1}(0)$:
  $$\begin{aligned} \Vert z^\top (0, \cdot ) \Vert _{L^2} \le {\epsilon }, \quad \Vert z^\perp (0, \cdot ) \Vert _{L^2} \le {\epsilon }^{3}; \end{aligned}$$
(ii) Their high $H^s$-norms have large a-priori bounds:
  $$\begin{aligned}\Vert z(t) \Vert _s \le {\epsilon }^{-\theta } \quad \text {with } 0<\theta \ll 1\ . \end{aligned}$$

Note that the large a-priori bound above is not restrictive for our problem: if it fails, it means the solution has already grown. We then prove that *any* long-time controlled solution, on the enhanced timescale $| t| \lesssim {\epsilon }^{-2} \log ({\epsilon }^{-1})$, has

$$ \bullet $$ The modes $$z_1(t)$$ and $$ z_{-1}(t)$$ evolving very close to the rotations:

$$\begin{aligned}z_{\pm 1}(t)= e^{- \textrm{i}t(1\pm |z_{\pm 1}(0)|^2)} z_{\pm 1}(0)+ {{\mathcal {O}}}( {\epsilon }^{3-\theta });\end{aligned}$$

$\bullet$ The “low” $H^{s_0}$-norm of $z^\perp (t)$ staying very small, i.e. $\Vert z^\perp (t) \Vert _{s_0} \le {\epsilon }^2$. One key idea to obtain this is to estimate $z^\perp (t)$ in $L^2$, exploiting the cancellation coming from the skewadjointness of the paradifferential operator, then deducing a bound for $\Vert z^\perp (t)\Vert _{s_0}$ by interpolation with the large a-priori bound for $\Vert z(t)\Vert _{s}$.

Finally, we approximate the evolution of $z^\top (t)$ with the rotations $e^{- \textrm{i}t(1\pm |z_{\pm 1}(0)|^2)} z_{\pm 1}(0)$ in the symbol $\langle \underline{{\mathtt V}} \rangle (z^\top (t);x)$ obtaining a negligible remainder, and, after a space translation, we arrive at an effective system of the form (1.4); see Proposition 5.4.

**Step 4: Growth of Sobolev norms.** After this analysis, we have essentially reduced the problem to construct solutions of the effective equation (1.4) undergoing growth of Sobolev norms. We construct a paradifferential operator $\textsf{A}$, of order 2*s* and supported on high-frequencies, see (6.6), fulfilling the positive commutator estimate (Lemma 6.2)

1.7 $$\begin{aligned} \textrm{i}[\textsf{A}, \textrm{Op}^{\!\scriptscriptstyle BW}\!\left( ({\mathtt J}_1 + \mathfrak {v}(x))\xi \right) ] \ge {\mathtt I}_1 \textrm{Op}^{\!\scriptscriptstyle BW}\!\left( |\xi |^{2s} \eta _{\mathtt R}^2(\xi )\right) + h.o.t.. \end{aligned}$$

Here ${\mathtt I}_1$ is a strictly positive real number depending on the initial data, see (6.10), and $\eta _{\mathtt R}$ a cut-off function on high frequencies. To obtain such positive commutator estimate, the main ingredient is to find a symbol ${\mathfrak {a}}(x, \xi )$ which is an escape-function for the dynamics of $({\mathtt J}_1 + \mathfrak {v}(x))\xi$, namely such that the Poisson bracket $\{{\mathfrak {a}}(x, \xi ), ({\mathtt J}_1 + \mathfrak {v}(x))\xi \}$ is strictly positive. This is possible provided that function ${\mathtt J}_1 + \mathfrak {v}(x)$ does not have a sign, and since

$$\begin{aligned} {\mathtt J}_1 + \mathfrak {v}(x)= \frac{|z_1(0)|^2 + |z_{-1}(0)|^2}{2} + 2\textrm{Re }\big ( z_1(0) \overline{z_{-1}(0)} e^{\textrm{i}2x } \big ) , \end{aligned}$$

it is enough to select the values of the initial modes $z_{\pm 1}(0)$ so that $\frac{|z_1(0)|^2 + |z_{-1}(0)|^2}{2} < 2 |z_1(0) | \, |{z_{-1}(0)} |$. The same condition yields the strict positivity of the number ${\mathtt I}_1$ in (1.7). An important point is that the operator $\textsf{A}$ is chosen to be supported on very large $|\xi | \ge {\mathtt R}\ge {\epsilon }^{-\frac{3+\theta }{1-\alpha }}$. This is required so that the dispersive term $-\textrm{i}|D|^\alpha$ and all the other lower order operators becomes perturbative with respect to the leading transport. To conclude, we define the functional ${{\mathcal {A}}}(t):= \langle \textsf{A}z^\perp , z^\perp \rangle$ and show that (1.7) leads to a lower bound for the dynamics of $\frac{\textrm{d}}{\textrm{d}t} {{\mathcal {A}}}(t)$, forcing ${{\mathcal {A}}}(t)$ to grow exponentially fast provided ${{\mathcal {A}}}(0)$ is not too small, a condition that can be imposed by preparing well the initial data. Cover that $\mathcal {A}(t)\lesssim \Vert z^\perp (t)\Vert _s^2$, the growth of the Sobolev norms follows.

## Functional Setting

In this section we introduce the paradifferential operators and smoothing remainders, following [9, 13]. We also introduce a new class of transformations, that we call *admissible transformations*, see Definition 2.11. They are maps $U \mapsto \textbf{F}(U)$ whose main property is to be of regularity $C^1$ with respect to the internal variable. Consequently, the nonlinear map $U \mapsto \textbf{F}(U)U$ results invertible. We shall prove that all the transformation generated along the normal form reduction of Section 4 are admissible.

**Function spaces.** Along the paper we deal with real parameters $s\ge s_0 \gg \varrho$. We use the following conventions for the set of natural numbers

$$\begin{aligned} {{\mathbb {N}}}:= \{ 1, 2, \ldots \}, \quad {{\mathbb {N}}}_0:= {{\mathbb {N}}}\cup \{0\}. \end{aligned}$$

For $s\in {{\mathbb {R}}}$ we shall denote with $H^s({{\mathbb {T}}};{{\mathbb {C}}}^2)$ the space of couples of complex valued Sobolev functions in $H^s({{\mathbb {T}}}, {{\mathbb {C}}})$ and with

$$\begin{aligned} H^s_{{\mathbb {R}}}({{\mathbb {T}}};{{\mathbb {C}}}^2):=\Big \{ U= \Big ({\begin{smallmatrix} u^+ \\ u^-\end{smallmatrix}}\Big )\in H^s({{\mathbb {T}}};{{\mathbb {C}}}^2):\ u^-= \overline{u^+}\Big \} \ . \end{aligned}$$

Given that $r>0$, we set $B_{s}(r)$ the ball of radius *r* in ${H}^s\left( \mathbb {T},\mathbb {C}^2\right)$ and $B_{s,{{\mathbb {R}}}}(r)$ the ball of radius *r* in ${H}^s_{{\mathbb {R}}}\left( \mathbb {T},\mathbb {C}^2\right)$. Given an interval $I\subset {{\mathbb {R}}}$ symmetric with respect to $t = 0$ and a Banach space *X*, we use the standard notation *C*(*I*, *X*) to denote the space of continuous functions with values in *X*. Given $r>0$ we set $B_s(I;r)$ the ball of radius *r* in $C(I,{H}^s\left( \mathbb {T},\mathbb {C}^2)\right)$ and by $B_{s,{{\mathbb {R}}}}(I;r)$ the ball of radius *r* in $C(I,{H}^s_{{{\mathbb {R}}}}\left( \mathbb {T},\mathbb {C}^2)\right)$. We denote that $L^2({{\mathbb {T}}},{{\mathbb {C}}}):= H^0({{\mathbb {T}}},{{\mathbb {C}}})$ and we define

2.1 $$\begin{aligned} \langle u, v \rangle _{L^2} := \frac{1}{2\pi } \int _{{\mathbb {T}}}u(x)\, \overline{ v(x)} \, \textrm{d}x \, . \end{aligned}$$

Given $N \in {{\mathbb {N}}}_0$, we denote by $W^{N, \infty } ({{\mathbb {T}}})$ the space of continuous functions $u: {{\mathbb {T}}}\rightarrow {{\mathbb {C}}}$, $2 \pi$-periodic, whose derivatives up to order *N* are in $L^\infty$, equipped with the norm

$$\begin{aligned} \Vert u \Vert _{W^{N,\infty }} := \sum _{\ell =0}^{N} \Vert \partial _x^\ell u \Vert _{L^\infty }. \end{aligned}$$

For $N = 0$ the norm $\Vert \cdot \Vert _{W^{N,\infty }} = \Vert \cdot \Vert _{L^{\infty }}$.

We denote by $\tau _\varsigma$, $\varsigma \in {{\mathbb {R}}}$, and by ${\mathtt g}_\theta$, $\theta \in {{\mathbb {T}}}$, the translation operator respectively the phase rotation given by

2.2 $$\begin{aligned} {[}\tau _\varsigma u](x) :=u(x + \varsigma ) \ , \qquad \big [{\mathtt g}_\theta \Big ({\begin{smallmatrix}u \\ {\overline{u}}\end{smallmatrix}}\Big )\big ](x) := \begin{pmatrix}e^{\textrm{i}\theta }u(x) \\ e^{-\textrm{i}\theta }{\overline{u}}(x)\end{pmatrix} \ . \end{aligned}$$

**Symmetries of operators and vector fields.** Given a linear operator *A*(*U*) acting on $L^2({{\mathbb {T}}};{{\mathbb {C}}})$ we associate the linear operator defined by the relation

$$\begin{aligned} \overline{A}(U)[v] := \overline{A(U)[\overline{v}]} \, , \quad \forall v: {{\mathbb {T}}}\rightarrow {{\mathbb {C}}}\, . \end{aligned}$$

An operator *A* is *real * if $A = {\overline{A}}$. We say that a matrix of operators acting on $L^2({{\mathbb {T}}};{{\mathbb {C}}}^2)$ is *real-to-real*, if it has the form

2.3 $$\begin{aligned} R(U) = \left( \begin{matrix} R_{1}(U) &  R_{2}(U) \\ \overline{R_{2}}(U) &  \overline{R_{1}}(U) \end{matrix} \right) \, , \quad \forall U \in L^2_{{\mathbb {R}}}({{\mathbb {T}}}, {{\mathbb {C}}}^2) \ . \end{aligned}$$

A real-to-real matrix of operators *R*(*U*) acts in the subspace $L^2_{{\mathbb {R}}}({{\mathbb {T}}}, {{\mathbb {C}}}^2)$. If *R*(*U*) and $R'(U)$ are real-to-real operators then also $R(U)\circ R'(U)$ is real-to-real.

A matrix *R*(*U*) as in (2.3) is translation resp. gauge invariant if

2.4 $$\begin{aligned}&\tau _\varsigma \circ R(U)= R(\tau _\varsigma U )\circ \tau _\varsigma \ , \ \forall \varsigma \in {{\mathbb {R}}}\quad \text { resp. } \nonumber \\  &{\mathtt g}_\theta \circ R(U) = R({\mathtt g}_\theta U )\circ {\mathtt g}_\theta \ , \ \forall \theta \in {{\mathbb {T}}}\ . \end{aligned}$$

Similarly, we will say that a vector field

2.5 $$\begin{aligned} X(U): = \Big ({\begin{smallmatrix}X(U)^+ \\ X(U)^-\end{smallmatrix}}\Big ) \quad \text {is real-to-real if} \quad \overline{X(U)^+}=X(U)^- \, , \quad \forall U \in L^2_{{\mathbb {R}}}({{\mathbb {T}}}, {{\mathbb {C}}}^2) \, ,\nonumber \\ \end{aligned}$$

and that it is translation resp. gauge invariant if

2.6 $$\begin{aligned} \tau _\varsigma \circ X = X \circ \tau _\varsigma \ , \quad \forall \varsigma \in {{\mathbb {R}}}\ , \qquad {\mathtt g}_\theta \circ X = X\circ {\mathtt g}_\theta , \quad \forall \theta \in {{\mathbb {T}}}\ . \end{aligned}$$

If *R*(*U*) in (2.3) is translation resp. gauge invariant, then the vector field $X(U):= R(U)U$ is translation resp. gauge invariant as well.

**Fourier expansion.** Given a $2\pi$-periodic function *u*(*x*) in $L^2 ({{\mathbb {T}}},{{\mathbb {C}}})$, we expand it in Fourier series as

2.7 $$\begin{aligned} u(x)= \sum _{j \in \mathbb {Z}} u_j \, e^{\textrm{i}j x}, \quad u_j:= \frac{1}{2\pi }\int _{\mathbb {T}}u(x) e^{- \textrm{i}j x }\,\textrm{d}x \, . \end{aligned}$$

We shall expand a function $U\in L^2({{\mathbb {T}}};{{\mathbb {C}}}^2)$ as

$$\begin{aligned} U=\Big ({\begin{smallmatrix}u^+ \\ u^-\end{smallmatrix}}\Big )= \sum _{\sigma \in \pm } \sum _{j \in {{\mathbb {Z}}}} {\mathtt q}^\sigma u_j^\sigma e^{\textrm{i}\sigma j x}, \quad u^\sigma _j:=\frac{1}{2\pi }\int _{\mathbb {T}}u^\sigma (x) e^{- \textrm{i}\sigma j x }\,\textrm{d}x, \end{aligned}$$

where $$ {\mathtt q}^+:= \Big ({\begin{smallmatrix}1 \\ 0\end{smallmatrix}}\Big ), \quad {\mathtt q}^-:= \Big ({\begin{smallmatrix}0 \\ 1\end{smallmatrix}}\Big ) $$.

For ${\jmath } = (j_1,\dots , j_p) \in {{\mathbb {Z}}}^p$, $p \ge 1$, and ${\sigma } = (\sigma _1,\dots ,\sigma _p)\in \{\pm \}^p$ we denote $|{\jmath }|:= \max (|j_1|.\dots , |j_p| )$ and

$$\begin{aligned} u_{{\jmath }}^{{\sigma }}:= u_{j_1}^{\sigma _1}\cdots u_{j_p}^{\sigma _p} \, , \qquad {\sigma } \cdot {\jmath } := \sigma _1 j_1 + \dots + \sigma _p j_p \, , \quad \mathbf {\sigma }\cdot \textbf{1} := \sigma _1 + \cdots + \sigma _p \ . \end{aligned}$$

We also denote by ${\mathcal {P}}_p$ the set of indexes

2.8 $$\begin{aligned} {\mathcal {P}}_p:= \left\{ ( {\jmath } , \mathbf {\sigma }) \in {{\mathbb {Z}}}^p \times \{\pm \}^p :\quad {\jmath } \cdot \mathbf {\sigma }= 0 \ , \ \ \mathbf {\sigma }\cdot \textbf{1} = 0 \right\} \ . \end{aligned}$$

**Fourier representation of homogeneous operators and vector fields.** In the sequel we shall encounter matrices of linear operators, gauge and translational invariant, of the form

2.9 $$\begin{aligned} M(U)= \begin{pmatrix}M^+_+(U)& M^-_+(U)\\ M^+_-(U)& M^-_-(U)\end{pmatrix} , \end{aligned}$$

depending on *U* in a homogeneous way. We shall call them *p*-homogeneous if they are polynomials in *U* of order *p*. We write them in Fourier as

$$\begin{aligned} \begin{aligned} M(U)V= \Big ({\begin{smallmatrix}(M(U)V)^+ \\ (M(U)V)^-\end{smallmatrix}}\Big ), \quad (M(U)V)^\sigma = \sum _{ \sigma k = \mathbf {\sigma }_{p} \cdot \mathbf {\jmath }_{p}+ \sigma ' j \atop \sigma = \mathbf {\sigma }_p \cdot \textbf{1} + \sigma ' } M_{\mathbf {\jmath }_{p}, j,k}^{\mathbf {\sigma }_{p}, \sigma ',\sigma } u_{\mathbf {\jmath }_{p}}^{\mathbf {\sigma }_{p}} v^{\sigma '}_{j} {e^{\textrm{i}\sigma k x}}\, , \end{aligned} \end{aligned}$$

where the coefficients $M_{\mathbf {\jmath }_{p}, j,k}^{\mathbf {\sigma }_{p}, \sigma ', \sigma } \in {{\mathbb {C}}}$ fulfill the the following symmetric property: for any permutation $\pi$ of $\{1, \ldots , p \}$, we have that

2.10 $$\begin{aligned} M_{j_{\pi (1)}, \ldots ,j_{\pi (p)}, j,k}^{ \sigma _{\pi (1)}, \ldots , \sigma _{\pi (p)},\sigma ',\sigma } = M_{j_{1}, \ldots , j_{p}, j,k}^{ \sigma _{1}, \ldots , \sigma _{p},\sigma ',\sigma } \, . \end{aligned}$$

The operator *M*(*U*) is real-to-real, according to definition (2.3), if and only if its coefficients fulfill

2.11 $$\begin{aligned} \overline{M_{\mathbf {\jmath }_{p}, j,k}^{\mathbf {\sigma }_{p}, \sigma ', \sigma }} = M_{\mathbf {\jmath }_{p}, j,k}^{-\mathbf {\sigma }_{p}, -\sigma ', -\sigma } \ . \end{aligned}$$

A $$ (p+1)$$-homogeneous vector field, which is gauge and translation invariant (see (2.6)), can be expressed in Fourier as: for any $$\sigma = \pm $$,

2.12 $$\begin{aligned} X(U)^\sigma = \sum _{k\in {{\mathbb {Z}}}} X(U)^\sigma _k \, e^{\textrm{i}\sigma k x} , \qquad X(U)^\sigma _k= \!\!\!\sum _{ k \sigma = {\sigma }_{p+1} \cdot {\jmath }_{p+1} \atop \sigma = {\sigma }_{p+1} \cdot \textbf{1} }\!\!\!\!\!\!\!\!\! X_{{\jmath }_{p+1}, k}^{ {\sigma }_{p+1}, \sigma } \, u^{{\sigma }_{p+1}}_{{\jmath }_{p+1}} \,, \end{aligned}$$

the last sum being in $(\mathbf {\jmath }_{p+1}, \mathbf {\sigma }_{p+1})$, and with coefficients $X_{{\jmath }_{p+1}, k}^{ {\sigma }_{p+1}, \sigma }\in {{\mathbb {C}}}$ satisfying the symmetry condition: for any permutation $\pi$ of $\{1, \ldots , p+1 \}$,

$$\begin{aligned} X_{\ j_{\pi (1)}, \ldots ,j_{\pi (p+1)}, k}^{ \sigma _{\pi (1)}, \ldots , \sigma _{\pi (p+1)},\sigma } = X_{\ j_{1}, \ldots , j_{p+1}, k}^{ \sigma _{1}, \ldots , \sigma _{p+1},\sigma } \, . \end{aligned}$$

The constraint of the indexes in (2.12) can also be written as $(\mathbf {\jmath }_{p+1}, k, \mathbf {\sigma }_{p+1}, - \sigma ) \in {{\mathcal {P}}}_{p+2}$ (recall (2.8)), and we shall often use this notation.

If *X*(*U*) is real-to-real (see (2.5)), then

$$\begin{aligned} \overline{X(U)^+_k}=X(U)^-_k\quad \text{ i.e. } \quad \overline{X_{{\jmath }_{p+1}, k}^{ { \sigma }_{p+1}, +}}=X_{{\jmath }_{p+1}, k}^{- {\sigma }_{p+1}, -} \, . \end{aligned}$$

### Paradifferential calculus

In this section we introduce paradifferential and smoothing operators, following [9, 13].

**Symbols.** We define the class of symbols which we will use along the paper. They correspond to the autonomous symbols of Definition 3.3 in [9], where the dependence on time enters only through the function $U=U(t)$. In view of this, we do not need to keep track on the regularity indexes in time and we fix $K = K' =0$ with respect to Definition 3.3 of [9].

#### Definition 2.1

(Symbols) Let $$ m \in {{\mathbb {R}}}$$, $$N \in {{\mathbb {N}}}_0$$, $$p \in {{\mathbb {N}}}$$, $$s_0, r>0$$.

1. **Hölder symbols.** We denote by $\Gamma ^m_{W^{N,\infty }}$ the space of functions $a: {{\mathbb {T}}}\times {{\mathbb {R}}}\rightarrow {{\mathbb {C}}}$, $a(x, \xi )$, which are $C^\infty$ with respect to $\xi$ and such that, for any $\beta \in {{\mathbb {N}}}_0$, there exists a constant $C_\beta >0$ such that
  $$\begin{aligned} \big \Vert \partial _\xi ^\beta \, a(\cdot , \xi ) \big \Vert _{W^{N,\infty }} \le C_\beta \, \langle \xi \rangle ^{m - |\beta |} , \quad \forall \xi \in {{\mathbb {R}}}\, . \end{aligned}$$
  We endow $\Gamma ^m_{W^{N,\infty }}$ with the family of norms defined, for any $n \in {{\mathbb {N}}}_0$, by
  2.13 $$\begin{aligned} \left| a \right| _{m, {W^{N,\infty }}, n}:= \max _{\beta \in \{0, \dots , n\} }\, \sup _{\xi \in {{\mathbb {R}}}} \, \big \Vert \langle \xi \rangle ^{-m+|\beta |} \, \partial _\xi ^{\beta } a(\cdot , \xi ) \big \Vert _{W^{N,\infty }} \ . \end{aligned}$$
2. *p*-**Homogeneous symbols.** We denote by $\widetilde{\Gamma }^m_p$ the space of *p*-linear symmetric maps from $\left( C^{\infty }\left( \mathbb {T};\mathbb {{{\mathbb {C}}}}^2\right) \right) ^p$ to $C^\infty (\mathbb {T}\times {{\mathbb {R}}}; {{\mathbb {C}}})$ , $(x, \xi ) \mapsto a_p(U_1, \dots , U_p;x,\xi )$ defined by
  2.14 $$\begin{aligned} a_p(U_1, \dots , U_p; x, \xi ):= \sum _{\begin{array}{c} {\jmath }\in {{\mathbb {Z}}}^p\\ {\sigma }\in \{ \pm \}^p \end{array}} a_{\mathbf {\jmath }}^{\mathbf {\sigma }}(\xi ) (u_1)_{j_1}^{\sigma _1} \cdots (u_p)_{j_p}^{\sigma _p}\, e^{\textrm{i}({\sigma }\cdot {\jmath }) x}, \end{aligned}$$
  where $a_{{\jmath }}^{{\sigma }}(\xi ):= a_{j_1, \dots , j_p}^{\sigma _1, \dots , \sigma _p}(\xi )$ are complex valued Fourier multipliers, satisfying
  $$\begin{aligned} a_{j_1, \dots , j_p}^{\sigma _1, \dots , \sigma _p}(\xi ) = a_{j_{\pi (1)}, \dots , j_{\pi (p)}}^{\sigma _{\pi (1)}, \dots , \sigma _{\pi (p)}} (\xi )\quad \text { for any } \pi \text { permutation of } \{1, \dots , p\}\,, \end{aligned}$$
  and for some $\mu \ge 0$,
  2.15 $$\begin{aligned} | \partial _\xi ^\beta a_{{\jmath }}^{{\sigma }}(\xi ) | \le C_\beta \langle {\jmath }\rangle ^{\mu } \langle \xi \rangle ^{m-\beta }, \quad \forall {\jmath }\in {{\mathbb {Z}}}^p,\, {\sigma }\in \{\pm \}^p, \, \beta \in {{\mathbb {N}}}_0. \end{aligned}$$
  We shall denote by
  $$\begin{aligned} a_p(U;x, \xi ):= a_p(U, \cdots , U; x, \xi ) \end{aligned}$$
  the polynomial symbol associated to the multilinear symmetric symbol.
  We denote by $\widetilde{\Gamma }^m_0$ the space of constant coefficients symbols $\xi \mapsto a(\xi )$ which satisfy (2.15) with $\mu = 0$.
3. **Non-homogeneous symbols. ** We denote by $\Gamma _{\ge p}^m[r]$ the space of functions $(U;x,\xi )\mapsto a(U;x,\xi )$, defined for $U \in B_{s_0}(r)$ for some $s_0$ large enough, with complex values, such that for any ${\mathtt s}\ge s_0$, there are $C>0$, $r':=r'({\mathtt s})\in (0,r)$ and for any $U \in B_{s_0}\left( r' \right) \cap H^{{\mathtt s}}\left( \mathbb {T};\mathbb {C}^2\right)$, any $\beta \in {{\mathbb {N}}}_0$ and $N \le {\mathtt s}- s_0$, one has the estimate
  2.16 $$\begin{aligned} \left\| \partial _\xi ^\beta a\left( { U;\cdot ,\xi }\right) \right\| _{W^{N,\infty }} \le C \langle \xi \rangle ^{m-\beta } \Vert U\Vert _{{s_0}}^{p-1}\Vert U\Vert _{{\mathtt s}} \, . \end{aligned}$$
  In addition we require also the translation invariance property
  2.17 $$\begin{aligned} a\left( \tau _{\varsigma } U; x,\xi \right) = a\left( U; x+\varsigma , \xi \right) ,\quad \forall \varsigma \in {{\mathbb {R}}}\, , \end{aligned}$$
  where $\tau _\varsigma$ is the translation operator in (2.2).
4. **Symbols.** We denote by $\Sigma \Gamma ^m_0[r]$ the class of symbols of the form
  2.18 $$\begin{aligned} a(U; x, \xi ) = a_0(\xi ) + a_2(U; x, \xi ) + a_{\ge 4}(U;x, \xi ) \end{aligned}$$
  where $a_0(\xi )\in {\widetilde{\Gamma }}^m_0$ is a Fourier multiplier, $a_2(U) \in {\widetilde{\Gamma }}_2^m$ and $a_{\ge 4}(U) \in \Gamma ^m_{\ge 4}[r]$. We denote by $\Sigma \Gamma ^m_2[r]$ the class of symbols of the form (2.18) with $a_0(\xi ) = 0$. Finally sometimes we shall write $\Sigma \Gamma ^m_4[r]\equiv \Gamma _{\ge 4}^m[r]$.

We say that a symbol $a(U;x,\xi )$ is *real* if it is real valued for any $U \in B_{s_0,{{\mathbb {R}}}}(I;r)$.

We also denote by ${\widetilde{{{\mathcal {F}}}}}_p$ (respectively ${{\mathcal {F}}}_{\ge p}[r]$) the subspace of ${\widetilde{\Gamma }}^0_p$ (respectively $\Gamma ^0_{\ge p}[r]$) made of those symbols which are independent of $\xi$, and by ${\widetilde{{{\mathcal {F}}}}}^{{\mathbb {R}}}_p$ (respectively ${{\mathcal {F}}}^{{\mathbb {R}}}_{\ge p}[r]$) to denote functions in ${\widetilde{{{\mathcal {F}}}}}_p$ (respectively ${{\mathcal {F}}}^{{\mathbb {R}}}_{\ge p}[r]$) which are real valued.

#### Remark 2.2

Sometimes we shall write a symbol $a_p(U; x, \xi )$ only in polynomial form

2.19 $$\begin{aligned} a_p(U;x,\xi ):=\sum _{\begin{array}{c} {\jmath }\in {{\mathbb {Z}}}^p\\ {\sigma }\in \{ \pm \}^p \end{array}} {\widetilde{a}}_{{\jmath }}^{{\sigma }}(\xi ) \, u_{{\jmath }}^{{\sigma }} \, e^{\textrm{i}({\sigma }\cdot {\jmath }) x}, \end{aligned}$$

with some Fourier multiplier coefficients ${\widetilde{a}}_{{\jmath }}^{{\sigma }}(\xi )$ not necessarily symmetric, but fulfilling the estimates (2.15). One obtains the symmetric coefficients $a_{j_1, \dots , j_p}^{\sigma _1, \dots , \sigma _p}$ in the expression (2.14) by symmetrizing, i.e., denoting by ${{\mathcal {S}}}_p$ the symmetric group of permutations of $\{1, \ldots , p\}$,

$$\begin{aligned} a_{j_1, \dots , j_p}^{\sigma _1, \dots , \sigma _p} = \frac{1}{p!}\sum _{\pi \in {{\mathcal {S}}}_p} {\widetilde{a}}_{j_{\pi (1)}, \dots , j_{\pi (p)}}^{\sigma _{\pi (1)}, \dots , \sigma _{\pi (p)}} \ . \end{aligned}$$

We shall use the notation (2.19) for example in formulas (4.4) and for the resonant transport term in (4.8); the reason is that the transport term (4.8) is perhaps the most important object, being the term responsible for the growth, and we prefer to express it in the simplest possible form.

$\bullet$ If *a* is a symbol in $\Gamma ^m_{W^{N,\infty }}$ then $\partial _x a \in \Gamma ^{m}_{W^{N-1,\infty }}$ and $\partial _\xi a \in \Gamma ^{m-1}_{W^{N,\infty }}$. If *b* is a symbol in $\Gamma ^{m'}_{W^{N,\infty }}$ then $a b \in \Gamma ^{m+m'}_{W^{N,\infty }}$. If $a \in \Gamma ^{m}_{\ge p}[r]$ and $b \in \Gamma ^{m'}_{\ge q}[r]$, then $ab \in \Gamma ^{m+m'}_{\ge p+q}[r]$ .

$\bullet$ *p*-homogeneous symbols in ${\widetilde{\Gamma }}^m_p$ and non-homogeneous symbols in $\Gamma ^m_{\ge p}[r]$ are actually functions with values in $\Gamma ^m_{W^{N,\infty }}$ for some $N \in {{\mathbb {N}}}$, whose seminorms (2.13) are bounded by

$$\begin{aligned} |a_p|_{m, W^{N,\infty }, n}\le &   C_n \, \Vert U\Vert _{{1}}^{p-1}\Vert U\Vert _{{N+\mu +1}} \ , \quad |a|_{m, W^{N,\infty }, n} \le C_n \, \Vert U \Vert _{{s_0}}^{p-1} \Vert U \Vert _{{\mathtt s}} \ , \\    &   \quad N \le {\mathtt s}- s_0 \ . \end{aligned}$$

$\bullet$ A *p*-homogeneous symbol $a_p(U,x,\xi )$ is a non-homogeneous symbol, since (2.14)–(2.15) imply

2.20 $$\begin{aligned} \left\| \partial _\xi ^\beta a_p\left( { U;\cdot ,\xi }\right) \right\| _{W^{N,\infty }} \le C \langle \xi \rangle ^{m-\beta } \Vert U\Vert _{{1}}^{p-1}\Vert U\Vert _{{N+\mu +1}} \, \ , \end{aligned}$$

and (2.14) implies the translation invariance property (2.17).

**Paradifferential quantization.** Given $p\in {{\mathbb {N}}}_0$ we consider functions $\chi _{p}\in C^{\infty }({{\mathbb {R}}}^{p}\times {{\mathbb {R}}};{{\mathbb {R}}})$ and $\chi \in C^{\infty }({{\mathbb {R}}}\times {{\mathbb {R}}};{{\mathbb {R}}})$, even with respect to each of their arguments, satisfying, for $0<\delta _0\le \tfrac{1}{10}$,

$$\begin{aligned}&{\textrm{supp}}\, \chi _{p} \subset \{(\xi ',\xi )\in {{\mathbb {R}}}^{p}\times {{\mathbb {R}}}; |\xi '|\le \delta _0 \langle \xi \rangle \} \, ,\qquad \chi _p (\xi ',\xi )\equiv 1\,\,\, \textrm{for } \,\,\, |\xi '|\le \delta _0 \langle \xi \rangle / 2 \, , \\&\textrm{supp}\, \chi \subset \{(\xi ',\xi )\in {{\mathbb {R}}}\times {{\mathbb {R}}}; |\xi '|\le \delta _0 \langle \xi \rangle \} \, ,\qquad \quad \chi (\xi ',\xi ) \equiv 1\,\,\, \textrm{for } \,\,\, |\xi '|\le \delta _0 \langle \xi \rangle / 2 \, . \end{aligned}$$

For $p=0$ we set $\chi _0\equiv 1$. We assume, moreover, that

$$\begin{aligned} |\partial _{\xi }^{\ell }\partial _{\xi '}^{\beta }\chi _p(\xi ',\xi )|\le &   C_{\ell ,\beta }\langle \xi \rangle ^{-\ell -|\beta |} \, , \ \forall \ell \in {{\mathbb {N}}}_0, \,\beta \in {{\mathbb {N}}}_0^{p} \, , \ \ \\ |\partial _{\xi }^{\ell }\partial _{\xi '}^{\beta }\chi (\xi ',\xi )|\le &   C_{\ell ,\beta }\langle \xi \rangle ^{-\ell -\beta }, \ \forall \ell , \,\beta \in {{\mathbb {N}}}_0 \, . \end{aligned}$$

If $a (x, \xi )$ is a smooth symbol we define its Weyl quantization as the operator acting on a $2 \pi$-periodic function *u*(*x*) (written as in (2.7)) as

$$\begin{aligned} \textrm{Op}^{W}(a)u= \sum _{k\in {{\mathbb {Z}}}} \Big (\sum _{j\in {{\mathbb {Z}}}}\hat{a}\big (k-j, \tfrac{k+j}{2}\big ) u_j \Big ){e^{\textrm{i}k x}}, \end{aligned}$$

where $\hat{a}(k,\xi )$ is the $k^{th}-$Fourier coefficient of the $2\pi -$periodic function $x\mapsto a(x,\xi )$.

#### Definition 2.3

**(Bony-Weyl quantization)** If $a(U;x,\xi )$ is a symbol in $\widetilde{\Gamma }^{m}_{p}$, respectively in $\Gamma ^m_{W^{N,\infty }}$ or $\Gamma ^{m}_{\ge p}[r]$, we set

2.21 $$\begin{aligned} a_{\chi _{p}}(U;x,\xi ):= &   \!\!\!\!\! \sum _{\begin{array}{c} {\jmath }\in {{\mathbb {Z}}}^p\\ {\sigma }\in \{ \pm \}^p \end{array}}\chi _p ({\jmath },\xi ) a_{{\jmath }}^{{\sigma }}(\xi ) u_{{\jmath }}^{{\sigma }} e^{\textrm{i}({\sigma }\cdot {\jmath }) x},\nonumber \\ a_{\chi }(U;x,\xi ):= &   \sum _{j\in {{\mathbb {Z}}}} \chi (j,\xi )\hat{a}(U;j,\xi )e^{\textrm{i}j x} \, \end{aligned}$$

where in the last equality ${\hat{a}}(U; j,\xi )$ stands for $j^{th}$ Fourier coefficient of $a(U;x, \xi )$ with respect to the *x* variable, and we define the *Bony-Weyl* quantization of $a(U; \cdot )$ as

2.22 $$\begin{aligned}&{\textrm{Op}^{{\scriptscriptstyle {\mathrm BW}}}}(a({U};\cdot ))v= \textrm{Op}^{W} (a_{\chi _{p}}({U};\cdot ))v= \!\!\!\!\!\!\!\!\!\sum _{\begin{array}{c} ({\jmath },j,k)\in {{\mathbb {Z}}}^{p+2}\\ {\sigma }\in \{ \pm \}^p\\ {\sigma }\cdot {\jmath }+j=k \end{array}}\chi _p \left( {\jmath },\frac{j+k}{2}\right) a_{{\jmath }}^{{\sigma }}\left( \frac{j+k}{2}\right) u_{{\jmath }}^{{\sigma }} v_j {e^{\textrm{i}k x}} \, , \end{aligned}$$

2.23 $$\begin{aligned}&{\textrm{Op}^{{\scriptscriptstyle {\mathrm BW}}}}(a(U;\cdot ))v= \textrm{Op}^{W} (a_{\chi }(U;\cdot ))v= \!\!\!\!\!\! \sum _{(j,k)\in {{\mathbb {Z}}}^2} \!\!\! \chi \left( k-j,\frac{j+k}{2} \right) \hat{a}\left( U; k-j, \frac{k+j}{2}\right) v_j {e^{\textrm{i}k x}} \, . \end{aligned}$$

Note that if $\chi \Big ( k-j, \tfrac{ k + j }{2}\Big ) \ne 0$ then $|k-j| \le \delta _0 \langle \frac{j + k}{2} \rangle$ and therefore, for $\delta _0 \in (0,1)$,

$$\begin{aligned} \frac{1-\delta _0}{1+\delta _0} |k| \le |j| \le \frac{1+\delta _0}{1-\delta _0}|k| \, , \quad \forall j, k \in {{\mathbb {Z}}}\, . \end{aligned}$$

This relation shows that the action of a paradifferential operator does not spread much the Fourier support of functions.

$\bullet$ If *a* is a homogeneous symbol, the two definitions of quantization in (2.22) and (2.23) differ by a smoothing operator according to Definition 2.6 below.

$\bullet$ Definition 2.3 is independent of the cut-off functions $\chi _{p}$, $\chi$, up to smoothing operators that we define below (see Definition 2.6), see the remark at page 50 of [9].

$\bullet$ Given a paradifferential operator $A = \textrm{Op}^{\!\scriptscriptstyle BW}\!\left( a(x,\xi )\right)$, we have that

$$\begin{aligned} {\overline{A}} = \textrm{Op}^{\!\scriptscriptstyle BW}\!\left( \overline{a(x, - \xi )}\right) \, , \quad A^\top = \textrm{Op}^{\!\scriptscriptstyle BW}\!\left( a(x, - \xi )\right) \, , \quad A^*= \textrm{Op}^{\!\scriptscriptstyle BW}\!\left( \overline{a(x, \xi )}\right) \, , \end{aligned}$$

where $A^\top$ and $A^*$ denote respectively the transposed and adjoint operator with respect to the complex, respectively real, scalar product of $L^2({{\mathbb {T}}}, {{\mathbb {C}}})$ in (2.1). It results $A^* = {\overline{A}}^\top$.

$\bullet$ A paradifferential operator $A= \textrm{Op}^{\!\scriptscriptstyle BW}\!\left( a(x,\xi )\right)$ is *real* (i.e. $A = {\overline{A}}$) if

2.24 $$\begin{aligned} \overline{a(x,\xi )}= a^\vee (x,\xi ) \quad \text { where} \quad a^{\vee }(x,\xi ) := a(x,-\xi ) \, . \end{aligned}$$

$\bullet$ A matrix of paradifferential operators $\textrm{Op}^{\!\scriptscriptstyle BW}\!\left( A( x,\xi )\right)$ is real-to-real, i.e. (2.3) holds, if and only if the matrix of symbols $A(x,\xi )$ has the form

2.25 $$\begin{aligned} {\small A(x,\xi ) = \left( \begin{matrix} {a}(x,\xi ) &  {b}(x,\xi )\\ {\overline{b^\vee (x,\xi )}} &  {\overline{a^\vee (x,\xi )}} \end{matrix} \right) =\left( \begin{matrix} {a}(x,\xi ) &  0\\ 0 &  {\overline{a^\vee (x,\xi )}} \end{matrix} \right) +\left( \begin{matrix} 0&  {b}(x,\xi )\\ {\overline{b^\vee ( x,\xi )}} &  0 \end{matrix} \right) \, \, . \qquad } \end{aligned}$$

$\bullet$ A real-to-real matrix of *U*-dependent paradifferential operators $\textrm{Op}^{\!\scriptscriptstyle BW}\!\left( A(U; x,\xi )\right)$ is gauge invariant, i.e. (2.4) holds, if and only if the symbols in (2.25) fulfill, with ${\mathtt g}_\theta$ in (2.2),

2.26 $$\begin{aligned} a(U; x, \xi ) = a({\mathtt g}_\theta U; x, \xi ) \ , \quad e^{\textrm{i}2 \theta } \, b(U; x, \xi ) = b({\mathtt g}_\theta U; x, \xi ) \ , \quad \forall \theta \in {{\mathbb {T}}}\ ,\qquad \end{aligned}$$

If, in addition, $a, b\in {\widetilde{\Gamma }}^m_p$, then $\textrm{Op}^{\!\scriptscriptstyle BW}\!\left( a\right)$ in (2.22) have indexes restricted to $\mathbf {\sigma }\cdot 1=0$, whereas $\textrm{Op}^{\!\scriptscriptstyle BW}\!\left( b\right)$ to $\mathbf {\sigma }\cdot 1 = 2$.

We will use also the notations

2.27 $$\begin{aligned} \begin{aligned}&\text {Op}^{\scriptscriptstyle BW}_{\texttt {vec}}\!\left( a(x,\xi )\right) := \text {Op}^{\!\scriptscriptstyle BW}\!\left( \begin{bmatrix} a(x,\xi )&  0\\ 0&  \overline{ a^{\vee }(x,\xi )}\end{bmatrix}\right) \, , \\  &\text {Op}^{\scriptscriptstyle BW}_{\texttt {out}}\!\left( b(x,\xi )\right) := \text {Op}^{\!\scriptscriptstyle BW}\!\left( \begin{bmatrix} 0&  b(x,\xi )\\ \overline{ b^{\vee }(x,\xi )}&  0\end{bmatrix}\right) \end{aligned} \end{aligned}$$

Through of this paper we shall use the following results concerning the action of a paradifferential operator in Sobolev spaces. (we refer to [13, Theorem A.7] for the proof of (*i*) and to [9, Proposition 3.8] for the proof of (*ii*), (*iii*)):

#### Theorem 2.4

(Continuity of Bony-Weyl operators) Let $m\in {{\mathbb {R}}}$, $p\in {{\mathbb {N}}}$, $r>0$. Then

(*i*) Let $a \in \Gamma ^m_{L^\infty }$. Then $\textrm{Op}^{\!\scriptscriptstyle BW}\!\left( a\right)$ extends to a bounded operator $H^{s} \rightarrow H^{s-m}$ for any $s \in {{\mathbb {R}}}$ satisfying the estimate, for any $u \in H^s$,

2.28 $$\begin{aligned}&\Vert \textrm{Op}^{\!\scriptscriptstyle BW}\!\left( a\right) u \Vert _{{s-m}} \lesssim \, \left| a \right| _{m, L^\infty , 4} \, \Vert u \Vert _{{s}} \ . \end{aligned}$$

(*ii*) Let $a\in \widetilde{\Gamma }_{p}^{m}$. There is $s_0 > 0$ such that for any $s \in {{\mathbb {R}}}$, there is a constant $C>0$, depending only on *s* and on (2.15) with $\ell =\beta =0$, such that for any $U_1,\ldots ,U_{p} \in H^{s_0}({{\mathbb {T}}}, {{\mathbb {C}}}^2)$ and $v \in H^s({{\mathbb {T}}}, {{\mathbb {C}}})$, one has

2.29 $$\begin{aligned} \Vert \textrm{Op}^{\!\scriptscriptstyle BW}\!\left( a(U_1,\dots , U_p;\cdot )\right) v\Vert _{{s-m}}\le C\prod _{j=1}^{p}\Vert U_{j}\Vert _{{s_0}}\Vert v\Vert _{{s}} \, , \end{aligned}$$

for $p\ge 1$, while for $p=0$ the (2.29) holds by replacing the right hand side with $C\Vert v\Vert _{{s}}$.

(*iii*) Let $a\in \Gamma ^{m}_{\ge p}[r]$. There is $s_0 > 0$ such that for any $s \in {{\mathbb {R}}}$ there is a constant $C>0$ such that, for any $U \in B_{s_0}(r)$, one has

2.30 $$\begin{aligned} \Vert \textrm{Op}^{\!\scriptscriptstyle BW}\!\left( a(U;\cdot )\right) \Vert _{\mathcal {L}({H}^{s},{H}^{s-m})}\le C\Vert U\Vert _{{s_0}}^{p} \, . \end{aligned}$$

**Classes of** *m*-**operators and smoothing operators.** We introduce *m*-operators and smoothing operators. This is a small adaptation of [9, 13] where we consider only autonomous maps, where again the time dependence is only through *U*(*t*). In particular we put $K,K' = 0$ with respect to the notation in [9, 13]. Given integers $(n_1,\ldots ,n_{p+1})\in {{\mathbb {N}}}^{p+1}$, we denote by $\max _{2}(n_1,\ldots , n_{p+1})$ the second largest among $n_1,\ldots , n_{p+1}$.

#### Definition 2.5

(Classes of *m*-operators) Let $m \in {{\mathbb {R}}}$, $p \in {{\mathbb {N}}}_0$ and $r >0$.

1. *p*-**homogeneous** *m*-**operators**. We denote by ${\widetilde{{{\mathcal {M}}}}}^{m}_p$ the class of $(p+1)$-linear operators from $(C^{\infty }({{\mathbb {T}}};{{\mathbb {C}}}^{2}))^{p}\times C^{\infty }({{\mathbb {T}}};{{\mathbb {C}}})$ to $C^{\infty }({{\mathbb {T}}};{{\mathbb {C}}})$ of the form $(U_{1},\ldots ,U_{p}, v)\rightarrow M_p(U_1,\ldots , U_p)v$, symmetric in $(U_{1},\ldots ,U_{p})$, with Fourier expansion
  2.31 $$\begin{aligned} M_p(U)v:= M_p(U, \ldots , U)v= \!\!\!\!\!\!\!\! \sum _{\begin{array}{c} \mathbf {\sigma }_{p}\in \{\pm \}^{p} \\ k -j = \mathbf {\sigma }_{p} \cdot \mathbf {\jmath }_{p} \end{array} } \!\!\!\!\!\! M_{\mathbf {\jmath }_{p}, j,k}^{\mathbf {\sigma }_{p}} \, u_{\mathbf {\jmath }_{p}}^{\mathbf {\sigma }_{p}}\, v_{j} \, {e^{\textrm{i}k x} } \end{aligned}$$
  that satisfy the following. There are $\mu \ge 0$, $C>0$ such that for any $(\mathbf {\jmath }_{p},j,k) \in {{\mathbb {Z}}}^{p+2}$, $\mathbf {\sigma }_p \in \{ \pm \}^{p}$, one has
  2.32 $$\begin{aligned} |M_{\mathbf {\jmath }_p, j, k}^{\mathbf {\sigma }_p} | \le C \textrm{max}_2\{ \langle j_1\rangle ,\dots , \langle j_p\rangle ,\langle j\rangle \}^{\mu }\, \max \{ \langle j_1\rangle ,\dots , \langle j_p\rangle ,\langle j\rangle \}^{m}.\qquad \end{aligned}$$
2. **Non-homogeneous** *m***-operators.** We denote by ${{\mathcal {M}}}^{m}_{\ge p}[r]$ the class of operators $(U, v)\rightarrow M(U) v$ defined on $B_{s_0}(r)\times {H}^{s_0}({{\mathbb {T}}},{{\mathbb {C}}})$ for some $s_0 >0$, which are linear in the variable *v* and such that the following holds true. For any $s\ge s_0$ there are $C>0$ and $r'=r'(s)\in ]0,r[$ such that for any $U\in B_{s_0}(r')\cap {H}^{s}({{\mathbb {T}}},{{\mathbb {C}}}^2)$, any $v \in {H}^{s}({{\mathbb {T}}},{{\mathbb {C}}})$, we have that
  2.33 $$\begin{aligned}  &   \Vert { M(U)v }\Vert _{{s - m}} \le C \left( \Vert {v}\Vert _{s}\Vert {U}\Vert _{s_0}^{p} +\Vert {v}\Vert _{{s_0}}\Vert U\Vert _{{s_0}}^{p-1}\Vert {U}\Vert _{{s}} \right) \quad \text {if} \quad p \ge 1\, ,\nonumber \\  &   \Vert { M(U)v }\Vert _{{s - m}} \le C \left( \Vert {v}\Vert _{s} +\Vert {v}\Vert _{{s_0}} \Vert {U}\Vert _{{s}}\Vert U\Vert _{s_0} \right) \quad \text {if} \quad p =0\,. \nonumber \\ \end{aligned}$$
  In addition, we require the translation invariance property
  2.34 $$\begin{aligned} M( \tau _\varsigma U) [\tau _\varsigma v] = \tau _\varsigma \big ( M(U)v \big ) \, , \quad \forall \varsigma \in {{\mathbb {R}}}\, , \end{aligned}$$
  where $\tau _\varsigma$ is the translation operator in (2.2).
3. *m*-**Operators.** We denote by $\Sigma {{\mathcal {M}}}^m_0[r]$ the space of operators $(U,v) \rightarrow M(U)v$ of the form
  2.35 $$\begin{aligned} M(U) = M_0 + M_2(U) + M_{\ge 4}(U) , \end{aligned}$$
  where $M_p(U)$ in ${\widetilde{{{\mathcal {M}}}}}_p^m$, $p\in \{0,2\}$, and $M_{\ge 4}(U)$ in ${{\mathcal {M}}}^m_{\ge 4}[r]$.
  We denote by $\Sigma {{\mathcal {M}}}^m_2[r]$ the operators of the form (2.35) with $M_0 = 0$. Finally sometimes we shall write $\Sigma {{\mathcal {M}}}^m_4[r]\equiv {{\mathcal {M}}}^m_{\ge 4}[r]$.

$\bullet$ A *p*-homogeneous *m*-operator $M_p$ is a non-homogeneous *m*-operator. Indeed, (2.32) implies the quantitative estimate: for $s_0 \ge \mu +1>0$, for any $s \ge s_0$, any $U \in H^s({{\mathbb {T}}};{{\mathbb {C}}}^2)$, any $v \in H^s({{\mathbb {T}}};{{\mathbb {C}}})$,

2.36 $$\begin{aligned} \Vert M_p(U)v \Vert _{{s-m}}\lesssim _s \Vert U \Vert _{{s_0}}^{p} \Vert V \Vert _{ s}+ \Vert U \Vert _{{s_0}}^{p-1}\Vert U \Vert _{s} \Vert V \Vert _{{s_0}}, \end{aligned}$$

which is (2.33) (see Lemma 2.8 and 2.9 in [13] for a proof). Moreover (2.34) follows from the Fourier restriction $k -j = \mathbf {\sigma }_{p} \cdot \mathbf {\jmath }_{p}$ in (2.31).

$\bullet$ **(Paradifferential operators as** *m***-operators)** If $a(U;x,\xi )$ is a symbol in $\Sigma \Gamma ^{m}_{0}[r]$ then the paradifferential operator ${\textrm{Op}^{{\scriptscriptstyle {\mathrm BW}}}}(a(U;x,\xi ))$ is an *m*-operator $\Sigma \mathcal {M}_{0}^m[r]$. This is a consequence of Theorem 2.4–(*ii*) &(*iii*).

$\bullet$ We will meet vector fields of the form $X(U) = M(U)U$ where *M*(*U*) is a matrix of *p*-homogeneous *m*-operators as in (2.9). In this case the relation between the Fourier coefficients of the vector field in (2.12) and those of the *m*-operator in (2.31) is given by

2.37 $$\begin{aligned} X_{\ j_1, \ldots , j_{p}, j_{p+1}, k}^{\sigma _1, \ldots , \sigma _{p}, \sigma _{p+1}, \sigma } = \frac{1}{p+1} \left( M_{\ j_1, \ldots , j_{p}, j_{p+1}, k}^{\sigma _1, \ldots , \sigma _{p}, \sigma _{p+1}, \sigma } + M_{\ j_{p+1}, \ldots , j_{p}, j_1, k}^{\sigma _{p+1}, \ldots , \sigma _{p}, \sigma _1, \sigma } + \cdots + M_{\ j_1, \ldots , j_{p+1}, j_{p}, k}^{\sigma _1, \ldots , \sigma _{p+1}, \sigma _{p}, \sigma } \right) ,\nonumber \\ \end{aligned}$$

namely, they are obtained symmetrizing with respect to the second last index $(j,\sigma ')$ the coefficients $M_{\ \mathbf {\jmath }_{p}, j, k}^{\mathbf {\sigma }_{p}, \sigma ',\sigma }$ of *M*(*U*).

If $m \le 0$ the *m*-operators are referred to as smoothing operators.

#### Definition 2.6

*(Smoothing operators)* Let $\varrho \ge 0$, $p\in {{\mathbb {N}}}_0$ and $q \in \{0,2\}$. We define the $\varrho$-smoothing operators

$$\begin{aligned} \begin{aligned}&\widetilde{\mathcal {R}}^{-\varrho }_{p}:= \widetilde{\mathcal {M}}^{-\varrho }_{p} \, , \quad \mathcal {R}^{-\varrho }_{\ge p}[r]:= \mathcal {M}^{-\varrho }_{ \ge p}[r] \ , \quad \Sigma \mathcal {R}^{-\varrho }_q[r]:= \Sigma \mathcal {M}^{-\varrho }_q[r] \ . \end{aligned} \end{aligned}$$

$\bullet$ In view of (2.32) a homogeneous *m*-operator in $\widetilde{\mathcal {M}}^{m}_{p}$ with the property that, on its support, $\textrm{max}_2\{ \langle j_1\rangle ,\dots , \langle j_p\rangle ,\langle j\rangle \}\sim \textrm{max}\{ \langle j_1\rangle ,\dots , \langle j_p\rangle ,\langle j\rangle \}$ is actually a *smoothing* operator in $\widetilde{\mathcal {R}}^{-\varrho }_{p}$ for *any* $\varrho \ge 0$ satisfying (2.32) with $\mu \leadsto \mu +m+\varrho$ and $m \leadsto -\varrho$.

$\bullet$ The Definition 2.6 of smoothing operators is modeled to gather remainders which satisfy either the property $\max _2 (n_1, \ldots , n_{p+1}) \sim \max (n_1, \ldots , n_{p+1})$ or arise as remainders of compositions of paradifferential operators, see Proposition 2.8 below, and thus have a fixed order $\varrho$ of regularization.

**Composition theorems.** Let $D_{x}:=\frac{1}{\textrm{i}}\partial _{x}$. The following is Definition 3.11 in [9].

#### Definition 2.7

*(Asymptotic expansion of composition symbol)* Let $\varrho \ge 0$, $m,m'\in {{\mathbb {R}}}$, $r>0$. Consider symbols $a \in \Sigma \Gamma ^{m}_{p}[r]$ and $b\in \Sigma \Gamma ^{m'}_{p'}[r]$, $p,p' \in \{0,2\}$. For *U* in $B_{{\mathtt s}}(I;r)$ we define, for $\varrho < {\mathtt s}- s_0$, the symbol

2.38 $$\begin{aligned} (a\#_{\varrho } b)(U;x,\xi ):= \sum _{k=0}^{\varrho } \frac{1}{2^k} \sum _{\ell +\beta =k} \frac{(-1)^{\beta }}{\ell ! \beta !} (\partial _\xi ^\ell D_x^\beta a ) \cdot (\partial _\xi ^\beta D_x^\ell b )(U;x,\xi ) \ .\qquad \end{aligned}$$

$\bullet$ The symbol $a\#_{\varrho } b$ belongs to $\Sigma \Gamma ^{m+m'}_{p+p'}[r]$.

$\bullet$ We have that $a\#_{\varrho }b =ab+\frac{1}{2 \textrm{i}}\{a,b\}$ up to a symbol in $\Sigma \Gamma ^{m+m'-2}_{p+p'}[r]$, where

2.39 $$\begin{aligned} \{a,b\} := \partial _{\xi }a\partial _{x}b -\partial _{x}a\partial _{\xi }b \in \Sigma \Gamma ^{m+m'-1}_{p+p'}[r] \end{aligned}$$

denotes the Poisson bracket. Moreover if $a\in \Gamma ^m_{W^{N,\infty }}$ and $b\in \Gamma ^{m'}_{W^{N,\infty }}$ then $\{a,b\}\in \Gamma ^{m+m'-1}_{W^{N-1,\infty }}$ with estimate

2.40 $$\begin{aligned} | \{a,b\} |_{m+m'-1, W^{N-1,\infty }, n} \lesssim | a |_{m, W^{N,\infty }, n+1}| b |_{m', W^{N,\infty }, n+1}. \end{aligned}$$

$\bullet$ Due to (2.18), the symbol $a\#_\varrho b$ does not contain symbols of odd homogeneity.

$\bullet$ $\overline{a^\vee } \#_{\varrho } \overline{b^\vee } = \overline{a \#_{\varrho } b}^\vee$ where $a^\vee$ is defined in (2.24).

The following proposition is proved in [13, Theorem A.8] and [9, Proposition 3.12]:

#### Proposition 2.8

(Composition of Bony-Weyl operators) Let $m, m' \in {{\mathbb {R}}}$, $p,p' \in \{0,2\}$, $\varrho \ge 0$ and $r >0$.

(*i*) Let $$a \in \Gamma ^m_{W^{\varrho , \infty }}$$, $$b \in \Gamma ^{m'}_{W^{\varrho , \infty }}$$. Then

$$\begin{aligned} \textrm{Op}^{\!\scriptscriptstyle BW}\!\left( a\right) \textrm{Op}^{\!\scriptscriptstyle BW}\!\left( b\right)&= \textrm{Op}^{\!\scriptscriptstyle BW}\!\left( a\#_\varrho b\right) + R(a,b), \end{aligned}$$

where the linear operator $R(a,b):{H}^s \rightarrow {H}^{s-(m+m')+\varrho }$, $\forall s \in {{\mathbb {R}}}$, satisfies, for some $N=N(\varrho ) >0$,

2.41 $$\begin{aligned} \Vert R(a,b)u \Vert _{{s -(m+m') +\varrho }} \lesssim \left( \left| a \right| _{m, W^{\varrho , \infty }, N} \, \left| b \right| _{m', L^\infty , N} + \left| a \right| _{m, L^\infty , N} \, \left| b \right| _{m', W^{\varrho , \infty }, N} \right) \Vert u \Vert _{s} \ . \end{aligned}$$

One can take that $N(2) = 7$.

(*ii*) Let $a\in \Sigma {\Gamma }^{m}_p[r]$, $b\in \Sigma {\Gamma }^{m'}_{p'}[r]$. Then

$$\begin{aligned} \textrm{Op}^{\!\scriptscriptstyle BW}\!\left( a(U;x,\xi )\right) \circ \textrm{Op}^{\!\scriptscriptstyle BW}\!\left( b(U;x,\xi )\right) = \textrm{Op}^{\!\scriptscriptstyle BW}\!\left( (a\#_{\varrho } b)(U;x,\xi )\right) + R(U), \end{aligned}$$

where *R*(*U*) are smoothing operators in $\Sigma {\mathcal {R}}^{-\varrho +m+m'}_{p+p'}[r]$.

$\bullet$ Let $a(U) \in \Sigma \Gamma ^m_{p}[r]$ and $b(U) \in \Sigma \Gamma ^{m'}_{p'}[r]$, with the notation in (2.27), one has that

2.42 $$\begin{aligned} \begin{aligned}&\left[ \textrm{Op}^{\scriptscriptstyle BW}_{\texttt {out}}\!\left( b\right) ,\textrm{Op}^{\scriptscriptstyle BW}_{\texttt {vec}}\!\left( a\right) \right] = \textrm{Op}^{\scriptscriptstyle BW}_{\texttt {out}}\!\left( b\#_\varrho \overline{a^\vee }- a\#_\varrho b\right) + R(U)\\&\left[ \textrm{Op}^{\scriptscriptstyle BW}_{\texttt {out}}\!\left( a\right) ,\textrm{Op}^{\scriptscriptstyle BW}_{\texttt {out}}\!\left( b\right) \right] = \textrm{Op}^{\scriptscriptstyle BW}_{\texttt {vec}}\!\left( a\#_\varrho \overline{b^\vee }- b\#_\varrho \overline{a^\vee }\right) + R(U)\\&\left[ \textrm{Op}^{\scriptscriptstyle BW}_{\texttt {vec}}\!\left( a\right) ,\textrm{Op}^{\scriptscriptstyle BW}_{\texttt {vec}}\!\left( b\right) \right] = \textrm{Op}^{\scriptscriptstyle BW}_{\texttt {vec}}\!\left( a\#_\varrho {b}- b\#_\varrho {a}\right) + R(U) \end{aligned}, \end{aligned}$$

where *R*(*U*) are real-to-real matrices of smoothing operators in $\Sigma {{\mathcal {R}}}_{p+p'}^{-\varrho + m'+m}[r]$.

We conclude this section with the paralinearization of the product (see [9, Lemma 7.2]).

#### Lemma 2.9

(Bony paraproduct decomposition) Let *f*, *g*, *h* be functions in $H^{\sigma }({{\mathbb {T}}};{{\mathbb {C}}})$ with ${\sigma }>\frac{1}{2}$. Then

$$\begin{aligned} fgh = \textrm{Op}^{\!\scriptscriptstyle BW}\!\left( f g \right) h + \textrm{Op}^{\!\scriptscriptstyle BW}\!\left( f h \right) g+\textrm{Op}^{\!\scriptscriptstyle BW}\!\left( g h \right) f + R_1(f, g )h+ R_2(f ,h )g+R_3(g, h )f \end{aligned}$$

where for $$j=1, 2, 3 $$, $$R_j$$ is a homogeneous smoothing operator in $$ {\widetilde{\mathcal {R}}}^{-\varrho }_{1}$$ for any $$ \varrho \ge 0$$.

**Composition of** *m*-**operators.** The next lemma, which is a consequence of Proposition 2.15 (items (*ii*) and (*iv*)) in [13], shall be used below.

#### Lemma 2.10

Let $m,m', m_0\in {{\mathbb {R}}}$, $\varrho \ge 0$, $r>0$, $p \in \{0,2\}$. Let *M*(*U*) be a real-to-real matrix of *m*-operators in $\Sigma \mathcal {M}_2^{m}[r]$, $\textbf{F}(U)$ be a real-to-real matrix of 0-operators $\mathcal {M}^0_{\ge 0}[r]$ and $\texttt{p}(\xi )$ be a matrix of Fourier multipliers in ${\widetilde{\Gamma }}_0^{m_0}$. Then

1. If *c*(*U*) is a 2-homogeneous symbol in ${\widetilde{\Gamma }}_2^{m'}$ and $c_{\ge 4}(U)$ is a non-homogeneous symbol in $\Gamma _{\ge 4}^m[r]$,
  $$\begin{aligned} b_2(U;x,\xi ):= c(-\textrm{i}\texttt{p}(D)U;x,\xi ), \quad \text {and} \quad {\left\{ \begin{array}{ll} b_{\ge 4}(U;x,\xi ):= c(M(U)U,U;x,\xi )\\ b'_{\ge 4}(U;x,\xi ):= c_{\ge 4}(\textbf{F}(U)U;x,\xi ) \end{array}\right. } \end{aligned}$$
  are symbols respectively in ${\widetilde{\Gamma }}^{m'}_{2}$ and $\Gamma _{\ge 4}^{m'}[r']$ for some $r'>0$;
2. If *Q*(*U*) is a 2-homogeneous smoothing operator in $\widetilde{\mathcal {R}}_2^{-\varrho }$,
  $$\begin{aligned} {\widetilde{R}}_2(U):= &   Q(-\textrm{i}\texttt{p}(D)U, U )\in \widetilde{\mathcal {R}}_2^{-\varrho +\max \{0,m_0\}} \quad \text {and} \quad \\ R_{\ge 4}(U):= &   Q(M(U)U,U)\in \mathcal {R}^{-\varrho +\max \{0,m\}}_{\ge 4}[r];\end{aligned}$$
3. If $R({U}) \in \Sigma {{\mathcal {R}}}^{-\varrho }_{ 2}[ r]$ and $\textsf{a}( U;x, \xi )\in \Sigma \Gamma ^m_{ 2}[ r]$, $\varrho \ge m$, then
  $$\begin{aligned} R({U})\circ \textrm{Op}^{\!\scriptscriptstyle BW}\!\left( \textsf{a}({U;x, \xi }) \right) \in {{\mathcal {R}}}^{-\varrho + m}_{\ge 4}[ r],  &   \textrm{Op}^{\!\scriptscriptstyle BW}\!\left( \textsf{a}( U;x, \xi ) \right) \circ R( U) \in {{\mathcal {R}}}^{-\varrho + m}_{\ge 4}[ r]. \end{aligned}$$
4. If *M* is in $\Sigma \mathcal {M}^{m}_{p}[r]$ and $M'$ is in $\Sigma \mathcal {M}^{m'}_{p'}[r]$ then the composition $M\circ M'$ is in $\Sigma \mathcal {M}^{m+\max (m',0)}_{p+p'}[r]$.
5. If *M*(*U*) is in ${\mathcal {M}}_{\ge 4}^{m}[r]$, then $M(\textbf{F}(U)U)$ is in $\mathcal {M}^{m}_{\ge 4}[r']$ for some $r'>0$.

### Admissible transformations

In this section we introduce a class of *U*-dependent transformations, that we call *admissible*, that have three properties: (*i*) they are bounded as maps on Sobolev spaces of sufficiently high regularity, (*ii*) they are differentiable with respect to the internal variable *U* and (*iii*) their differential may lose *m*-derivatives in the external variable, but gain $\varrho$-derivatives in the internal one. Examples are flows of paradifferential and smoothing operators; see Lemmas 2.16 and 2.17.

#### Definition 2.11

*(Admissible transformations)* Let $r>0$, $m, \varrho \ge 0$. We say that a real-to-real matrix $\textbf{F}(U)$ of non-homogeneous 0-operators in ${{\mathcal {M}}}_{\ge 0}^0[r]$ is an *m*-*admissible transformation* of *gain* $\varrho$ if the following holds:

(i) **Linear invertibility:** $\textbf{F}(U)$ is linearly invertible and its inverse $\textbf{F}(U)^{-1}$ is a real-to-real matrix of non-homogeneous 0-operators in ${{\mathcal {M}}}_{\ge 0}^{{0}}[r]$ satisfying the following: there exists $s_0>0$ such that for any $s\ge s_0 + \varrho$ there is a constant $C:=C_{s}>0$ and $r=r_s >0$ such that for any $U\in B_{s_0,{{\mathbb {R}}}}(r)\cap {H^{s-\varrho }_{{\mathbb {R}}}({{\mathbb {T}}};{{\mathbb {C}}}^2)}$ and $V \in H^s_{{\mathbb {R}}}({{\mathbb {T}}};{{\mathbb {C}}}^2)$ one has
  2.43 $$\begin{aligned}&\Vert \textbf{F}(U)V\Vert _{s}+ \Vert \textbf{F}^{-1}(U)V\Vert _{s}\le C (\Vert V \Vert _s + \Vert U\Vert _{s-\varrho }\Vert U\Vert _{s_0}\Vert V\Vert _{s_0}) \ . \end{aligned}$$
(ii) **Expansion:** $\textbf{F}(U) - \textrm{Id}$ is a matrix of *m*-operators in $\Sigma {{\mathcal {M}}}^m_2[r]$ expanding as
  2.44 $$\begin{aligned} \textbf{F}(U) = \textrm{Id}+ \textbf{F}_{2}(U)+ \textbf{F}_{\ge 4}(U), \quad \textbf{F}_{2}(U)\in {\widetilde{\mathcal {M}}}^m_2, \quad \textbf{F}_{\ge 4}(U) \in \mathcal {M}_{\ge 4}^m[r].\nonumber \\ \end{aligned}$$
(iii) **Derivative:** there is $s_0\ge 0$ such that for any $\sigma \ge s_0 + \varrho$, the map
  $$\begin{aligned} B_{\sigma -\varrho , {{\mathbb {R}}}}(r) \ni U \mapsto \textbf{F}(U) \in {{\mathcal {L}}}\big (H^{\sigma +m}_{{\mathbb {R}}}({{\mathbb {T}}},{{\mathbb {C}}}^2), \, H^{\sigma }_{{\mathbb {R}}}({{\mathbb {T}}},{{\mathbb {C}}}^2)\big ) =: X^{\sigma ,m} \end{aligned}$$
  is differentiable. Moreover its differential $\textrm{d}_U \textbf{F}(U)$ satisfies the quantitative bound: there are $C=C_{\sigma }>0$, $r'=r'({\sigma })>0$ such that for any $U \in B_{\sigma -\varrho ,{{\mathbb {R}}}}(r')$ and ${\hat{U}} \in H^{\sigma -\varrho }_{{\mathbb {R}}}({{\mathbb {T}}};{{\mathbb {C}}}^2)$
  2.45 $$\begin{aligned} \Vert \textrm{d}_U \textbf{F}(U)[{\widehat{U}}] \Vert _{X^{{\sigma },m}} \le C \Vert U \Vert _{\sigma -\varrho } \Vert {\hat{U}}\Vert _{\sigma -\varrho } \ . \end{aligned}$$
  Moreover, for any $s\ge s_0+m$, there is $C:=C_{s}>0$ such that for any $U\in B_{s_0,{{\mathbb {R}}}}(r)\cap H^{s}_{{\mathbb {R}}}({{\mathbb {T}}};{{\mathbb {C}}}^2)$, $Z, {\hat{U}} \in H^{s}_{{\mathbb {R}}}({{\mathbb {T}}};{{\mathbb {C}}}^2)$ one has
  2.46 $$\begin{aligned} \begin{aligned} \Vert&\left( \textrm{d}_U \textbf{F}(U)[{\hat{U}}] - \textrm{d}_U \textbf{F}_2(U)[{\hat{U}}]\right) Z\Vert _{s-m}=\Vert \textrm{d}_U \textbf{F}_{\ge 4}(U)[{\hat{U}}] Z \Vert _{s-m}\\&\le C \left( \Vert U\Vert _{s_0}^3\Vert {\hat{U}}\Vert _{s_0}\Vert Z\Vert _s + \Vert U\Vert _{s_0}^3\Vert {\hat{U}}\Vert _{s}\Vert Z\Vert _{s_0} + \Vert U\Vert _{s} \Vert U\Vert _{s_0}^2 \Vert {\hat{U}}\Vert _{s_0}\Vert Z\Vert _{s_0} \right) . \end{aligned}\nonumber \\ \end{aligned}$$

#### Remark 2.12

(1) Compared to *m*-operators in $\mathcal {M}_{\ge 0}^0[r]$, admissible transformations exhibit a gain of $\varrho$ derivatives in the internal variable *U*; see the second term in estimate (2.43) and compare it with (2.33) for $p = 0$. This additional gain will be verified since the admissible transformations we consider are linear flows generated by either paradifferential operators or smoothing operators. In both cases, the internal variable gains derivatives with respect to the external one.

(2) Thanks to the bound in (2.43), $\textbf{F}(U)$ conjugates any matrix $B_{\ge 4}(U)$ of 0-operators in $\mathcal {M}^0_{\ge 4}[r]$ into another matrix of 0-operators in $\mathcal {M}^0_{\ge 4}[r]$, namely $\textbf{F}(U) B_{\ge 4}(U) \textbf{F}(U)^{-1}$ is a matrix of 0-operators in $\mathcal {M}^0_{\ge 4}[r]$.

(3) Property (*ii*) implies that

$$\begin{aligned}&\Vert \left[ \textbf{F}(U)-\textrm{Id}\right] V\Vert _{s-m}+ \Vert \left[ \textbf{F}^{-1}(U)-\textrm{Id}\right] V\Vert _{s-m}\le C \Vert U\Vert _{s_0}^2 \Vert V \Vert _{s}+{\Vert U\Vert _{s_0}\Vert U\Vert _s \Vert V\Vert _{s_0}} \ \end{aligned}$$

and that

2.47 $$\begin{aligned} \Vert \textrm{d}_U \textbf{F}_2(U)[{\hat{U}}]V \Vert _{{s-m}} \lesssim _s \Vert U \Vert _{{s_0}} \Vert {\hat{U}} \Vert _{{s_0}} \Vert V \Vert _{ s}+ \Vert U \Vert _{{s_0} }\Vert {\hat{U}} \Vert _{s} \Vert V \Vert _{{s_0}} + \Vert U \Vert _{{s}} \Vert {\hat{U}} \Vert _{{s_0}} \Vert V \Vert _{s_0} \ .\nonumber \\ \end{aligned}$$

(4) The expansion (2.44) for $\textbf{F}(U)$ implies the corresponding expansion for $\textbf{F}(U)^{-1}$:

$$\begin{aligned} \textbf{F}(U)^{-1}=\textrm{Id}- \textbf{F}_{2}(U)+ \breve{\textbf{F}}_{\ge 4}(U), \end{aligned}$$

where $\breve{\textbf{F}}_{\ge 4}(U):= -\textbf{F}(U)^{-1}\textbf{F}_{\ge 4}(U) + \textbf{F}(U)^{-1}[\textbf{F}(U)-\textrm{Id}] \textbf{F}_2(U)$ is a real-to-real matrix of 2*m*-operators in $\mathcal {M}_{\ge 4}^{2m}[r]$.

We now prove that admissible transformations are closed by composition.

#### Lemma 2.13

Let $\textbf{F}^{(1)}(U)$ be $m_1$-admissible with gain $\varrho _1$ and $\textbf{F}^{(2)}(U)$ be $m_2$-admissible with gain $\varrho _2$. If $m_1 \le \varrho _2$, then the composition $\textbf{F}^{(1)}(U)\textbf{F}^{(2)}(U)$ is a $m_1+m_2$-admissible transformation with gain $\varrho := \min (\varrho _2 - m_1, \varrho _1)$.

#### Proof

We set $m:=m_1+m_2$. (*i*) and (*ii*) follows by the composition properties of *m*-operators, see Lemma 2.10-4, and by applying twice estimate (2.43) and using also $\varrho \le \min \{ \varrho _1,\varrho _2\}$. Moreover we have the expansion

$$\begin{aligned} \textbf{F}^{(1)}(U)\textbf{F}^{(2)}(U)=\textrm{Id}+\textbf{F}^{(1)}_2(U)+\textbf{F}^{(2)}_2(U)+\textbf{F}^{(1,2)}_{\ge 4 }(U), \end{aligned}$$

where $$\textbf{F}^{(1,2)}_{\ge 4 }(U)= \textbf{F}^{(1)}_{\ge 4 }(U)+\textbf{F}^{(2)}_{\ge 4 }(U)+ \left( \textbf{F}^{(1)}_2(U)+\textbf{F}^{(1)}_{\ge 4 }(U)\right) \left( \textbf{F}^{(2)}_2(U)+\textbf{F}^{(2)}_{\ge 4 }(U)\right) \in \mathcal {M}^{m}_{\ge 4}[r]$$.

(*iii*) Set $s_0:= s_0^{(1)}+s_0^{(2)}$ with $s_0^{(j)}$, $j=1,2$, the regularity threshold in property (*iii*) for $\textbf{F}^{(j)}$. We first prove that, for any $\sigma \ge s_0+\varrho$, $U \mapsto \textbf{F}^{(1)}(U) \textbf{F}^{(2)}(U)$ is differentiable at $U \in B_{\sigma -\varrho , {{\mathbb {R}}}}(r)$, $r>0$ sufficiently small, and its differential is given by

2.48 $$\begin{aligned} \textrm{d}_U \big ( \textbf{F}^{(1)}(U) \textbf{F}^{(2)}(U) \big )[{\widehat{U}}] = (\textrm{d}_U \textbf{F}^{(1)}(U)[{\widehat{U}}]) \, \textbf{F}^{(2)}(U) + \textbf{F}^{(1)}(U) (\textrm{d}_U \textbf{F}^{(2)}(U)[{\widehat{U}}]). \nonumber \\ \end{aligned}$$

Indeed fix $U\in B_{\sigma -\varrho , {{\mathbb {R}}}}(r)$, take ${\widehat{U}}$ with $\Vert {\hat{U}} \Vert _{{\sigma }-\varrho } \ll r$ and set that

$$\begin{aligned} \textbf{Q}(U,{\hat{U}}):=&\textbf{F}^{(1)}(U+ {\hat{U}})\textbf{F}^{(2)}(U+ {\hat{U}})\\&-\textbf{F}^{(1)}(U)\textbf{F}^{(2)}(U)- \left( (\textrm{d}_U \textbf{F}^{(1)}(U)[{\widehat{U}}]) \, \textbf{F}^{(2)}(U)+\textbf{F}^{(1)}(U) \, (\textrm{d}_U \textbf{F}^{(2)}(U)[{\widehat{U}}]) \right) \\ =&\left( \textbf{F}^{(1)}(U+ {\hat{U}})- \textbf{F}^{(1)}(U)- \textrm{d}_U \textbf{F}^{(1)}(U)[{\hat{U}}]\right) \textbf{F}^{(2)}(U+ {\hat{U}})\\&+ \textbf{F}^{(1)}(U) \left( \textbf{F}^{(2)}(U+{\hat{U}})-\textbf{F}^{(2)}(U)- \textrm{d}_U \textbf{F}^{(2)}(U)[{\hat{U}}]\right) \\&+\textrm{d}_U \textbf{F}^{(1)}(U)[{\hat{U}}]\left( \textbf{F}^{(2)}(U+{\hat{U}} )-\textbf{F}^{(2)}(U)\right) \\&= \textbf{Q}_1(U,{\hat{U}}) +\textbf{Q}_2(U,{\hat{U}}) + \textbf{Q}_3(U,{\hat{U}}). \end{aligned}$$

We show that, for $j=1,2,3$,

2.49 $$\begin{aligned} \Vert \textbf{Q}_j(U,{\hat{U}}) \Vert _{X^{{\sigma },m}}\lesssim \Vert {\hat{U}} \Vert _{{\sigma }-\varrho }^2 \end{aligned}$$

proving formula (2.48). Consider first $\textbf{Q}_1(U,{\hat{U}}) V$ with $V\in H^{{\sigma }+ m}_{{{\mathbb {R}}}}({{\mathbb {T}}}, {{\mathbb {C}}}^2)$. Using the differentiability of $\textbf{F}^{(1)}(U)$, estimate (2.43) for $\textbf{F}^{(2)}(U+ {\hat{U}})$ and that $\varrho =\min ( \varrho _2 - m_1, \varrho _1)$ we get that

$$\begin{aligned} \Vert \textbf{Q}_1(U,{\hat{U}}) V \Vert _{{\sigma }}&\lesssim \Vert \textbf{F}^{(1)}(U+ {\hat{U}}) - \textbf{F}^{(1)}(U)- \textrm{d}_U \textbf{F}^{(1)}(U)[{\hat{U}}] \Vert _{X^{{\sigma },m_1}} \\  &\qquad \Vert \textbf{F}^{(2)}(U+ {\hat{U}})V \Vert _{\sigma +m_1}\\&\lesssim \Vert {\hat{U}}\Vert _{{\sigma }-\varrho _1}^2 (\Vert V\Vert _{{\sigma }+m_1}+ \Vert U+ {\hat{U}}\Vert _{{\sigma }-(\varrho _2-m_1)}\Vert U+ {\hat{U}}\Vert _{s_0}\Vert V\Vert _{s_0})\\&{\lesssim } \Vert {\hat{U}}\Vert _{{\sigma }-\varrho }^2 \Vert V\Vert _{{\sigma }+m_1}, \end{aligned}$$

proving (2.49) for $j=1$ as $m \ge m_1$. We now prove the estimate for $j=2$. Using (2.43) and the differentiability of $\textbf{F}^{(2)}$, we get

$$\begin{aligned} \Vert \textbf{Q}_2(U,{\hat{U}}) V \Vert _{{\sigma }} \lesssim&\Vert \left( \textbf{F}^{(2)}(U+{\hat{U}})-\textbf{F}^{(2)}(U)- \textrm{d}_U \textbf{F}^{(2)}(U)[{\hat{U}}]\right) V \Vert _{{\sigma }}\\&+ \Vert U \Vert _{{\sigma }-\varrho _1}\Vert U\Vert _{s_0} \Vert \left( \textbf{F}^{(2)}(U+{\hat{U}})-\textbf{F}^{(2)}(U)- \textrm{d}_U \textbf{F}^{(2)}(U)[{\hat{U}}]\right) V\Vert _{s_0} \\ \lesssim&\Vert {\hat{U}}\Vert _{{\sigma }-\varrho _2}^2 \Vert V\Vert _{{\sigma }+m_2}+ \Vert U\Vert _{{\sigma }-\varrho _1}\Vert U\Vert _{s_0}\Vert {\hat{U}}\Vert _{{\sigma }-\varrho _2}^2\Vert V\Vert _{{\sigma }+m_2}\\&{\lesssim } \Vert {\hat{U}}\Vert _{{\sigma }-\varrho }^2 \Vert V\Vert _{{\sigma }+m}, \end{aligned}$$

also proving that (2.49) for $j=2$, Consider now $j=3$. Applying first (2.45) for $\textrm{d}_U \textbf{F}^{(1)}(U)[{\hat{U}}]$ with $m \leadsto m_1$, then writing $\textbf{F}^{(2)}(U + {\hat{U}}) - \textbf{F}^{(2)}(U) = \int _0^1 \textrm{d}_U \textbf{F}^{(2)}(U + \tau {\hat{U}})[{\hat{U}}] \textrm{d}\tau$ and using (2.45) for $\textrm{d}_U \textbf{F}^{(2)}(U+\tau {\hat{U}})[{\hat{U}}]$, $\tau \in [0,1]$ with $m\leadsto m_2$ and ${\sigma }\leadsto {\sigma }+m_1$ we get

$$\begin{aligned} \Vert \textbf{Q}_3(U,{\hat{U}})V \Vert _{{\sigma }} {\lesssim }&\Vert U \Vert _{{\sigma }-\varrho _1}\Vert {\hat{U}} \Vert _{{\sigma }-\varrho _1}\Vert \textbf{F}^{(2)}(U+{\hat{U}})V-\textbf{F}^{(2)}(U)V \Vert _{{\sigma }+m_1}\\ \lesssim&\Vert U \Vert _{{\sigma }-\varrho _1} \Vert {\hat{U}} \Vert _{{\sigma }-\varrho _1}\, \Vert U \Vert _{{\sigma }+m_1-\varrho _2}\, \Vert {\hat{U}}\Vert _{{\sigma }+m_1-\varrho _2}\, \Vert V\Vert _{{\sigma }+m}\\&{\lesssim } \Vert U \Vert _{{\sigma }-\varrho }^2 \Vert {\hat{U}} \Vert _{{\sigma }-\varrho }^2 \Vert V\Vert _{{\sigma }+m}, \end{aligned}$$

also proving that (2.49) for $j=3$. We conclude that (2.48) holds.

Next we show that $\textrm{d}_U \big ( \textbf{F}^{(1)}(U) \textbf{F}^{(2)}(U) \big )$ fulfills estimate (2.45). So fix ${\widehat{U}} \in H^{{\sigma }-\varrho }_{{\mathbb {R}}}({{\mathbb {T}}}, {{\mathbb {C}}}^2)$ and $V \in H^{{\sigma }+m}_{{\mathbb {R}}}({{\mathbb {T}}}, {{\mathbb {C}}}^2)$ and consider the first term in the right hand side of (2.48). We have

$$\begin{aligned}&\Vert (\textrm{d}_U \textbf{F}^{(1)}(U)[{\widehat{U}}]) \, \textbf{F}^{(2)}(U) V \Vert _{{\sigma }} {\mathop {\lesssim }\limits ^{(2.45)}} \Vert U \Vert _{{\sigma }-\varrho _1} \Vert {\widehat{U}} \Vert _{{\sigma }-\varrho _1} \Vert \textbf{F}^{(2)}(U) V \Vert _{{\sigma }+m_1} \\ {\mathop {\lesssim }\limits ^{(2.43)}}&\Vert U \Vert _{{\sigma }-\varrho _1} \Vert {\widehat{U}} \Vert _{{\sigma }-\varrho _1} \left( \Vert V \Vert _{{\sigma }+m_1} + \Vert U \Vert _{{\sigma }+ m_1-\varrho _2}\Vert U \Vert _{s_0}\Vert V \Vert _{s_0} \right) \\  &{\lesssim } \Vert U \Vert _{{\sigma }-\varrho } \Vert {\widehat{U}} \Vert _{{\sigma }-\varrho } \Vert V \Vert _{{\sigma }+m} \end{aligned}$$

The second term in (2.48) has an analogous estimate, proving (2.45).

Finally we prove the estimate (2.46). First we compute the differential

$$\begin{aligned} \begin{aligned} \textrm{d}_U \textbf{F}^{(1,2)}_{\ge 4 }(U)[{\hat{U}}]Z=&\textrm{d}_U \textbf{F}^{(1)}_{\ge 4 }(U)[{\hat{U}}]Z+\textrm{d}_U \textbf{F}^{(2)}_{\ge 4 }(U)[{\hat{U}}]Z\\&+\left( \textrm{d}_U\textbf{F}^{(1)}_2(U)[{\hat{U}} ]+\textrm{d}_U \textbf{F}^{(1)}_{\ge 4 }(U)[{\hat{U}}]\right) \left( \textbf{F}^{(2)}_2(U)+\textbf{F}^{(2)}_{\ge 4 }(U)\right) Z\\&+\left( \textbf{F}^{(1)}_2(U)+\textbf{F}^{(1)}_{\ge 4 }(U)\right) \left( \textrm{d}_U\textbf{F}^{(2)}_2(U)[{\hat{U}} ]+\textrm{d}_U \textbf{F}^{(2)}_{\ge 4 }(U)[{\hat{U}}]\right) Z. \end{aligned} \end{aligned}$$

Estimate (2.46) for $\textrm{d}_U \textbf{F}^{(1,2)}_{\ge 4 }(U)[{\hat{U}}]Z$ follows from the corresponding estimates for $\textrm{d}_U \textbf{F}^{(1)}_{\ge 4 }(U)[{\hat{U}}]Z$, $\textrm{d}_U \textbf{F}^{(2)}_{\ge 4 }(U)[{\hat{U}}]Z$ in (2.47) and (2.33)–(2.36) for $\textbf{F}_2^{(1)}(U)$, $\textbf{F}_2^{(2)}(U)$, $\textbf{F}_{\ge 4}^{(1)}(U)$ and $\textbf{F}_{\ge 4}^{(2)}(U)$. $\square$

Next we prove a local invertibility property of the nonlinear map $U \mapsto \textbf{F}(U)U$ when $\textbf{F}(U)$ is an admissible transformation.

#### Lemma 2.14

Let $\textbf{F}(U)$ be a *m*-admissible transformation with gain $\varrho \ge {\max \{ m, 1\}}$. Consider the nonlinear map $\mathcal {F}(U):= \textbf{F}(U)U$. The following holds true:

(i) There exists $s_0' \ge 0$ such that for any $s \ge s_0'$, the map ${{\mathcal {F}}}^{-1}$ is locally invertible: namely there is $r'>0$ and $\mathcal {F}^{-1}: B_{s_0',{{\mathbb {R}}}}(r')\cap H^{s}_{{\mathbb {R}}}({{\mathbb {T}}};{{\mathbb {C}}}^2)\rightarrow H^{s}_{{\mathbb {R}}}({{\mathbb {T}}};{{\mathbb {C}}}^2)$ such that
  $$\begin{aligned} \mathcal {F}\circ \mathcal {F}^{-1}(V)=V, \quad \mathcal {F}^{-1}\circ \mathcal {F}(U)=U, \quad \forall U,V\in B_{s_0',{{\mathbb {R}}}}(r') \ . \end{aligned}$$
(ii) One has ${{\mathcal {F}}}^{-1}(V) = \textbf{G}(V)V$ with $\textbf{G}(V)$ a matrix of non-homogeneous 0-operators in ${{\mathcal {M}}}_{\ge 0}^0[r']$ such that $\textbf{G}(V) - \textrm{Id}\in \Sigma {{\mathcal {M}}}_2^{2m}[r']$ for some $r' >0$ and expands as
  2.50 $$\begin{aligned} \textbf{G}(V) = \textrm{Id}- \textbf{F}_2(V) + \textbf{G}_{\ge 4}(V) \ , \quad \textbf{G}_{\ge 4}(V) \in {{\mathcal {M}}}^{2m}_{\ge 4}[r'] \ . \end{aligned}$$

#### Proof

Let $s_0, r >0$ the parameters given by Definition 2.11 associated to $\textbf{F}(U)$.

(*i*) Let $\sigma _0:= s_0 + \varrho$. We prove that there exists $r_1>0$ such that for any $V \in B_{\sigma _0 +m, {{\mathbb {R}}}}(r_1)$ there is a unique solution $U=\mathcal {F}^{-1}(V) \in B_{{\sigma }_0, {{\mathbb {R}}}}(r)$ of the equation $V= \mathcal {F}(U)= \textbf{F}(U)U$. Then we show that if $V \in H^{s}_{{\mathbb {R}}}({{\mathbb {T}}}, {{\mathbb {C}}}^2)$, $s > {\sigma }_0$, also $U \in H^{s}_{{\mathbb {R}}}({{\mathbb {T}}}, {{\mathbb {C}}}^2)$.

Exploiting the linear invertibility of $\textbf{F}(U)$, we recast $V=\textbf{F}(U)U$ as the fixed point problem

2.51 $$\begin{aligned} \mathcal {G}(U;V):=\textbf{F}(U)^{-1} V = U \ . \end{aligned}$$

First we show that for any $V \in B_{{\sigma }_0+m, {{\mathbb {R}}}}(r_1)$, the map $U \mapsto \mathcal {G}(U;V)$ is a contraction on the ball $B_{{\sigma }_0, {{\mathbb {R}}}}(r)$ provided $r_1>0$ is small enough.

${\underline{\mathcal {G}(U;V)\hbox { maps the ball into itself}.}}$ Let $V \in B_{{\sigma }_0+m, {{\mathbb {R}}}}(r_1)$ and $U \in B_{{\sigma }_0, {{\mathbb {R}}}}(r)$. It follows from (2.43) that

$$\begin{aligned} \Vert \mathcal {G}(U;V) \Vert _{{\sigma }_0} \le C \left( \Vert V \Vert _{{\sigma }_0} + \Vert U \Vert _{s_0}^2 \Vert V \Vert _{s_0} \right) \le C r_1 (1+r^2) \le r \end{aligned}$$

which is verified provided $r_1$ is sufficiently small.

${\underline{\mathcal {G}(U;V)\hbox { is a contraction.}}}$ Again let $V \in B_{{\sigma }_0+m, {{\mathbb {R}}}}(r_1)$ and $U_1, U_2 \in B_{{\sigma }_0, {{\mathbb {R}}}}(r)$. By (*iii*) one has

$$\begin{aligned}\textbf{F}(U_1) - \textbf{F}(U_2) = \int _0^1 \textrm{d}_U \textbf{F}(\tau U_1 + (1-\tau )U_2)[U_1 - U_2] \textrm{d}\tau \ , \end{aligned}$$

which applying $\textbf{F}(U_1)^{-1}$ to the left and $\textbf{F}(U_2)^{-1}$ to the right yields

$$\begin{aligned} \textbf{F}(U_1)^{-1} - \textbf{F}(U_2)^{-1} = -\int _0^1 \textbf{F}(U_1)^{-1} \, \textrm{d}_U \textbf{F}(\tau U_1 + (1-\tau )U_2)[U_1 - U_2]\, \textbf{F}(U_2)^{-1} \textrm{d}\tau . \end{aligned}$$

Exploiting such a formula, we get

$$\begin{aligned} \Vert \mathcal {G}(U_1;V)&- \mathcal {G}(U_2;V) \Vert _{{\sigma }_0} {\mathop {\le }\limits ^{(2.43)}} \sup _{\tau \in [0,1]}\Vert \textrm{d}_U \textbf{F}(\tau U_1 + (1-\tau )U_2)[U_1 - U_2]\, \textbf{F}(U_2)^{-1} V \Vert _{{\sigma }_0} \\&\ \ \ + \Vert U_1 \Vert _{s_0}^2 \sup _{\tau \in [0,1]}\Vert \textrm{d}_U \textbf{F}(\tau U_1 + (1-\tau )U_2)[U_1 - U_2]\, \textbf{F}(U_2)^{-1} V \Vert _{s_0} \\&{\mathop {\le }\limits ^{(2.45)}} C \left( \Vert U_1 \Vert _{s_0} + \Vert U_2 \Vert _{s_0} \right) \, \Vert U_1- U_2 \Vert _{s_0} \, \Vert F(U_2)^{-1}V \Vert _{{\sigma }_0 + m} \\&{\mathop {\le }\limits ^{(2.43)}} C \left( \Vert U_1 \Vert _{{\sigma }_0} + \Vert U_2 \Vert _{{\sigma }_0} \right) \, \Vert U_1- U_2 \Vert _{{\sigma }_0} \left( \Vert V \Vert _{{\sigma }_0+m} + \Vert U_2 \Vert _{s_0} \Vert U_2 \Vert _{s_0 + m } \Vert V \Vert _{s_0} \right) \\&\le C \left( \Vert U_1 \Vert _{{\sigma }_0} + \Vert U_2 \Vert _{{\sigma }_0} \right) \, \Vert U_1- U_2 \Vert _{{\sigma }_0} \, \Vert V \Vert _{{\sigma }_0+m} \le \frac{1}{2} \Vert U_1- U_2 \Vert _{{\sigma }_0}, \end{aligned}$$

where in the last step we chose $r_1>0$ small enough. By Banach fixed point theorem, there is a unique $U \in B_{{\sigma }_0,{{\mathbb {R}}}}(r)$ solving the fixed point problem (2.51), and so we set that

2.52 $$\begin{aligned} {{\mathcal {F}}}^{-1}(V) := U \ , \text{ so } \text{ that } {{\mathcal {G}}}({{\mathcal {F}}}^{-1}(V); V) = {{\mathcal {F}}}^{-1}(V) \ . \end{aligned}$$

Upgraded regularity. We now show that for any $s\ge {\sigma }_0+m$, if $V\in B_{{\sigma }_0+m,{{\mathbb {R}}}}(r_1) \cap H^s_{{\mathbb {R}}}({{\mathbb {T}}}, {{\mathbb {C}}}^2)$, then $\mathcal {F}^{-1}(V)$ belongs to $H^s_{{\mathbb {R}}}({{\mathbb {T}}}, {{\mathbb {C}}}^2)$ and

$$\begin{aligned} \Vert {{\mathcal {F}}}^{-1}(V)\Vert _s \le 2 C_s \Vert V\Vert _s\,. \end{aligned}$$

First, from the fixed point, $U:={{\mathcal {F}}}^{-1}(V) \in B_{{\sigma }_0,{{\mathbb {R}}}}(r)$. Now fix $\underline{n}\in {{\mathbb {N}}}$ so that $s \in ({\sigma }_0+\underline{n}\varrho , {\sigma }_0+\underline{n}\varrho +1]$. Then, from equation (2.51) and estimate (2.43), we get

$$\begin{aligned} \Vert U \Vert _{{\sigma }_0+n\varrho } \le C \big ( \Vert V \Vert _{{\sigma }_0+n\varrho } + \Vert U \Vert _{{\sigma }_0+(n-1)\varrho } \Vert U \Vert _{s_0}\Vert V \Vert _{s_0} \big ) \ , \quad n =1 , \ldots , \underline{n} \ . \end{aligned}$$

This shows that $U\in H^{{\sigma }_0+\underline{n}\varrho }_{{{\mathbb {R}}}}({{\mathbb {T}}};{{\mathbb {C}}}^2)$ and, using also that $U\in B_{s_0,{{\mathbb {R}}}}(r)$, $V\in B_{s_0+m,{{\mathbb {R}}}}(r_1)$, we get

2.53 $$\begin{aligned} \Vert U \Vert _{{\sigma }_0+n\varrho } \le C \Vert V \Vert _{{\sigma }_0+n\varrho }, \quad n=1,\ldots , \underline{n}. \end{aligned}$$

Finally, using $s-\varrho \le {\sigma }_0+\underline{n}\varrho <s$ and again that $U\in B_{s_0,{{\mathbb {R}}}}(r)$, $V\in B_{s_0+m,{{\mathbb {R}}}}(r_1)$, we deduce that

$$\begin{aligned} \Vert U\Vert _s = \Vert \textbf{F}^{-1}(U) V \Vert _s {\mathop {\le }\limits ^{(2.43)}}C\left( \Vert V \Vert _s+ \Vert U\Vert _{s-\varrho }\Vert U\Vert _{s_0}\Vert V\Vert _{s_0}\right) {\mathop {\le }\limits ^{(2.53)}} C \Vert V\Vert _{s}. \end{aligned}$$

So far we have shown that ${{\mathcal {F}}}\circ {{\mathcal {F}}}^{-1}(V) = V$ for any $V \in B_{s_0'}(r_1)$, where $s_0' = s_0 + \varrho + m$. Now we show that ${{\mathcal {F}}}^{-1}\circ {{\mathcal {F}}}(U) = U$ provided $U \in B_{s_0',{{\mathbb {R}}}}(r')$, with a smaller $r'$. First of all, note that ${{\mathcal {F}}}^{-1} \circ {{\mathcal {F}}}(U)$ solves the fixed point equation (2.51) with $V \leadsto {{\mathcal {F}}}(U)$ and $U \leadsto {{\mathcal {F}}}^{-1} \circ {{\mathcal {F}}}(U)$. When $\mathcal {F}(U) \in B_{s_0', {{\mathbb {R}}}}(r_1)$, the map ${{\mathcal {G}}}(\,\cdot ;{{\mathcal {F}}}(U))$ is a contraction. As a result, the associated fixed point problem admits a unique solution, which must therefore coincide with *U*. We prove now that $\mathcal {F}(U) \in B_{s_0', {{\mathbb {R}}}}(r_1)$. Indeed estimate (2.43), for some $C>1$, gives

$$\begin{aligned} \Vert \mathcal {F}(U)\Vert _{s_0'}=\Vert \textbf{F}\left( U\right) U\Vert _{s_0'}\le C\Vert U\Vert _{s_0'}\le r_1 \end{aligned}$$

for any $U\in B_{s_0',{{\mathbb {R}}}}(r_1/C)$. The thesis of item (*i*) follows by choosing $r':= r_1/C$.

(*ii*) It follows from (2.52) and (2.51)

2.54 $$\begin{aligned} {{\mathcal {F}}}^{-1}(V) = \textbf{G}(V) V \ , \quad \textbf{G}(V):= \textbf{F}({{\mathcal {F}}}^{-1}(V))^{-1}\in {{\mathcal {M}}}^0_{\ge 0}[r'] . \end{aligned}$$

Since by definition $r'=r_1/C\le r_1$, by the fixed point theorem $\mathcal {F}^{-1}(V)\in B_{s_0+\varrho ,{{\mathbb {R}}}}(r)$ for any $V \in B_{s_0',{{\mathbb {R}}}}(r')$. Then, since $\textbf{F}(U)^{-1}$ is a a real-to-real matrix of non-homogeneous 0-operators in ${{\mathcal {M}}}^0_{\ge 0}[r]$, it follows that $\textbf{G}(V)$ is a real-to-real matrix of non-homogeneous 0-operators in ${{\mathcal {M}}}^0_{\ge 0}[r']$ (with $s_0\leadsto s_0'$).

Next we show that $\textbf{G}(V)$ expands as in (2.50). Put ${\widetilde{{{\mathcal {F}}}}}^{-1}(V):= V - \textbf{F}_2(V)V$. Then, using the expansion ${{\mathcal {F}}}(U) = U + \textbf{F}_2(U)U + \textbf{F}_{\ge 4}(U)U$ and Lemma 2.10, we get

$$\begin{aligned} ({\widetilde{{{\mathcal {F}}}}}^{-1}\circ {{\mathcal {F}}})(U) = U + \textbf{F}'_{\ge 4}(U)U \ , \quad \text{ with } \textbf{F}'_{\ge 4}(U) \in {{\mathcal {M}}}^{2m}_{\ge 4}[r]. \ \end{aligned}$$

Substituting $U = {{\mathcal {F}}}^{-1}(V)$ and using (2.54) and Lemma 2.10, we obtain

$$\begin{aligned} {{\mathcal {F}}}^{-1}(V) = V - \textbf{F}_2(V) V + \textbf{G}_{\ge 4}(V) V, \quad \textbf{G}_{\ge 4}(V):= - \textbf{F}'_{\ge 4}({{\mathcal {F}}}^{-1}(V)) \textbf{G}(V) \in {{\mathcal {M}}}^{2m}_{\ge 4}[r'] \ . \end{aligned}$$

This proves the expansion in (2.50). $\square$

An immediate consequence of the above lemma is that the inverse $\mathcal {F}^{-1}$ of an admissible transformation $\mathcal {F}$ fulfills the estimate

2.55 $$\begin{aligned} \Vert \mathcal {F}^{-1}(V)\Vert _s\le C_s \Vert V\Vert _s, \quad \text {for any} \ V\in B_{s_0',{{\mathbb {R}}}}(r')\cap H^s({{\mathbb {T}}};{{\mathbb {C}}}^2) \ . \end{aligned}$$

We now show that the linear flows generated by two types of paradifferential operators are admissible transformations. Consider the flows

2.56 $$\begin{aligned}  &   {\left\{ \begin{array}{ll} \partial _\tau \Phi ^\tau (U)= G(\tau ,U)\Phi ^\tau (U)\\ \Phi ^0(U)=\textrm{Id}\end{array}\right. } \quad \text {where}\quad \nonumber \\ G(\tau ,U)= &   {\left\{ \begin{array}{ll} \textrm{Op}^{\scriptscriptstyle BW}_{\texttt {vec}}\!\left( \frac{\beta (U;x)}{1+\tau \beta _x(U;x)}\textrm{i}\xi \right) ,& \beta \in {\widetilde{\mathcal {F}}}_2^{{\mathbb {R}}}\ \ \text {or}\\ \textrm{Op}^{\scriptscriptstyle BW}_{\texttt {out}}\!\left( g(U;x,\xi )\right) ,&  g\in {\widetilde{\Gamma }}_2^0. \end{array}\right. } \end{aligned}$$

#### Remark 2.15

The map $\Phi ^\tau (U)$ is gauge invariant if the generator $G(U;\tau )$ is gauge invariant. Indeed $\Phi ^\tau ({\mathtt g}_\theta U){\mathtt g}_\theta$ and ${\mathtt g}_\theta \Phi (U)$ solve the same equation, thus coinciding.

The following lemma ensures that the flow map $\Phi ^\tau (U)$ generated by $G(\tau , U)$ is an admissible transformation for any $\tau \in [0,1]$:

#### Lemma 2.16

Let $\Phi ^\tau (U)$ be the flow map in (2.56). Fix an arbitrary $\varrho \ge 0$. Then

(*i*) if $G(\tau ,U)= \textrm{Op}^{\scriptscriptstyle BW}_{\texttt {vec}}\!\left( \frac{\beta (U;x)}{1+\tau \beta _x(U;x)}\textrm{i}\xi \right)$ then $\Phi ^\tau (U)$ is a 2-admissible transformation with gain $\varrho$;

(*ii*) if $G(\tau ,U)= \textrm{Op}^{\scriptscriptstyle BW}_{\texttt {out}}\!\left( g(U;x,\xi )\right)$ then $\Phi ^\tau (U)$ is a 0-admissible transformation with gain $\varrho$.

#### Proof

Along the proof we put $m=2$ if $G(\tau , U)$ is as in (*i*), and $m=0$ in case (*ii*).

It is classical that $\Phi ^\tau (U)$ is a matrix of 0-operators in ${{\mathcal {M}}}^0_{\ge 0}[r]$ as well as its linear inverse, see e.g. Lemma 3.16 of [14]. In particular, estimate (3.53) in [14] (with $k = K' =K=0$) gives that for any $U\in B_{s_0,{{\mathbb {R}}}}(r)\cap {H^{s-\varrho }_{{\mathbb {R}}}({{\mathbb {T}}};{{\mathbb {C}}}^2)}$ and $V \in H^s_{{\mathbb {R}}}({{\mathbb {T}}};{{\mathbb {C}}}^2)$, $\sup _{\tau \in [0,1]}\Vert \Phi ^\tau (U)^{\pm 1}V \Vert _s \le C \Vert V \Vert _s$, which clearly implies both (2.43) and the second of (2.33).

We now prove the expansion (2.44). First we expand

2.57 $$\begin{aligned} G(\tau ,U)=G_2(U)+ G_{\ge 4}(\tau ,U)= {\left\{ \begin{array}{ll} \textrm{Op}^{\scriptscriptstyle BW}_{\texttt {vec}}\!\left( {\beta (U;x)}\textrm{i}\xi \right) +G_{\ge 4}(\tau ,U)\\ \textrm{Op}^{\scriptscriptstyle BW}_{\texttt {out}}\!\left( g(U;x,\xi )\right) \, , \end{array}\right. } \end{aligned}$$

so the expansion of $\Phi ^\tau (U)$ reads as

$$\begin{aligned} \Phi ^\tau (U)= \textrm{Id}+ \tau G_2(U) + \textbf{F}_{\ge 4}(\tau , U), \quad \textbf{F}_{\ge 4}(\tau , U)\in \mathcal {M}^m_{\ge 4}[r]. \end{aligned}$$

We prove now (*iii*). First we claim that, for both choices of $G(\tau , U)$ in (2.56), there is $s_0 >0$ such that, for any $s\in {{\mathbb {R}}}$,

2.58 $$\begin{aligned}&\sup _{\tau \in [0,1]}\Vert \textrm{d}_U G(\tau ,U)[\widehat{U}]W \Vert _{s-\frac{m}{2}} \lesssim \Vert U \Vert _{s_0}\Vert \widehat{U} \Vert _{s_0}\Vert W \Vert _{s}, \end{aligned}$$

2.59 $$\begin{aligned}&\sup _{\tau \in [0,1]}\Vert \textrm{d}_U G_{\ge 4}(\tau ,U)[\widehat{U}]W \Vert _{s-\frac{m}{2}} \lesssim \Vert U \Vert _{s_0}^3\Vert \widehat{U} \Vert _{s_0}\Vert W \Vert _{s}. \end{aligned}$$

Assuming for the moment such properties, consider the differential $\textrm{d}_U \Phi ^\varsigma (U)[{\widehat{U}}]$. It fulfills the variational equation

$$\begin{aligned} {\left\{ \begin{array}{ll} \partial _\varsigma \textrm{d}_U \Phi ^\varsigma (U)[{\widehat{U}}] =G(\varsigma ,U) \textrm{d}_U \Phi ^\varsigma (U)[{\widehat{U}}] +\textrm{d}_U G(\varsigma ,U)[\widehat{U}] \Phi ^\varsigma (U), \\ \textrm{d}_U \Phi ^0(U)[{\widehat{U}}]=0 \, , \end{array}\right. } \end{aligned}$$

whose solution is given by the Duhamel formula

2.60 $$\begin{aligned} \textrm{d}_U \Phi ^\varsigma (U)[{\widehat{U}}] =&\Phi ^\varsigma (U) \int _0^\varsigma \Phi ^\tau (U)^{-1} \ \textrm{d}_U G(\tau ,U)[\widehat{U}]\ \Phi ^\tau (U)\, \textrm{d}\tau \nonumber \\ {\mathop {=}\limits ^{(2.57), (2.56)}}&\varsigma \textrm{d}_U G_2(U)[\widehat{U}]+\varsigma \int _0^\varsigma G(\theta ,U)\Phi ^\theta (U)\textrm{d}_U G_2(U)[\widehat{U}]\, \textrm{d}\theta \nonumber \\  &+ \Phi ^\varsigma (U)\int _0^\varsigma \textrm{d}_U G_{\ge 4}(\tau ,U)[\widehat{U}]\, \textrm{d}\tau \nonumber \\&+\Phi ^\varsigma (U) \int _0^\varsigma \int _0^\tau \Phi ^\theta (U)^{-1} \ \left[ \textrm{d}_U G(\tau ,U)[\widehat{U}], G(\theta ,U)\right] \ \Phi ^\theta (U)\,\textrm{d}\theta \,\textrm{d}\tau , \end{aligned}$$

where in the second equality we also used the expansion

$$\begin{aligned} \Phi ^\theta (U)^{-1}&\ \text {d}_U G(\tau ,U)[\widehat{U}]\ \Phi ^\theta (U)_{|\theta =\tau }\\ = \text {d}_U G(\tau ,U)[\widehat{U}] + \int _0^\tau&\Phi ^\theta (U)^{-1} \ \left[ \text {d}_U G(\tau ,U)[\widehat{U}], G(\theta ,U)\right] \ \Phi ^\theta (U)\,\text {d}\theta . \end{aligned}$$

Inserting estimates (2.58)–(2.59) in (2.60) and using (2.33) for $\Phi ^\varsigma (U)$ and (2.30) for $G(\tau , U)$, one checks that, for any ${\sigma }\ge s_0 + \varrho$,

$$\begin{aligned} \Vert \textrm{d}_U \Phi ^\varsigma (U)[{\widehat{U}}] V \Vert _{\sigma }\le C \Vert U \Vert _{s_0} \, \Vert {\widehat{U}} \Vert _{s_0} \Vert W \Vert _{{\sigma }+2} \le \Vert U \Vert _{{\sigma }-\varrho } \, \Vert {\widehat{U}} \Vert _{{\sigma }- \varrho } \Vert W \Vert _{{\sigma }+2} \end{aligned}$$

showing the validity of (2.45).

Similarly one checks that the term $\left( \textrm{d}_U\Phi ^\tau (U)[{\hat{U}}]- \tau \textrm{d}_U G_2(U)[{\hat{U}}]\right) W$ fulfills (2.46). We now prove (2.58)–(2.59). Consider first $G(\tau ,U)= \textrm{Op}^{\scriptscriptstyle BW}_{\texttt {out}}\!\left( g(U;x,\xi )\right)$, for which (2.59) is trivial (being $G_{\ge 4 }(\tau ,U)\equiv 0$). Since $g(U;\cdot )$ is homogeneous of degree 2,

$$\begin{aligned} \textrm{d}_U G(\tau ,U)[\widehat{U}]=\textrm{Op}^{\scriptscriptstyle BW}_{\texttt {out}}\!\left( 2 g(\widehat{U}, U;x,\xi )\right) =\textrm{d}_U G_2(U)[{\hat{U}}] \ , \end{aligned}$$

and (2.58) follows from Theorem 2.4.

Next we analyze the case $G(\tau ,U)= \textrm{Op}^{\scriptscriptstyle BW}_{\texttt {vec}}\!\left( \frac{\beta (U;x)}{1+\tau \beta _x(U;x)}\textrm{i}\xi \right)$. Its differential is given by

$$\begin{aligned} \textrm{d}_U G(\tau ,U)[\widehat{U}]&= 2\textrm{Op}^{\scriptscriptstyle BW}_{\texttt {vec}}\!\left( \beta (\widehat{U},U;x)\textrm{i}\xi \right) \\  &-{2\tau } \textrm{Op}^{\scriptscriptstyle BW}_{\texttt {vec}}\!\left( \frac{\beta (\widehat{U},U;x) \beta _x(U;x)}{1+\tau \beta _x(U;x)} + \frac{\beta (U;x)\beta _x(\widehat{U}, U;x)}{(1+\tau \beta _x(U;x))^2}\textrm{i}\xi \right) \\&= \textrm{d}_U G_2(U)[{\hat{U}}]+ \textrm{d}_U G_{\ge 4}(\tau , U)[{\hat{U}}] \end{aligned}$$

Now notice that $\beta (\widehat{U},U;x) \in {\widetilde{{{\mathcal {F}}}}}^{{\mathbb {R}}}_{2}$ and

$$\begin{aligned} {\mathtt b}(\tau , {\widehat{U}}, U):= \frac{\beta (\widehat{U},U;x) \beta _x(U;x)}{1+\tau \beta _x(U;x)} + \frac{\beta (U;x)\beta _x(\widehat{U}, U;x)}{(1+\tau \beta _x(U;x))^2} \in L^\infty ({{\mathbb {T}}}; {{\mathbb {R}}})\end{aligned}$$

with bound $\sup _{\tau \in [0,1]} \Vert {\mathtt b}(\tau , {\widehat{U}}, U) \Vert _{L^\infty } \lesssim \Vert \widehat{U} \Vert _{s_0}\Vert U \Vert _{s_0}^3.$ Then Theorem  2.4 gives (2.58) and (2.59). $\square$

Next we consider the flow map generated by a matrix of smoothing operators:

2.61 $$\begin{aligned} {\left\{ \begin{array}{ll} \partial _\tau \Phi ^\tau (U)= R(U)\Phi ^\tau (U)\\ \Phi ^0(U)=\textrm{Id}\end{array}\right. } \quad \text {where}\quad R(U) \in {\widetilde{{{\mathcal {R}}}}}^{-\varrho }_2 \ . \end{aligned}$$

#### Lemma 2.17

Let $\varrho >0$. The flow $\Phi ^\tau (U)$ in (2.61) is a 0-admissible transformation with gain $\varrho$.

#### Proof

Since $R(U) \in {\widetilde{{{\mathcal {R}}}}}^{-\varrho }_2$, for any $U \in { B_{s_0, {{\mathbb {R}}}}(r)}$ with $s_0>0$ sufficiently large the problem (2.61) admits a unique solution $\Phi ^{\tau }(U)$ fulfilling $\Vert \Phi ^\tau (U)V \Vert _{s_0} \le C \Vert V \Vert _{s_0}$ uniformly for $\tau \in [-1,1]$. We now prove that $\Phi ^\tau (U)$ fulfills (2.43). Let $s \ge s_0+ \varrho$, $U \in { B_{s_0, {{\mathbb {R}}}}(r_s)} \cap H^{s-\varrho }_{{{\mathbb {R}}}}({{\mathbb {T}}}, {{\mathbb {C}}}^2)$ with a sufficiently small $r_s>0$ and $V \in H^{s}_{{\mathbb {R}}}(\mathbb {T};{{\mathbb {C}}}^2)$. Then the integral formula $\Phi ^\tau (U) = \textrm{Id}+ \int _0^\tau R(U) \Phi ^{\tau '}(U) \textrm{d}\tau '$ and estimate (2.36) (with $m = -\varrho$ and $s \leadsto s-\varrho$) yield

$$\begin{aligned} \begin{aligned} \Vert \Phi ^\tau (U)V \Vert _{s}\le&C_s \left( \Vert V \Vert _{s } + \Vert V \Vert _{s_0} \Vert U \Vert _{s-\varrho } \Vert U \Vert _{s_0}\right) + C_s\sup _{\tau \in [-1,1]}\Vert \Phi ^\tau (U)V \Vert _{s-\varrho } \Vert U \Vert _{s_0}^2 . \end{aligned} \end{aligned}$$

Then, possibly shrinking $r_s$ so that $C_s r_s^2 < \frac{1}{2}$, we obtain

$$\begin{aligned} \sup _{\tau \in [-1,1]} \Vert \Phi ^\tau (U)V \Vert _{s}\le 2 C_s \left( \Vert V \Vert _{s } + \Vert V \Vert _{s_0} \Vert U \Vert _{s-\varrho } \Vert U \Vert _{s_0}\right) , \end{aligned}$$

proving (2.43). The rest of the proof follows along the same lines as the previous one. The algebraic expansion (2.60) holds with $G(\tau ,U)\leadsto R(U)$ and, since $\textrm{d}_U R(U)[{\widehat{U}}] = 2 R(U, {\widehat{U}})$, we replace (2.58) and (2.59) with the bound

$$\begin{aligned} \Vert \textrm{d}_U R(U)[{\widehat{U}}]W \Vert _\sigma \le C \Vert U \Vert _{{\sigma }-\varrho } \Vert {\widehat{U}} \Vert _{{\sigma }-\varrho } \Vert W \Vert _{{\sigma }-\varrho }, \end{aligned}$$

obtained from (2.36) with $m =- \varrho$ and $s-m \leadsto \sigma$. Then both (2.45) and (2.46) follow. $\square$

## Analysis of Weak Resonances

Equation (1.1) is Hamiltonian, with Hamiltonian function given by

$$\begin{aligned} \mathscr {H}(u) := \int _{{\mathbb {T}}}(|D|^\alpha u ) {\overline{u}} + \frac{\textrm{i}}{4} \int _{{\mathbb {T}}}|u|^2 \, ({\overline{u}} \, u_x - u {\overline{u}}_x) \, \textrm{d}x \ . \end{aligned}$$

Due to the gauge and translation invariance of equation (1.1), any sufficiently regular solution *u*(*t*) of (1.1) conserves the total mass and momentum, namely

3.1 $$\begin{aligned} \begin{aligned}&\mathscr {M}(u(t)) :=\frac{1}{2\pi } \Vert u(t) \Vert _{L^2}^2 \equiv \frac{1}{2\pi } \int _{{\mathbb {T}}}|u(t,x)|^2 \textrm{d}x = \sum _{k \in {{\mathbb {Z}}}} | u_k(t)|^2 = \mathscr {M}(u(0)) , \\&\mathscr {P}(u(t)) := \frac{1}{2\pi } \int _{{\mathbb {T}}}\textrm{i}(\partial _x u(t,x)) \, \overline{u(t,x)} \, \textrm{d}x = -\sum _{k \in {{\mathbb {Z}}}} k | u_k(t)|^2 = \mathscr {P}(u(0)) \ . \end{aligned} \end{aligned}$$

In view of this we introduce the new variable

$$\begin{aligned} v(t,x):= e^{\textrm{i}t \mathscr {P}(u(t))}u\big (t,x-\mathscr {M}(u(t))t \big ) \ . \end{aligned}$$

Clearly *v*(*t*, *x*) and *u*(*t*, *x*) have same Sobolev norms, same magnitude, mass and the momentum, i.e.

$$\begin{aligned} \Vert v(t, \cdot ) \Vert _{s} = \Vert u(t,\cdot ) \Vert _s \ , \quad \forall s \in {{\mathbb {R}}}\end{aligned}$$

and

$$\begin{aligned} |v(t,x)|= |u(t,x-\mathscr {M}(u(t))t )|, \quad \mathscr {M}(v(t))=\mathscr {M}(u(t)), \quad \mathscr {P}(v(t)) = \mathscr {P}(u(t)) \ , \end{aligned}$$

and one readily checks that $v(t,\cdot )$ fulfills the re-normalized equation

3.2 $$\begin{aligned} \partial _t v = - \textrm{i}|D|^\alpha v+ |v|^2 v_x - \mathscr {M}(v)v_x+ \textrm{i}\mathscr {P}(v)v \ . \end{aligned}$$

This is the equation that we shall consider from now on, and we will relabel $v\leadsto u$. Also (3.2) is a Hamiltonian PDE with Hamiltonian function

$$\begin{aligned} {\widetilde{\mathscr {H}}}(v):= \mathscr {H}(v)- \mathscr {M}(v)\mathscr {P}(v) \ . \end{aligned}$$

### Remark 3.1

The reason we renormalize equation (1.1) is that the vector field of (3.2) does not contain integrable resonant monomials of the form $|u_k|^2 u_\ell e^{\textrm{i}\ell x}$ with $k \ne \ell$. Although not strictly necessary, it simplifies the analysis of the resonant part of (3.2) in Lemma 3.4.

**Analysis of 4-waves interactions.** Denote by $\textsf{R}$ the subset of ${\mathcal {P}}_4$ (recall (2.8)) consisting in 4-waves resonant indexes, namely

3.3 $$\begin{aligned} \textsf{R}:= \left\{ ( {\jmath } , \mathbf {\sigma }) \in {\mathcal {P}}_4 :\quad \sigma _1 |j_1|^\alpha + \sigma _2 |j_2|^\alpha + \sigma _3 |j_3|^\alpha +\sigma _4 |j_4|^\alpha = 0 \right\} \ . \end{aligned}$$

When $\alpha \in (0,1)$ is irrational, one can expect the set $\textsf{R}$ to contain only integrable resonances, namely indexes of the form $\big ( (k, k, \ell , \ell ), (+, -, +, -) \big )$ with $k, \ell \in {{\mathbb {Z}}}$ and their permutations. For $\alpha$ rational, instead, nonintegrable resonances do exist in general: for example, when $\alpha = \frac{1}{2}$, one has the non-integrable Zakharov-Dyachenko resonances [73]. We do not care if such non-integrable resonances exist or not, since, as we discussed in the introduction, our energy cascades will be due to *quasi-resonances*, rather than exact resonances. What we really are interested in, is to study the resonances between frequencies in a fixed set $\Lambda$ and those in its complementary set, with at most two frequencies in $\Lambda ^c$.

We shall now study resonant sets with indexes constrained to belong to certain subsets.

### Definition 3.2

Given a set $\Lambda \subseteq {{\mathbb {Z}}}$ and $n \in \{0, \ldots , 4\}$, we denote by ${\mathcal {P}}_\Lambda ^{(n)}$ the elements of ${{\mathcal {P}}}_4$ (see (2.8)) having exactly *n* indexes *outside* the set $\Lambda$:

3.4 $$\begin{aligned} {\mathcal {P}}_\Lambda ^{(n)} := \{ ( j_1, j_2, j_3, j_4 , \mathbf {\sigma }) \in {\mathcal {P}}_4 :\text{ exactly } n \text{ indexes } \text{ among } j_1, j_2, j_3, j_4 \text{ are } \text{ outside } \Lambda \} \ .\nonumber \\ \end{aligned}$$

We denote by $\textsf{R}_\Lambda ^{(n)}$ the subset of ${\mathcal {P}}_\Lambda ^{(n)}$ made of 4-waves resonances: with ${\mathtt R}$ in (3.3),

3.5 $$\begin{aligned} \textsf{R}_\Lambda ^{(n)} := \{ ( j_1, j_2, j_3, j_4 , \mathbf {\sigma }) \in \textsf{R}:\text{ exactly } n \text{ indexes } \text{ among } j_1, j_2, j_3, j_4 \text{ are } \text{ outside } \Lambda \} \ .\nonumber \\ \end{aligned}$$

We shall now study in detail the sets $\textsf{R}_\Lambda ^{(n)}$, $n = 0,1,2$, when $\Lambda$ is given by

3.6 $$\begin{aligned} \Lambda := \{ -1, +1\} \ . \end{aligned}$$

### Lemma 3.3

Let $\Lambda$ in (3.6) and ${{\mathcal {P}}}^{(n)}_\Lambda$, $\textsf{R}^{(n)}_\Lambda$ defined in (3.4) and (3.5).

(i) The set ${\mathcal {P}}_\Lambda ^{(0)} \equiv \textsf{R}_\Lambda ^{(0)}$ and it contains only integrable resonances:
  3.7 $$\begin{aligned} \begin{aligned} \textsf{R}_\Lambda ^{(0)}&= \left\{ \big ( \pi (\texttt{k}, \texttt{k}, \ell , \ell ), \ \pi (+,-, +, -) \big ), :\texttt{k}, \ell \in \Lambda \ , \ \ \pi \in {{\mathcal {S}}}_4 \right\} \end{aligned} \end{aligned}$$
  and ${{\mathcal {S}}}_4$ is the symmetric group of permutations of four symbols.
(ii) The set $\textsf{R}_\Lambda ^{(1)} = \emptyset$. Moreover ${\mathcal {P}}_\Lambda ^{(1)}$ has finite cardinality and there exists $c >0$ such that
  3.8 $$\begin{aligned} ({\jmath }, \mathbf {\sigma }) \in {\mathcal {P}}_\Lambda ^{(1)} \quad \Rightarrow \quad \left| \sigma _1 |j_1|^\alpha + \sigma _2 |j_2|^\alpha + \sigma _3 |j_3|^\alpha +\sigma _4 |j_4|^\alpha \right| \ge c \ . \end{aligned}$$
(iii) The set
  3.9 $$\begin{aligned} \begin{aligned} \textsf{R}_\Lambda ^{(2)}&= \{ \big ( \pi (\texttt{k}, \texttt{k}, \ell , \ell ), \ \pi (+,-, +, -) \big ) :\ \mathtt k \in \Lambda , \ \ell \in \Lambda ^c , \ \pi \in {{\mathcal {S}}}_4 \} \ . \end{aligned} \end{aligned}$$
  Moreover there exists $c >0$ such that
  3.10 $$\begin{aligned} ({\jmath }, \mathbf {\sigma }) \in {\mathcal {P}}_\Lambda ^{(2)}\setminus \textsf {R}_\Lambda ^{(2)} \quad \Rightarrow \\ \left| \sigma _1 |j_1|^\alpha + \sigma _2 |j_2|^\alpha + \sigma _3 |j_3|^\alpha +\sigma _4 |j_4|^\alpha \right| \nonumber \ge&\frac{c}{\max \limits _{a=1,\ldots , 4} ( |j_a|)^{1-\alpha } } \ . \end{aligned}$$

### Proof

The gauge condition $\sum _{a=1}^4 \sigma _a = 0$ implies that exactly two $\sigma _a$’s are $+$, the other are −. So, up to permutation, we can always assume that $\sigma _1 = \sigma _3 = 1$ and $\sigma _2 = \sigma _4 = -1$.

(*i*) In this case all indexes $j_1, j_2, j_3, j_4 \in \Lambda$, so automatically $|j_1|^\alpha - |j_2|^\alpha + |j_3|^\alpha -|j_4|^\alpha = 0$, so ${\mathcal {P}}^{(0)}_\Lambda = \textsf{R}^{(0)}_\Lambda$. Next the momentum condition $j_1-j_2 +j_3-j_4 =0$ gives that either $j_1 = j_2 = \texttt{k}$, $j_3 =j_4 = \ell$, yielding $\Big ( (\texttt{k}, \texttt{k}, \ell , \ell ), (+,-,+,-)\Big )$, or $j_1 = j_4 = \texttt{k}$, $j_2 = j_3 = \ell$, yielding $\Big ( (\texttt{k},\ell ,\ell , \texttt{k}), (+,-,+,-)\Big )$, which is a permutation of the previous one.

(*ii*) We can always assume that $j_1, j_2, j_3 \in \Lambda$ and $j_4 \in \Lambda ^c$. Then the resonant condition $|j_1|^\alpha - |j_2|^\alpha + |j_3|^\alpha - |j_4|^\alpha$ reduces to $|j_3|^\alpha - |j_4|^\alpha$, for which we have the lower bound

$$\begin{aligned} \left| |j_3|^\alpha - |j_4|^\alpha \right| \ge {\left\{ \begin{array}{ll} 2^\alpha -1 , &  \text{ if } |j_4| \ge 2 \ , \\ 1 , &  \text{ if } j_4 = 0 \end{array}\right. } \ \ \ . \end{aligned}$$

This proves both $\textsf{R}_\Lambda ^{(1)} = \emptyset$ and (3.8).

(*iii*) We have two different cases.

Case I: W.l.o.g. assume $j_1, j_3 \in \Lambda$, $j_2, j_4 \in \Lambda ^c$. The momentum condition reads $j_1 + j_3 = j_2 + j_4$. We examine further subcases.

$$\bullet $$ If $$j_2 = j_4 = 0$$, then $$ \left| |j_1|^\alpha - |j_2|^\alpha + |j_3|^\alpha - |j_4|^\alpha \right| = 2$$.

$\bullet$ If $j_2 = 0$ and $j_4 \ne 0$, from the momentum condition we get $|j_4| \le 2$, so actually $j_4 = \pm 2$. Then $\left| |j_1|^\alpha - |j_2|^\alpha + |j_3|^\alpha - |j_4|^\alpha \right| = 2 - 2^\alpha >0$.

$\bullet$ If $j_2, j_4 \ne 0$, then $|j_2|, |j_4| \ge 2$. Then $\left| |j_1|^\alpha - |j_2|^\alpha + |j_3|^\alpha - |j_4|^\alpha \right| \ge 2(2^\alpha -1) >0$.

Hence in Case I there are no resonances and the lower bound (3.10) holds.

Case II: W.l.o.g. assume that $j_1, j_2 \in \Lambda$, $j_3, j_4 \in \Lambda ^c$. The momentum condition reads $j_1 - j_2 = j_4- j_3$. Again we examine further subcases.

$\bullet$ If $j_1=j_2 = {\mathtt k}\in \Lambda$, then, by the momentum, $j_3 = j_4 = \ell \in \Lambda ^c$ and they form an element of $\textsf{R}^{(2)}_\Lambda$. All other cases in (3.9) are obtained by permutations.

$\bullet$ If $j_1 \ne j_2$, then $j_4 = j_3 \pm 2$. Consider the “+” case, the other being analogous. The term $\left| |j_1|^\alpha - |j_2|^\alpha + |j_3|^\alpha - |j_4|^\alpha \right|$ reduces to

$$\begin{aligned} \begin{aligned} \left| |j_3+2|^\alpha - |j_3|^\alpha \right|&\ge {\left\{ \begin{array}{ll} 2^\alpha &  \text{ if } j_3 = 0 \text{ or } j_3 = -2 \\ 4^\alpha -2^\alpha &  \text{ if } j_3 = 2 \\ \dfrac{c_\alpha }{\max \big (|j_3|, \, |j_3+2|\big )^{1-\alpha }} &  \text{ if } |j_3|\ge 3 \end{array}\right. } \end{aligned} \end{aligned}$$

proving (3.10). $$\square $$

**Projection of cubic vector fields.** We introduce now projections of cubic vector fields on the sets ${\mathcal {P}}_\Lambda ^{(n)}$ and $\textsf{R}_\Lambda ^{(n)}$. Recall that any real-to-real cubic vector field *X*(*U*), translation and gauge invariant, expand in Fourier as (see (2.12))

3.11 $$\begin{aligned} X(U)^\sigma = \!\!\!\!\!\! \sum _{({\jmath }, k, \mathbf {\sigma }, - \sigma ) \in {\mathcal {P}}_4} \!\!\!\!\!\! X_{\ j_1, j_2, j_3, k }^{\sigma _1, \sigma _2, \sigma _3, \sigma } \ u_{j_1}^{\sigma _1} \, u_{j_2}^{\sigma _2} \, u_{j_3}^{\sigma _3}\, { e^{\textrm{i}\sigma k x }} \ , \quad X_{\ j_{\pi (1)}, \ldots ,j_{\pi (3)}, k}^{ \sigma _{\pi (1)}, \ldots , \sigma _{\pi (3)},\sigma } = X_{\ j_{1}, \ldots , j_{3}, k}^{ \sigma _{1}, \ldots , \sigma _{3},\sigma } \,\nonumber \\ \end{aligned}$$

for any permutation $\pi$ of $\{1, 2, 3 \}$. Given a subset $A \subseteq {\mathcal {P}}_4$, we denote by $\Pi _A X$ the vector field obtained restricting the indexes to belong to *A*, namely

3.12 $$\begin{aligned} (\Pi _A X)(U)^\sigma := \!\!\!\!\!\!\! \sum _{({\jmath }, k, \mathbf {\sigma }, - \sigma ) \in A} \!\!\!\!\!\!\! X_{\ j_1, j_2, j_3, k }^{\sigma _1, \sigma _2, \sigma _3, \sigma } \ u_{j_1}^{\sigma _1} \, u_{j_2}^{\sigma _2} \, u_{j_3}^{\sigma _3}\, { e^{\textrm{i}\sigma k x }} \ . \end{aligned}$$

We now compute the projections of the cubic vector field in (3.2), which we denote by

3.13 $$\begin{aligned} X_3(U)^+ := |u|^2 u_x - \mathscr {M}(u)u_x+ \textrm{i}\mathscr {P}(u)u \end{aligned}$$

on the sets $\textsf{R}^{(n)}_\Lambda$ defined in (3.5) for $n=0,1,2$.

### Lemma 3.4

The cubic, translation and gauge invariant vector field $X_3(U)^+$ in (3.13) fulfills

(i) **Structure:** There exists a 2-homogeneous 1-operator $M_\texttt {NLS}^+(U) \in {\widetilde{{{\mathcal {M}}}}}_2^1$ such that $X_3(U)^+ = M_\texttt {NLS}^+(U)u$;
(ii) **Resonances:** The projections of the vector field $X_3(U)^+$ on the sets $\textsf{R}_\Lambda ^{(n)}$, $n=0,1,2$, defined in (3.5) are given by
  3.14 $$\begin{aligned} \begin{aligned}&(\Pi _{\textsf{R}_\Lambda ^{(0)}} X_3)(U)^+ = - \textrm{i}|u_1|^2 u_1 \, e^{\textrm{i}x} + \textrm{i}|u_{-1}|^2 u_{-1} \, e^{-\textrm{i}x} \ , \\&(\Pi _{\textsf{R}_\Lambda ^{(1)}} X_3) (U)^+= 0 \ , \quad (\Pi _{\textsf{R}_\Lambda ^{(2)}} X_3)(U)^+ = 0 \ . \end{aligned} \end{aligned}$$

### Proof

(*i*) Define $M_{\texttt{NLS}}^+(U)$ to be the operator

3.15 $$\begin{aligned} M_{\texttt{NLS}}^+(U)v := \big (|u|^2 - \mathscr {M}(u) \big ) \ \partial _x v + \textrm{i}\mathscr {P}(u) v \ , \end{aligned}$$

so that $M_{\texttt{NLS}}^+(U)u = X_3(U)^+$. To prove that $M_{\texttt{NLS}}^+(U) \in {\widetilde{{{\mathcal {M}}}}}_2^1$ we write it in Fourier as

$$\begin{aligned} \begin{aligned}&M_{\texttt{NLS}}^+(U)v = \sum _{\sigma _1 j_1 +\sigma _2 j_2 +j = k \atop \sigma _1 + \sigma _2 = 0 } M_{j_1, j_2, j, k}^{\sigma _1, \sigma _2} \, u_{j_1}^{\sigma _1} u_{j_2}^{\sigma _2} v_{j} \, {e^{\textrm{i}k x}} , \\&\quad M_{j_1, j_2, j, k}^{\sigma _1, \sigma _2 } := {\left\{ \begin{array}{ll} \frac{\textrm{i}}{2}j &  \text{ if } j_1 \ne j_2 , \ j \ne k , \ \sigma _1 \ne \sigma _2 \\ - \frac{\textrm{i}}{2} j_1 &  \text{ if } j_1 = j_2 , \ j = k \ , \ \sigma _1 \ne \sigma _2 \ . \\ 0 &  \text{ otherwise } \\ \end{array}\right. } \end{aligned} \end{aligned}$$

The coefficients $M_{j_1, j_2, j, k}^{\sigma _1, \sigma _2 }$ are symmetric in the first two indexes and fulfill (2.32) with $m = 1$ and $\mu = 0$.

(*ii*) As we shall compute the projectors using the definition (3.12), we need first to write $X_3(U)^+$ in the form (3.11). So expand $X_3(U)^+$ in (3.13) in Fourier, getting

$$\begin{aligned} X_3(U)^+ =&\sum _{j_1 - j_2 + j_3 = k \atop j_1 \ne j_2 } \textrm{i}j_3 u_{j_1} {\overline{u}}_{j_2} u_{j_3} e^{\textrm{i}k x} - \sum _{ j_1 = j_2, \ j_3=k} \textrm{i}j_2 \, |u_{j_2}|^2 u_{j_3} e^{\textrm{i}k x} \\ =&\sum _{(\mathbf {\jmath },k, \mathbf {\sigma },-)\in \mathcal {P}_4 } N_{\mathbf {\jmath },k}^{\mathbf {\sigma },+} u_{\mathbf {\jmath }}^{\mathbf {\sigma }} e^{\textrm{i}k x}, \end{aligned}$$

where

$$\begin{aligned} N_{j_1,j_2,j_3,k}^{\sigma _1,\sigma _2,\sigma _3,+}:= \textrm{i}( j_3 \delta _{j_1 \ne j_2}- j_2\delta _{j_1=j_2}\delta _{j_3=k})\delta _{(\sigma _1,\sigma _2,\sigma _3)=(+,-,+)} \ . \end{aligned}$$

The coefficients of expansion (3.11) are obtained by the symmetrization

$$\begin{aligned} X_{j_1,j_2,j_3,k}^{\sigma _1,\sigma _2,\sigma _3,+}= \frac{1}{6} \sum _{\pi \in \mathcal {S}_3} N_{j_{\pi (1)},j_{\pi (2)},j_{\pi (3)},k}^{\sigma _{\pi (1)},\sigma _{\pi (2)},\sigma _{\pi (3)},+}, \end{aligned}$$

yielding

3.16 $$\begin{aligned} X_{j_1,j_2,j_3,k}^{+,-,+,+} = \frac{\textrm{i}}{6} \left( j_3 \delta _{j_1 \ne j_2} + j_1 \delta _{j_3 \ne j_2} - j_2 (\delta _{j_1 = j_2} + \delta _{j_3 = j_2} ) \right) . \end{aligned}$$

$\underline{\hbox {Projection on } \textsf{R}_\Lambda ^{(0)}:}$ We use the definition of projections in (3.12). In view of the characterization of $\textsf{R}_\Lambda ^{(0)}$ given in (3.7), we must consider only those monomials with indexes of the form $\Big ( (k, k, \ell , \ell ), (+,-, +, -) \Big )$ with $k, \ell \in \{ \pm 1\}$ and their permutations. Once the last couple $(\ell ,-)$ is fixed, than either $k=\ell$, giving the index $\big ((\ell , \ell , \ell , \ell ), (+,-, +,-) \big )$ and its 3 permutations, or $k=-\ell$, giving $\big ((-\ell , -\ell , \ell , \ell ), (+,-, +,-) \big )$ and its 6 permutations. Therefore we obtain

$$\begin{aligned} ( \Pi _{\textsf{R}_\Lambda ^{(0)}} X_3)(U)^+&= \Big ( 3 X_{\ 1, 1, 1, 1}^{+, -, +, +} \, |u_{1}|^2 u_{1} +6 X_{ -1, -1, 1, 1}^{+, -, +, +} \, |u_{-1}|^2 u_{1} \, \Big ) {e^{\textrm{i}x} } \\&\ \ \ + \Big ( 6 X_{\ 1, 1, -1, -1}^{+, -, +, +} \, |u_{1}|^2 u_{-1} +3 X_{ -1, -1, -1, -1}^{+, -, +, +} \, |u_{-1}|^2 u_{-1} \, \Big ) {e^{-\textrm{i}x} } \\&{\mathop {=}\limits ^{(3.16)}} - \textrm{i}|u_1|^2 u_1 {e^{\textrm{i}x} } + \textrm{i}|u_{-1}|^2 u_{-1} {e^{-\textrm{i}x} }, \end{aligned}$$

proving the first of (3.14).

$\underline{\hbox {Projection on } \textsf{R}_\Lambda ^{(1)}:}$ It is zero since $\textsf{R}_\Lambda ^{(1)} = \emptyset$ by Lemma 3.3 (*ii*).

$\underline{\hbox {Projection on } \textsf{R}_\Lambda ^{(2)}:}$ In view of the characterization of $\textsf{R}_\Lambda ^{(2)}$ in (3.9), the monomials surviving the projection have indexes of the form $\Big ( (k, k, \ell , \ell ), (+,-, +, -) \Big )$ (and their permutations) with only one among *k* and $\ell$ in $\Lambda$. Once the last index $(\ell , -)$ is fixed in either $\Lambda$ or $\Lambda ^c$, and *k* is fixed in the complementary set, there are 6 possible permutations. Hence we get

$$\begin{aligned} ( \Pi _{\textsf{R}_\Lambda ^{(2)}} X_3)(U)^+&= \sum _{k \in \Lambda ^c} 6 X_{k, k, 1, 1}^{+, -, +, +} \, |u_{k}|^2 u_{1} {e^{\textrm{i}x} } + \sum _{k\in \Lambda ^c} 6 X_{k, k, -1, -1}^{+, -, +, +} \, |u_{k}|^2 u_{-1} {e^{-\textrm{i}x} }\\&+ \sum _{\ell \in \Lambda ^c} \sum _{k = \pm 1 } 6 X_{k, k, \ell , \ell }^{+, -, +, +} \, |u_{k}|^2 u_{\ell } \, {e^{\textrm{i}\ell x} } {\mathop {=}\limits ^{(xm{3.16})}} 0 \end{aligned}$$

proving the last of (3.14). $\square$

For later use, we now prove a lemma about the projections on $\textsf{R}^{(n)}_{\Lambda }$, $n=0,1,2$, of cubic paradifferential vector fields. More precisely, we have

### Lemma 3.5

Let $a(Z; x, \xi )$ be a 2-homogeneous symbol in ${\widetilde{\Gamma }}_2^m$, $m \in {{\mathbb {R}}}$, with zero average and fulfilling $a({\mathtt g}_\theta Z; \cdot ) = a(Z; \cdot )$ for any $\theta \in {{\mathbb {T}}}$, where ${\mathtt g}_\theta$ in (2.2). Then

$$\begin{aligned} \Pi _{\textsf{R}^{(n)}_{\Lambda } } \left[ \textrm{Op}^{\scriptscriptstyle BW}_{\texttt {vec}}\!\left( a(Z; x, \xi )\right) Z \right] = 0 \ , \quad n = 0,1,2 \ . \end{aligned}$$

### Proof

Recalling (2.27), $\left( \textrm{Op}^{\scriptscriptstyle BW}_{\texttt {vec}}\!\left( a(Z; x, \xi )\right) Z \right) ^+ = \textrm{Op}^{\!\scriptscriptstyle BW}\!\left( a(Z; x, \xi )\right) z$. Using definition (2.22) specialized to quadratic symbols fulfilling $a({\mathtt g}_\theta Z; \cdot ) = a(Z; \cdot )$, $\forall \theta \in {{\mathbb {T}}}$, and the comments right below (2.26), we get

$$\begin{aligned} \textrm{Op}^{\!\scriptscriptstyle BW}\!\left( a(Z; x, \xi )\right) z = \sum _{ j_1-j_2 +j=k} \chi _2 \left( j_1, j_2,\frac{j+k}{2}\right) a_{j_1, j_2}^{+,-}\left( \frac{j+k}{2}\right) z_{j_1} {\overline{z}}_{j_2} z_j {e^{\textrm{i}k x}} \ . \end{aligned}$$

The point is that, when projecting on $\Pi _{\textsf{R}^{(n)}_{\Lambda } }$, $n = 0,1,2$, either the cut-off $\chi _2(\cdot , \cdot )$ or the coefficient $a_{j_1, j_2}^{+,-}$ vanish. Recall that $\chi _2(\xi ', \xi ) \equiv 0$ whenever $|\xi '| > \frac{\langle \xi \rangle }{10}$.

$\underline{\hbox {Case }n = 0:}$ In this case $j_1, j_2, j, k \in \Lambda$, and $\chi _2 \left( j_1, j_2,\frac{j+k}{2}\right) = 0$ for any choice of $j_1, j_2, j, k$.

$\underline{\hbox {Case }n = 1:}$ By Lemma 3.3$\textsf{R}_\Lambda ^{(1)} = \emptyset$ and there is nothing to prove.

$\underline{\hbox {Case } n = 2:}$ By Lemma 3.3 the indexes $j_1, j_2, j, k$ are pairwise equal.

Assume first that $j_1 = j_2$, then $a_{j_1, j_1}^{+,-} = 0$ since $a(Z;\cdot )$ has zero-average in *x*.

The case $j_1 = j \in \Lambda$ and $j_2 = k \in \Lambda ^c$ violates the momentum conservation, as well as $j_1 = j \in \Lambda ^c$, $j_2=k \in \Lambda$.

In case $j_1 = k \in \Lambda$ and $j_2 = j \in \Lambda ^c$, the cut-off vanishes since

$$\begin{aligned} \chi _2 \left( \pm 1, j,\frac{j +k}{2}\right) \equiv 0 \quad \forall k \in \Lambda , \ j \in \Lambda ^c \ . \end{aligned}$$

Analogously, the case $j_1 = k \in \Lambda ^c$, $j_2 = j \in \Lambda$ is ruled out, concluding the proof. $\square$

**Identification argument.** We prove an abstract identification argument in the spirit of [10, 11]. In section 4 we shall conjugate equation (3.2) with an admissible transformation. Without doing explicit computations, we shall a posteriori identify the explicit form of the resonant parts of the conjugated vector field thanks to the following proposition:

### Proposition 3.6

(Identification of the resonant normal form) Let $\textbf{F}(U)$ be a 2-admissible transformation (see Definition 2.11) with a gain $\varrho \ge 0$. There exist $r,s_0>0$ such that, provided $U(t) \in B_{s_0, {{\mathbb {R}}}}(I;r)$ is a solution of the system

3.17 $$\begin{aligned} \partial _t U= -\textrm{i}{\mathbf {\Omega }}(D)U+ X_3(U), \qquad {\mathbf {\Omega }}(D): = \begin{pmatrix} |D|^\alpha &  0 \\ 0 &  - |D|^\alpha \end{pmatrix} \end{aligned}$$

where

3.18 $$\begin{aligned} X_3(U)=M_2(U)U \ , \quad M_2(U) \text{ a } \text{ matrix } \text{ of } \text{ operators } \text{ in } {\widetilde{{{\mathcal {M}}}}}^1_2 \ , \end{aligned}$$

then the variable $Z:=\mathcal {F}(U) = \textbf{F}(U)U$ solves

3.19 $$\begin{aligned} \partial _t Z = -\textrm{i}{\mathbf {\Omega }}(D)Z + {\widetilde{X}}_3(Z)+ {\widetilde{M}}_{\ge 4}(Z)Z \ . \end{aligned}$$

Here ${\widetilde{M}}_{\ge 4}(Z)$ is a matrix of non-homogeneous 7-operators in ${{\mathcal {M}}}^7_{\ge 4}[r]$, whereas ${\widetilde{X}}_3(Z)$ is a cubic vector field fulfilling

3.20 $$\begin{aligned} \Pi _\textsf{A}{\widetilde{X}}_3= \Pi _\textsf{A}X_3, \quad \text {for any } \textsf{A}\subseteq \textsf{R}\ \end{aligned}$$

where $\textsf{R}$ is the 4-waves resonant set in (3.3).

### Proof

Defining $X(U):= -\textrm{i}{\mathbf {\Omega }}(D)U+ X_3(U)$, the variable *Z* solves the equation

$$\begin{aligned} \partial _t Z= \mathcal {F}^* X (Z):= \textrm{d}_U \mathcal {F}(U)\left[ X(U)\right] _{|U=\mathcal {F}^{-1}(Z)}, \end{aligned}$$

where to invert the nonlinear map ${\mathcal {F}}$ we used Lemma 2.14.

Next we provide a Taylor expansion of the push-forward vector field $\mathcal {F}^*X$. Using the expansion (2.44) for ${{\mathcal {F}}}(U) = \textbf{F}(U)U$, we get

3.21 $$\begin{aligned}  &   \textrm{d}_U \mathcal {F}(U)\left[ X(U)\right] = -\textrm{i}{\mathbf {\Omega }}(D)U+ X_3(U) \\  &   \quad + \textbf{F}_2(U)[-\textrm{i}{\mathbf {\Omega }}(D)U]+\textrm{d}_U \textbf{F}_2(U)[-\textrm{i}{\mathbf {\Omega }}(D)U ]U+ M_{\ge 4}(U)U\nonumber , \end{aligned}$$

where, using the structure (3.18) of $X_3(U)$

3.22 $$\begin{aligned} M_{\ge 4 }(U)W:=&- \textbf{F}_{\ge 4}(U)\textrm{i}{\mathbf {\Omega }}(D)W+\textbf{F}_{\ge 4}(U)M_2(U)W+ \textrm{d}_U \textbf{F}_{\ge 4}(U)[X(U) ]W\nonumber \\&+ \textbf{F}_2(U)M_2(U)W+ \textrm{d}_U \textbf{F}_2(U)[X_3(U)]W. \end{aligned}$$

We prove in Lemma 3.7 below that $M_{\ge 4}(U)$ is a matrix of non-homogeneous operators in $\mathcal {M}_{\ge 4 }^3[r]$. Next we compute (3.21) at

3.23 $$\begin{aligned} U=\mathcal {F}^{-1}(Z){\mathop {=}\limits ^{(2.50)}} \textbf{G}(Z)Z, \qquad \textbf{G}(Z)-\textrm{Id}= \underbrace{- \textbf{F}_2(Z)+ \textbf{G}_{\ge 4}(Z)}_{=:\textbf{G}_{\ge 2}(Z)} \in \Sigma {{\mathcal {M}}}^4_{2}[r],\nonumber \\ \end{aligned}$$

obtaining

$$\begin{aligned} \mathcal {F}^*X(Z)= - \textrm{i}{\mathbf {\Omega }}(D)Z+ {\widetilde{X}}_3(Z)+ {\widetilde{M}}_{\ge 4}(Z)Z \end{aligned}$$

where3.24and

3.25 $$\begin{aligned} {\widetilde{M}}_{\ge 4}(Z)W=&-\textrm{i}{\mathbf {\Omega }}(D)\textbf{G}_{\ge 4}(Z)W+ \big [M_2(\mathcal {F}^{-1}(Z))\textbf{G}(Z)-M_2(Z)\big ]W \nonumber \\&-\big [ \textbf{F}_2(\mathcal {F}^{-1}(Z))\textrm{i}{\mathbf {\Omega }}(D)\textbf{G}(Z)- \textbf{F}_2(Z)\textrm{i}{\mathbf {\Omega }}(D)\big ]W\nonumber \\&-\big [\textrm{d}_U \textbf{F}_2(\mathcal {F}^{-1}(Z))[\textrm{i}{\mathbf {\Omega }}(D)\mathcal {F}^{-1}(Z) ]\textbf{G}(Z)-\textrm{d}_Z \textbf{F}_2(Z)[\textrm{i}{\mathbf {\Omega }}(D)Z ]\big ]W\nonumber \\&+M_{\ge 4}(\mathcal {F}^{-1}(Z))\textbf{G}(Z)W \end{aligned}$$

We prove in Lemma 3.7 below that ${\widetilde{M}}_{\ge 4}(Z)$ belongs to $\mathcal {M}_{\ge 4 }^7[r]$. This concludes the proof of (3.19). To prove (3.20) we note thatit then follows that, for any set $\textsf{A}\subseteq \textsf{R}$, one haswhich, together with (3.24), implies (3.20). $\square$

### Lemma 3.7

There is $r>0$ such that $M_{\ge 4}(U)$ defined in (3.22) is a matrix of 3-operators in $\mathcal {M}^3_{\ge 4}[r]$ and ${\widetilde{M}}_{\ge 4}(Z)$ defined in (3.25) is a matrix of 7-operators in $\mathcal {M}^7_{\ge 4}[r]$.

### Proof

We need to show that each term in (3.22) and (3.25) fulfills (2.33) with $$p=4$$, some $$ s_0 \ge 0$$ and *m* equal 3 or 7. This is proved exploiting that each term is a composition of either *m*-operators or differentials of admissible transformations and therefore satisfying (2.46). As an example, we explicitly show how to bound the most difficult terms in (3.22) and (3.25). Recall that, by definition of admissible transformations, $$\textbf{F}(U) - \textrm{Id}$$ is a matrix of 2-operators in $$\Sigma {{\mathcal {M}}}_2^{2}[r]$$ for some $$ r >0$$.

We start from $\textrm{d}_U \textbf{F}_{\ge 4}(U)[X(U)]W$ in (3.22). Using (2.46) (with $s\leadsto s-1$ and $m =2$) and that $\Vert X(U)\Vert _{s-1}\lesssim \Vert U\Vert _s$, we get

$$\begin{aligned} \Vert \textrm{d}_U \textbf{F}_{\ge 4}(U)[X(U) ]W\Vert _{s-3}\lesssim&\Vert U\Vert _{s_0}^3\Vert X(U)\Vert _{s_0}\Vert W\Vert _{s-1}+ \Vert U\Vert _{s_0}^3\Vert X(U)\Vert _{s-1}\Vert W\Vert _{s_0}\\&+\Vert U\Vert _{s_0}^2 \Vert U\Vert _{s-1}\Vert X(U)\Vert _{s_0}\Vert W\Vert _{s_0}\\ \lesssim&\Vert U\Vert _{s_0+1}^4\Vert W\Vert _{s}+ \Vert U\Vert _{s_0+1}^3\Vert U\Vert _{s}\Vert W\Vert _{s_0+1}, \end{aligned}$$

proving (2.33) with $$s_0\leadsto s_0+1$$.

Now we consider the term in the third line of (3.25). Using the trilinearity of $(V, V', W) \mapsto \textrm{d}_U \textbf{F}_2(V)[ V']W$ and (3.23), we decompose that as

3.26 $$\begin{aligned}&\big [\textrm{d}_U \textbf{F}_2(\mathcal {F}^{-1}(Z))[\textrm{i}{\mathbf {\Omega }}(D)\mathcal {F}^{-1}(Z) ]\textbf{G}(Z)-\textrm{d}_Z \textbf{F}_2(Z)[\textrm{i}{\mathbf {\Omega }}(D)Z ]\big ]W\nonumber \\&= \textrm{d}_U \textbf{F}_2\left( \textbf{G}_{\ge 2}(Z)Z\right) [ \textrm{i}{\mathbf {\Omega }}(D)\mathcal {F}^{-1}(Z)] \textbf{G}(Z)W +\textrm{d}_U \textbf{F}_2(Z)[\textrm{i}{\mathbf {\Omega }}(D)\mathcal {F}^{-1}(Z) ]\textbf{G}_{\ge 2}(Z)W \nonumber \\&\quad +\textrm{d}_U \textbf{F}_2(Z)[\textrm{i}{\mathbf {\Omega }}(D)\textbf{G}_{\ge 2}(Z) ]W. \end{aligned}$$

We bound each term in (3.26) separately. We shall repeatedly use that $\Vert {\mathbf {\Omega }}(D)U\Vert _{s-\alpha }\le \Vert U\Vert _s$. First, using (2.47) and then (2.36), (2.33), (2.55) and (3.23), we get

3.27 $$\begin{aligned} \Vert \textrm{d}_U \textbf{F}_2&\left( \textbf{G}_{\ge 2}(Z)Z\right) [ \textrm{i}{\mathbf {\Omega }}(D)\mathcal {F}^{-1}(Z)] \textbf{G}(Z)W\Vert _{s-7}\nonumber \\ \lesssim&\Vert \textbf{G}_{\ge 2}(Z)Z\Vert _{s_0}\Vert {\mathbf {\Omega }}(D)\mathcal {F}^{-1}(Z)\Vert _{s_0}\Vert \textbf{G}(Z)W\Vert _{s-5}\nonumber \\&+\Vert \textbf{G}_{\ge 2}(Z)Z\Vert _{s_0}\Vert {\mathbf {\Omega }}(D)\mathcal {F}^{-1}(Z)\Vert _{s-5}\Vert \textbf{G}(Z)W\Vert _{s_0}\nonumber \\&+\Vert \textbf{G}_{\ge 2}(Z)Z\Vert _{s-5}\Vert {\mathbf {\Omega }}(D)\mathcal {F}^{-1}(Z) \Vert _{s_0}\Vert \textbf{G}(Z)W \Vert _{s_0}\nonumber \\ \lesssim&\Vert Z\Vert _{s_0+4}^4\Vert W\Vert _{s}+\Vert Z\Vert _{s_0+4}^3\Vert Z\Vert _{s}\Vert W\Vert _{s_0+4}. \end{aligned}$$

Similarly one obtains

3.28 $$\begin{aligned}  &   \Vert \textrm{d}_U \textbf{F}_2\left( Z\right) [ \textrm{i}{\mathbf {\Omega }}(D)\mathcal {F}^{-1}(Z)] \textbf{G}_{\ge 2}(Z)W\Vert _{s-7} \nonumber \\    &   \quad \lesssim \Vert Z\Vert _{s_0+4}^4\Vert W\Vert _{s}+\Vert Z\Vert _{s_0+4}^3\Vert Z\Vert _{s}\Vert W\Vert _{s_0+4}. \end{aligned}$$

Finally, using (2.47) and then (2.36), (2.33) and (3.23), we get

3.29 $$\begin{aligned}&\Vert \textrm{d}_U \textbf{F}_2\left( Z\right) [ \textrm{i}{\mathbf {\Omega }}(D)\textbf{G}_{\ge 2}(Z)Z] W\Vert _{s-7}\nonumber \\&\lesssim \Vert Z\Vert _{s_0}\Vert {\mathbf {\Omega }}(D)\textbf{G}_{\ge 2}(Z)Z\Vert _{s_0}\Vert W\Vert _{s-5}+\Vert Z\Vert _{s_0}\Vert {\mathbf {\Omega }}(D)\textbf{G}_{\ge 2}(Z)Z\Vert _{s-5}\Vert W\Vert _{s_0}\nonumber \\&\quad +\Vert Z\Vert _{s-5}\Vert {\mathbf {\Omega }}(D)\textbf{G}_{\ge 2}(Z)Z \Vert _{s_0}\Vert W \Vert _{s_0}\nonumber \\&\lesssim \Vert Z\Vert _{s_0+5}^4\Vert W\Vert _{s}+\Vert Z\Vert _{s_0+5}^3\Vert Z\Vert _{s}\Vert W\Vert _{s_0+5}. \end{aligned}$$

Estimates (3.27), (3.28) and (3.29) prove that the operator in (3.26) is a non-homogeneous 7-operator in $\mathcal {M}_{\ge 4}^{7}[r]$. $\square$

## Paradifferential Normal Form

The goal of this section is to use paradifferential transformations and Birkhoff normal forms, in the spirit of [9], to put the quasilinear equation (3.2) into a suitable normal form. However, the normal form that we shall obtain is rather different from the one of [9] and of [10, 11, 13, 63]; indeed, in these papers, the paradifferential part has symbols with constant coefficients (at least at low homogeneity), and the smoothing vector field is in Birkhoff normal form, namely supported only on resonant monomials. On the contrary, our normal form has to two important and different features, see Theorem 4.4: (*i*) the cubic part of the paradifferential vector field has a dominant transport term with *variable coefficients* and supported only on resonant sites, see (4.8), and (*ii*) the cubic smoothing vector field is in a suitable weak normal form, that we call $\Lambda$-normal form and we now introduce.

### Definition 4.1

($\Lambda$-normal form) Let $\Lambda = \{1,-1\}$ as in (3.6). A cubic, translation and gauge invariant vector field *X*(*U*) is said to be in

- *weak*-$\Lambda$ *normal form* if all its monomials with at most two indexes outside $\Lambda$ are resonant, i.e.
  $$\begin{aligned} \begin{aligned}&\Pi _{{\mathcal {P}}^{(n)}_\Lambda } X = \Pi _{\textsf{R}^{(n)}_\Lambda } X \ , \quad n = 0,1,2 \ ; \end{aligned} \end{aligned}$$
- *strong*-$\Lambda$ *normal form* if in addition there are no resonant monomials with one or two indexes outside $\Lambda$, i.e.
  $$\begin{aligned} \begin{aligned}&\Pi _{{\mathcal {P}}^{(0)}_\Lambda } X = \Pi _{\textsf{R}^{(0)}_\Lambda } X \ , \quad \Pi _{{\mathcal {P}}^{(1)}_\Lambda } X = \Pi _{{\mathcal {P}}^{(2)}_\Lambda } X = 0 \ , \end{aligned} \end{aligned}$$

the sets ${{\mathcal {P}}}^{(n)}_\Lambda$, $\textsf{R}^{(n)}_\Lambda$ being defined in (3.4) and (3.5).

Note that a cubic vector field in *strong*-$\Lambda$ *normal form* is composed by monomials $u_{j_1}^{\sigma _1} u_{j_2}^{\sigma _2} u_{j_3}^{\sigma _3} e^{\textrm{i}\sigma k x}$ whose indexes $\big ( (j_1, j_2, j_3, k), (\sigma _1, \sigma _2, \sigma _3, -\sigma ) \big )$ are

- either in $\Lambda$ and resonant, i.e. $\big ( (j_1, j_2, j_3, k), (\sigma _1, \sigma _2, \sigma _3, -\sigma ) \big ) \in \textsf{R}^{(0)}_\Lambda$;
- or at least three indexes are outside $\Lambda$, i.e. $\big ( (j_1, j_2, j_3, k), (\sigma _1, \sigma _2, \sigma _3, -\sigma ) \big ) \in {\mathcal {P}}^{(3)}_\Lambda \cup {\mathcal {P}}^{(4)}_\Lambda$.

To start the normal form procedure, it is convenient to write (3.2) as the system in the variable $$U:= \Big ({\begin{smallmatrix}u \\ {\overline{u}}\end{smallmatrix}}\Big )$$ given by

4.1 $$\begin{aligned} \partial _t U = - \textrm{i}{\mathbf {\Omega }}(D) U + X_3(U) , \quad X_3(U) = \begin{pmatrix}|u|^2 u_x - \mathscr {M}(u) u_x + \textrm{i}\mathscr {P}(u) u \\ |u|^2\overline{u_x} - \mathscr {M}(u)\overline{u_x} - \textrm{i}\mathscr {P}(u) {\overline{u}}\end{pmatrix}\qquad \end{aligned}$$

where ${\mathbf {\Omega }}(D)$ is defined in (3.17) and, with $M_{\texttt{NLS}}^+$ the 1-operator in ${\widetilde{{{\mathcal {M}}}}}_2^1$ in (3.15),

4.2 $$\begin{aligned} X_3(U) =M_{\texttt{NLS}}(U)U \ , \qquad M_{\texttt{NLS}}(U) := \begin{pmatrix} M_{\texttt{NLS}}^+(U) &  0 \\ 0 &  \overline{M_{\texttt{NLS}}^+(U) } \end{pmatrix} \ . \end{aligned}$$

The first step is to paralinearize such system.

### Lemma 4.2

(Paralinearization) Fix $\varrho \ge 0$ and $s_0 > \varrho +\frac{3}{2}$. If $u(t) \in H^{s_0}({{\mathbb {T}}}, {{\mathbb {C}}})$ solves equation (3.2), then $$U(t) = \Big ({\begin{smallmatrix}u(t) \\ {\overline{u}}(t)\end{smallmatrix}}\Big )$$ solves the system in paradifferential form (recall the notation in (2.27))

4.3 $$\begin{aligned} \partial _t U= &   - \textrm{i}{\mathbf {\Omega }}(D) U + \textrm{Op}^{\scriptscriptstyle BW}_{\texttt {vec}}\!\left( \textrm{i}\, \underline{{\mathtt V}}(U;x) \xi +\textrm{i}\, \underline{{\mathtt d}}(U;x) \right) U \nonumber \\    &   + \textrm{Op}^{\scriptscriptstyle BW}_{\texttt {out}}\!\left( b(U;x)\right) U + R_2(U) U \end{aligned}$$

where:

$\bullet$ ${\mathbf {\Omega }}(D)$ is the matrix of Fourier multipliers in (3.17);

$\bullet$ ${\mathtt V}(U;x),\, \underline{{\mathtt d}}(U;x), \in {\widetilde{{{\mathcal {F}}}}}^{{\mathbb {R}}}_2$ and $b(U;x) \in {\widetilde{{{\mathcal {F}}}}}_2$ are the zero-average, 2-homogeneous functions

4.4 $$\begin{aligned}&\underline{{\mathtt V}}(U;x) := |u|^2 - \mathscr {M}(u) = \sum _{k_1\not =k_2} u_{k_1}{\overline{u}}_{k_2} \, e^{ \textrm{i}(k_1-k_2) x }, \nonumber \\&\underline{{\mathtt d}}(U;x):= \,Im (u_x {\overline{u}}) - \mathscr {P}(u) = \textrm{Im }\sum _{k_1\not = k_2 } \textrm{i}\, k_1 u_{k_1} {\overline{u}}_{k_2} e^{ \textrm{i}(k_1-k_2) x } \ , \nonumber \\&b(U;x):= u u_x = \sum _{k_1,k_2\in {{\mathbb {Z}}}} \textrm{i}\frac{k_1+k_2}{2} u_{k_1}u_{k_2}e^{\textrm{i}(k_1+k_2)x}, \end{aligned}$$

where $\mathscr {M}(u),\, \mathscr {P}(u)$ are the mass and momentum defined in (3.1);

$\bullet$ $R_2(U)$ is a real-to-real, gauge invariant matrix of smoothing operators in ${\widetilde{{{\mathcal {R}}}}}^{-\varrho }_{2}$.

### Proof

The nonlinearity $|u|^2 u_x$ is paralinearized in a standard way using Lemma 2.9 and Proposition  2.8, getting a smoothing remainder *R*(*U*) whose coefficients fulfill (2.32) with $\mu \leadsto \varrho +1$ and $m\leadsto - \varrho$. Note also that, in view of the Bony quantization (2.21), (2.22) for homogeneous symbols

$$\begin{aligned} \mathscr {M}(u) u_x = \textrm{Op}^{\!\scriptscriptstyle BW}\!\left( \mathscr {M}(u) \textrm{i}\xi \right) u + R(U)u \ , \quad \mathscr {P}(u) u = \textrm{Op}^{\!\scriptscriptstyle BW}\!\left( \mathscr {P}(u)\right) u + R(U)u \end{aligned}$$

for some smoothing remainders in ${\widetilde{{{\mathcal {R}}}}}^{-\varrho }_{2}$. Finally, remark that equation (4.1) is real-to-real and gauge invariant. Since also the paradifferential operators in (4.3) are real-to-real and gauge invariant (see (2.25) and (2.26)), by difference so is the matrix of smoothing operators $R_2(U)$. $\square$

### Remark 4.3

Exploiting the continuity Theorem 2.4 and the symbolic calculus of Proposition 2.8, one checks easily that a solution of (4.3) (namely the paralinearization of (3.2)) fulfills the cubic energy estimate

4.5 $$\begin{aligned} \partial _t \Vert U(t) \Vert _s^2 \lesssim \Vert U(t) \Vert _{s_0}^2 \Vert U(t) \Vert _s^2 \end{aligned}$$

for any $s> s_0 > \frac{3}{2}$. It is then standard to deduce local well-posedness in $H^s$, $s > \frac{3}{2}$, for equation (4.3) —see e.g. the scheme in [62, Chapter 7]. Moreover, the energy estimate (4.5) shows that initial data of size $0<\delta \ll 1$ gives rise to solution remaining of size $\sim 2 \delta$ for times of order $\delta ^{-2}$.

The main result of the section is the following normal form theorem:

### Theorem 4.4

There exist $s_0, r >0$ and a 2-admissible transformation $\textbf{F}(U) \in {{\mathcal {M}}}_{\ge 0}^0[r]$ with gain 3 (see Definition 2.11) such that if $U(t) \in B_{s_0, {{\mathbb {R}}}}(I;r)$ solves (4.3), then the variable

4.6 $$\begin{aligned}  &   Z:= \mathcal {F}(U):=\textbf{F}(U)U \quad \text{ solves } \end{aligned}$$

4.7 $$\begin{aligned}  &   \begin{aligned} \partial _t Z=&- \textrm{i}{\mathbf {\Omega }}(D)Z + \textrm{Op}^{\scriptscriptstyle BW}_{\texttt {vec}}\!\left( \textrm{i}\langle \,\underline{{\mathtt V}}\, \rangle (Z;x) \xi + \textrm{i}{ a}^{(\alpha ) }_2(Z;x,\xi )\right) Z + {R}_2^{(\Lambda )}(Z)Z \\&+ \textrm{Op}^{\scriptscriptstyle BW}_{\texttt {vec}}\!\left( \textrm{i}{\widetilde{V}}_{\ge 4 }(Z;x) \xi + \textrm{i}{\widetilde{a}}^{(\alpha ) }_{\ge 4} (Z;x,\xi )\right) Z + {\widetilde{B}}_{\ge 4}(Z) Z \end{aligned} \end{aligned}$$

where

$\bullet$ ${\mathbf {\Omega }}(D)$ is the matrix of Fourier multipliers in (3.17);

$\bullet$ $\langle \,\underline{{\mathtt V}}\, \rangle (Z;x)$ is the zero-average, real valued function in ${\widetilde{{{\mathcal {F}}}}}^{{\mathbb {R}}}_2$ defined by

4.8 $$\begin{aligned} \langle \,\underline{{\mathtt V}}\, \rangle (Z;x) := 2\, \textrm{Re }\Big (\sum _{n \in {{\mathbb {N}}}} z_{n} \, \overline{z_{-n}} \, e^{\textrm{i}2n x} \Big ) \ ; \end{aligned}$$

$\bullet$ ${ a}_2^{(\alpha )}(Z;x,\xi )$ is a zero average, gauge-invariant, real symbol in ${\widetilde{\Gamma }}_2^{\alpha }$;

$\bullet$ ${\widetilde{V}}_{\ge 4 }(Z;x)$ is a real function in $\mathcal {F}_{\ge 4}^{{{\mathbb {R}}}}[r]$ and ${\widetilde{a}}^{(\alpha ) }_{\ge 4} (Z;x,\xi )$ a real non-homogeneous symbol in $\Gamma ^\alpha _{\ge 4}[r]$;

$\bullet$ ${R}_2^{(\Lambda )}(Z)$ is a real-to-real and gauge invariant matrix of smoothing operators in ${\widetilde{{{\mathcal {R}}}}}_2^{-4}$ such that the cubic vector field

4.9 $$\begin{aligned} X^{(\Lambda )}(Z):= {R}_2^{(\Lambda )}(Z)Z \end{aligned}$$

is in strong-$\Lambda$ normal form (see Definition 4.1). Precisely, with the notation in (3.12),

4.10 $$\begin{aligned} \begin{aligned}&(\Pi _{{\mathcal {P}}^{(0)}_\Lambda } X^{(\Lambda )}) (Z) = \begin{pmatrix} - \textrm{i}|z_1|^2 z_1 \, e^{\textrm{i}x} + \textrm{i}|z_{-1}|^2 z_{-1} \, e^{-\textrm{i}x} \\ \textrm{i}|z_1|^2 \overline{z_1} \, e^{-\textrm{i}x} - \textrm{i}|z_{-1}|^2 \overline{z_{-1}} \, e^{\textrm{i}x} \end{pmatrix} \ , \\&\Pi _{{\mathcal {P}}_\Lambda ^{(1)}} X^{(\Lambda )}= \Pi _{{\mathcal {P}}_\Lambda ^{(2)}} X^{(\Lambda )} = 0 \ . \end{aligned} \end{aligned}$$

$\bullet$ Finally ${\widetilde{B}}_{\ge 4}(Z)$ is a real-to-real matrix of 0-operators in $\mathcal {M}^0_{\ge 4}[r]$.

The rest of the section is devoted to the proof of Theorem 4.4.

### Block diagonalization

The goal of this section is to remove the out-diagonal term $\textrm{Op}^{\scriptscriptstyle BW}_{\texttt {out}}\!\left( b(U;x)\right)$ from equation (4.3) up to quadratic smoothing operators and quartic bounded operators. Precisely we prove:

#### Proposition 4.5

(Block-diagonalization) Let $\varrho \ge 1-\alpha$. There exist $s_0, r >0$ and a 0-admissible transformation $\Psi (U) \in {{\mathcal {M}}}_{\ge 0}^0[r]$ with gain 5 (see Definition 2.11) such that if $U(t) \in B_{ s_0, {{\mathbb {R}}}}(I;r)$ solves (4.3), then the variable4.114.12where

$\bullet$ ${\mathbf {\Omega }}(D)$ is the matrix of Fourier multipliers defined in (3.17);

$\bullet$ $\underline{{\mathtt V}}({U};x)$ and $\underline{{\mathtt d}}({U};x)$ are the zero average functions defined in (4.4);

$\bullet$ $R_2({U})$ is a real-to-real, gauge invariant matrix of homogeneous smoothing remainders in ${\widetilde{{{\mathcal {R}}}}}^{-\varrho }_2$;

$\bullet$ $B_{\ge 4}({U})$ is a real-to-real matrix of non-homogeneous bounded operators in ${{\mathcal {M}}}_{\ge 4}^0[r]$.

#### Proof

We define the map $\Psi (U)$ as the time-1 flow $\Psi (U):= \Psi ^\tau (U)_{|\tau =1}$ of the paradifferential equation

$$\begin{aligned} {\left\{ \begin{array}{ll} \partial _\tau \Psi ^\tau (U)= G(U)\Psi ^\tau (U)\\ \Psi ^0(U)=\textrm{Id}, \end{array}\right. }\quad \text {where} \quad G(U):= \textrm{Op}^{\scriptscriptstyle BW}_{\texttt {out}}\!\left( g_2(U;x,\xi )\right) \end{aligned}$$

and with the 2-homogeneous symbol $$ g_2$$ of the form

4.13 $$\begin{aligned} g_2(U;x,\xi )=\sum _{j_1,j_2\in {{\mathbb {Z}}}} g_{j_1,j_2}(\xi )u_{j_1}u_{j_2}e^{\textrm{i}( j_1+ j_2)x} \in {\widetilde{\Gamma }}_2^{-\alpha } \end{aligned}$$

to be determined. By Lemma 2.16, $\Psi (U)$ is a 0-admissible transformation with arbitrary gain, which, to be concrete, we fix to 5. Moreover, *G* is gauge invariant (see the bullet of formula (2.26)), so is $\Psi ^\tau$ (Remark 2.15). The variable $W=\Psi (U)U$ solves

4.14 $$\begin{aligned} \partial _t W=&\Psi (U) \textrm{Op}^{\scriptscriptstyle BW}_{\texttt {vec}}\!\left( - \textrm{i}|\xi |^\alpha +\textrm{i}\underline{{\mathtt V}}(U;x) \xi +\textrm{i}\underline{{\mathtt d}}(U;x) \right) \Psi (U)^{-1} W \end{aligned}$$

4.15 $$\begin{aligned}&+ \Psi (U)\left[ \textrm{Op}^{\scriptscriptstyle BW}_{\texttt {out}}\!\left( b(U;x)\right) +R_2(U)\right] \Psi (U)^{-1} W \end{aligned}$$

4.16 $$\begin{aligned}&+ \left( \partial _t \Psi (U)\right) \Psi (U)^{-1} W \ . \end{aligned}$$

We first expand (4.14). The Lie expansion formula (see e.g. Lemma A.1 of [11]) says that for any operator *A*(*U*), setting $\texttt{Ad}_{B}[A]:= [B,A]$, one has

4.17 $$\begin{aligned} \Psi (U)A(U)\Psi (U)^{-1}&= A(U)+ \left[ G(U),A(U)\right] \nonumber \\  &\quad + \int _0^1 (1-\tau ) \Psi ^\tau (U) \, \texttt{Ad}^2_{G(U)}[A(U)] \, (\Psi ^\tau (U))^{-1} \, \textrm{d}\tau \ . \end{aligned}$$

Applying this formula with $A=\textrm{Op}^{\scriptscriptstyle BW}_{\texttt {vec}}\!\left( - \textrm{i}|\xi |^\alpha +\textrm{i}\underline{{\mathtt V}}\xi +\textrm{i}\underline{{\mathtt d}}\right)$, using formulas (2.42) we get

$$\begin{aligned} (4.14)&=\textrm{Op}^{\scriptscriptstyle BW}_{\texttt {vec}}\!\left( - \textrm{i}|\xi |^\alpha +\textrm{i}\underline{{\mathtt V}}\xi +\textrm{i}\underline{{\mathtt d}}\right) W \\&\quad + \, \textrm{Op}^{\scriptscriptstyle BW}_{\texttt {out}}\!\left( \textrm{i}(g_2\#_\varrho |\xi |^\alpha + |\xi |^\alpha \#_\varrho g_2) \right) W + R_2'(U)W + B_{\ge 4}(U)W \end{aligned}$$

where $R_2'$ is a matrix of smoothing remainders in ${\widetilde{{{\mathcal {R}}}}}^{-\varrho }_2$ (coming from the first of (2.42)), and the operator $B_{\ge 4}$ is given by

4.18 $$\begin{aligned} B_{\ge 4}(U):= &   \textrm{Op}^{\scriptscriptstyle BW}_{\texttt {out}}\!\left( \textrm{i}\big (g_2 \#_\varrho \underline{{\mathtt V}}\xi - \underline{{\mathtt V}}\xi \#_\varrho g_2\big ) - \textrm{i}\big (g_2 \#_\varrho \underline{{\mathtt d}}+ \underline{{\mathtt d}}\#_\varrho g_2 \big )\right) + R'(U) \nonumber \\  &   + \int _0^1 (1-\tau ) \Psi ^\tau (U) \, \texttt{Ad}^2_{G(U)}[\textrm{Op}^{\scriptscriptstyle BW}_{\texttt {vec}}\!\left( - \textrm{i}|\xi |^\alpha +\textrm{i}\underline{{\mathtt V}}\xi +\textrm{i}\underline{{\mathtt d}}\right) ] \, (\Psi ^\tau (U))^{-1} \, \textrm{d}\tau , \qquad \end{aligned}$$

where $R'$ is a matrix of smoothing operators in ${{\mathcal {R}}}^{-\varrho + (1-\alpha )}_{\ge 4}[r]$. We claim that $B_{\ge 4}$ is a non-homogeneous bounded operator in ${{\mathcal {M}}}^0_{\ge 4}[r]$. Indeed, since $g_2 \in {\widetilde{\Gamma }}^{-\alpha }_2$, $\underline{{\mathtt V}}$ and $\underline{{\mathtt d}}$ belong to ${\widetilde{{{\mathcal {F}}}}}^{{\mathbb {R}}}_2$, and $-\varrho +1-\alpha \le 0$, we get that both the first line of (4.18) and $\texttt{Ad}^2_{G(U)}[\textrm{Op}^{\scriptscriptstyle BW}_{\texttt {vec}}\!\left( - \textrm{i}|\xi |^\alpha +\textrm{i}\underline{{\mathtt V}}\xi +\textrm{i}\underline{{\mathtt d}}\right) ]$ are matrices of 0-operators in ${\widetilde{{{\mathcal {M}}}}}^0_{4}$ and so in ${{\mathcal {M}}}^0_{\ge 4}[r]$ (use the symbolic calculus of Proposition 2.8 and the bullets after Definition 2.5). Finally, being $\Psi ^\tau$ an admissible transformation, also the second line of (4.18) is a matrix of non-homogeneous 0-operators in ${{\mathcal {M}}}^0_{\ge 4}[r]$ (see Remark 2.12—(2)).

Consider now (4.15). Expanding as in (4.17) one see that the 2-homogeneous component remains the unchanged, getting

$$\begin{aligned} \begin{aligned} (4.15) = \textrm{Op}^{\scriptscriptstyle BW}_{\texttt {out}}\!\left( b(U;x)\right) W+ R_2(U)W+ B_{\ge 4}(U)W \end{aligned} \end{aligned}$$

where $B_{\ge 4}(U)$ is another matrix of non-homogeneous 0-operators in ${{\mathcal {M}}}^0_{\ge 4}[r]$.

Finally we consider line (4.16). This time we use the Lie expansion (Lemma A.1 of [11])

$$\begin{aligned} \big (\partial _t \Psi (U)\big ) \Psi (U)^{-1}= \partial _t G(U) +\int _{0}^{1} (1- \tau ) {\Psi }^\tau (U)\, \texttt{Ad}_{G(U)} \,[ \partial _{t}G(U)]({\Psi }^\tau (U))^{-1} \textrm{d}\tau . \end{aligned}$$

Then, using that $g_2(U) \equiv g_2(U,U)$ is a symmetric function of *U*, we get that $\partial _t G(U)= \textrm{Op}^{\scriptscriptstyle BW}_{\texttt {out}}\!\left( \partial _t g_2(U;x,\xi )\right) = 2\textrm{Op}^{\scriptscriptstyle BW}_{\texttt {out}}\!\left( g_2( \partial _t U, U;x,\xi )\right)$. Since *U* solves equation (4.1), we get

$$\begin{aligned} \left( \partial _t \Psi (U) \right) \Psi ^{-1}(U)&=\textrm{Op}^{\scriptscriptstyle BW}_{\texttt {out}}\!\left( 2 g_2(-\textrm{i}{\mathbf {\Omega }}(D)U,U;x,\xi ))\right) + B_{\ge 4}(U) \end{aligned}$$

where, using also (4.2),

$$\begin{aligned}&B_{\ge 4}(U):= \text {Op}^{\scriptscriptstyle BW}_{\texttt {out}}\!\left( 2 g_2(M_{\texttt {NLS}}(U)U,U;x,\xi )\right) \\&\ \ +\int _{0}^{1} (1- \tau ) {\Psi }^\tau (U)\, \texttt {Ad}_{G(U)} \, [ 2\text {Op}^{\scriptscriptstyle BW}_{\texttt {out}}\!\left( g_2( -\text {i}{\mathbf {\Omega }}(D)U \right. \\&\ \ \left. +M_{\texttt {NLS}}(U)U , U;x,\xi )\right) ]({\Psi }^\tau (U))^{-1} \text {d}\tau \, \end{aligned}$$

By Lemma 2.10, the fact that $\Psi ^\tau$ is an admissible transformation, and the bullets after Definition 2.5, we deduce that $B_{\ge 4}$ is a matrix of $(-\alpha )$-operators in ${{\mathcal {M}}}^{-\alpha }_{\ge 4}[r]$.

In conclusion, we get that

4.19 $$\begin{aligned} \partial _t W&= \textrm{Op}^{\scriptscriptstyle BW}_{\texttt {vec}}\!\left( - \textrm{i}|\xi |^\alpha +\textrm{i}\underline{{\mathtt V}}(U) \xi +\textrm{i}\underline{{\mathtt d}}(U) \right) W\nonumber \\&\quad + \textrm{Op}^{\scriptscriptstyle BW}_{\texttt {out}}\!\left( \textrm{i}\big [(g_2(U)\#_\varrho |\xi |^\alpha + |\xi |^\alpha \#_\varrho g_2(U) - 2 g_2( {\mathbf {\Omega }}(D)U,U))\big ] + b(U)\right) W\nonumber \\&\quad + (R_2(U) + R_2'(U))W+ B_{\ge 4}(U)W \end{aligned}$$

where $B_{\ge 4}(U)$ is a matrix of 0-operators in ${{\mathcal {M}}}^{0}_{\ge 4}[r]$. Then the thesis follows from the following lemma.

#### Lemma 4.6

(The out-diagonal homological equation) Let $\varrho > 0$. There exists a symbol $g_2(U;x,\xi )\in {\tilde{\Gamma }}_2^{-\alpha }$ of the form (4.13) such that

4.20 $$\begin{aligned} r_2(U; \cdot ):= \textrm{i}\big [(g_2(U)\#_\varrho |\xi |^\alpha + |\xi |^\alpha \#_\varrho g_2(U) -2 g_2( {\mathbf {\Omega }}(D)U,U))\big ]+ b(U)\in {\widetilde{\Gamma }}_2^{-\varrho }\nonumber \\ \end{aligned}$$

and $r_2(U; \cdot )$ fulfills the second of (2.26).

#### Proof

Thanks to symbolic calculus formula (2.38) (see also (2.39)), we have that for any $g\in {\widetilde{\Gamma }}_2^m$, $m\in {{\mathbb {R}}}$,

$$\begin{aligned} {\left\{ \begin{array}{ll} \texttt{r}[ g](U) := g(U)\#_\varrho |\xi |^\alpha + |\xi |^\alpha \#_\varrho g(U)- 2 g(U)|\xi |^\alpha \in {\widetilde{\Gamma }}_2^{m+\alpha - 2}\\ \texttt{f}[g](U):= 2 g( {\mathbf {\Omega }}(D)U,U) \in {\widetilde{\Gamma }}_2^{m} \end{array}\right. } \end{aligned}$$

Moreover if *g* fulfills the second of (2.26), so do $\texttt{r}[ g]$ and $\texttt{f}[ g]$. Then the homological equation in (4.20) reads

$$\begin{aligned} r_2(U)= 2 \textrm{i}g_2(U) |\xi |^\alpha +\textrm{i}\texttt{r} [ g_2](U)- \textrm{i}\texttt{f}[g_2](U)+b(U)\in {\widetilde{\Gamma }}_2^{-\varrho } \ , \end{aligned}$$

which we solve iteratively exploiting that $g \mapsto {\mathtt r}[g]$ and $g \mapsto {\mathtt f}[g]$ are linear. Namely we put $g_2:= g^{(1)}+g^{(2)}+\dots + g^{(p)}$ with

$$\begin{aligned}&g^{(1)}(U;x,\xi ):= -\frac{b(U;x)}{2 \textrm{i}|\xi |^\alpha }\in {\widetilde{\Gamma }}_2^{-\alpha },\\&g^{(2)}(U;x,\xi ):= - \frac{\textrm{i}\texttt{r}[g^{(1)}](U;x,\xi )- \textrm{i}\texttt{f}[g^{(1)}](U;x,\xi )}{2\textrm{i}|\xi |^\alpha }\in {\widetilde{\Gamma }}_2^{-2\alpha }\\&\vdots \\&g^{(p)}(U;x,\xi ):= - \frac{\textrm{i}\texttt{r}[g^{(p-1)}](U;x,\xi )- \textrm{i}\texttt{f}[g^{(p-1)}](U;x,\xi )}{2\textrm{i}|\xi |^\alpha }\in {\widetilde{\Gamma }}_2^{-p\alpha }. \end{aligned}$$

With this choice we have $r_2(U) = \textrm{i}\texttt{r}[g^{(p)} ](U)- \textrm{i}\texttt{f}[g^{(p)}](U)\in {\widetilde{\Gamma }}_2^{-p\alpha }$ which implies the thesis choosing $p>{\varrho }/{\alpha }$. Moreover, since *b* fulfills the second of (2.26) (recall (4.4)), so does $g^{(1)}$, and by construction each $g^{(\ell )}$, $\ell \ge 2$ and the symbol $r_2(U)$. In particular $g_2$ has the claimed form in (4.13). $\square$

Applying Lemma 4.6, equation (4.19) becomes

4.21 $$\begin{aligned} \partial _t W=&\text {Op}^{\scriptscriptstyle BW}_{\texttt {vec}}\!\left( - \text {i}|\xi |^\alpha +\text {i}\underline{{\mathtt V}}(U) \xi +\text {i}\underline{{\mathtt d}}(U) \right) W \nonumber \\  &+ (R_2(U) + R_2'(U) + R_2''(U))W+ B_{\ge 4}(U)W \end{aligned}$$

where $R_2''(U) = \textrm{Op}^{\scriptscriptstyle BW}_{\texttt {out}}\!\left( r_2(U;\cdot )\right) \in {\widetilde{\mathcal {R}}}_2^{-\varrho }$ is the paradifferential operator of order $-\varrho$ coming from the symbol in (4.20). This proves the identity (4.12), renaming $R_2+R_2'+R_2'' \leadsto R_2$.

Finally we prove that the matrices of smoothing operators are gauge invariant. Indeed each operator on the right of (4.14)–(4.16) is gauge invariant (recall Lemma 4.2), as well as the 2-homogeneous matrix of paradifferential operators in (4.21). Then, by difference, the 2-homogeneous smoothing operators $R_2 + R_2' + R_2''$ are gauge invariant as well. $\square$

### Reduction of the highest order

In this section we perform a transformation that reduces the symbol of the highest order paradifferential operator $\textrm{Op}^{\scriptscriptstyle BW}_{\texttt {vec}}\!\left( \underline{{\mathtt V}}({U};x) \textrm{i}\xi \right)$ to its resonant normal form.

#### Proposition 4.7

(Paracomposition) Let $$\varrho \ge 1$$. There are $$ s_0, r > 0$$ and a 2-admissible transformation $$\Phi (U) \in {{\mathcal {M}}}_{\ge 0}^0[r]$$ with gain 5 (see Definition 2.11) such that if $$U(t) \in B_{ s_0, {{\mathbb {R}}}}(I;r)$$ solves (4.3), then the variable4.224.23where

$\bullet$ ${\mathbf {\Omega }}(D)$ is the matrix of Fourier multipliers defined in (3.17);

$\bullet$ $\langle \,\underline{{\mathtt V}}\, \rangle (U;x)$ is the resonant part of the function $\underline{{\mathtt V}}(U;x)$ in (4.4), namely the zero-average, real valued function in (4.8);

$\bullet$ $V_{\ge 4 }(U;x)$ is a real function in $\mathcal {F}_{\ge 4}^{{{\mathbb {R}}}}[r]$;

$\bullet$ ${ a}_2^{(\alpha )}({U};x,\xi )$ is a zero average, gauge invariant (fulfills the first of (2.26)), real symbol in ${\widetilde{\Gamma }}_2^{\alpha }$ and $a^{(\alpha ) }_{\ge 4} (U;x,\xi )$ a real non-homogeneous symbol in $\Gamma ^\alpha _{\ge 4}[r]$;

$\bullet$ $R_2({U})$ is a real-to-real, gauge invariant matrix of homogeneous smoothing operators in ${\widetilde{\mathcal {R}}}^{-\varrho }_{2}$;

$\bullet$ $B_{\ge 4}(U)$ is a real-to-real matrix of 0-operators in $\mathcal {M}^0_{\ge 4}[r]$.

#### Proof

We define the transformation $\Phi (U)$ as the time-1 flow of the paradifferential equation

4.24 $$\begin{aligned} {\left\{ \begin{array}{ll} \partial _\tau \Phi ^\tau (U)= G(U)\Phi ^\tau (U)\\ \Phi ^0(U)=\textrm{Id}, \end{array}\right. }\quad \text {where} \quad G(U):= \textrm{Op}^{\scriptscriptstyle BW}_{\texttt {vec}}\!\left( \frac{\beta _2(U;x)}{1+\tau (\beta _2)_x(U;x)}\textrm{i}\xi \right) \nonumber \\ \end{aligned}$$

and $\beta _2(U,V)$ the real valued, 2-symmetric function

$$\begin{aligned} \beta _2(U,V;x):= \sum _{j_1, j_2 \in {{\mathbb {Z}}}\atop \sigma _1, \sigma _2 \in {\pm }} \beta _{j_1,j_2}^{\sigma _1,\sigma _2} u_{j_1}^{\sigma _1} \, v_{j_2}^{\sigma _2} \, e^{\textrm{i}(\sigma _1 j_1 + \sigma _2 j_2)x} \end{aligned}$$

where the symmetric coefficients

$$\begin{aligned} \beta _{j_1,j_2}^{\sigma _1,\sigma _2}:={\left\{ \begin{array}{ll} \frac{1}{2\textrm{i}(\sigma _1|j_1|^\alpha +\sigma _2 |j_2|^\alpha )}&  \sigma _1\sigma _2=-1, \ |j_1| \ne |j_2| \\ 0&  \text {otherwise} \end{array}\right. } \end{aligned}$$

fulfill (2.15) with $\mu = 1-\alpha$. Note that

4.25 $$\begin{aligned} \beta _2(U;x):=\beta _2(U,U;x)= \sum _{|j_1| \ne |j_2| } \frac{1}{\textrm{i}(|j_1|^\alpha - |j_2|^\alpha ) }u_{j_1}\overline{u_{j_2}}e^{\textrm{i}( j_1- j_2)x} \end{aligned}$$

By Lemma 2.16, $\Phi$ is a 2-admissible transformation with an arbitrary gain, which again we fix to 5. Moreover, since $\beta _2$ fulfills the first of (2.26), *G* as well as $\Phi ^\tau$, $\tau \in [0,1]$, are gauge invariant (see the bullet of formula (2.26) and Remark 2.15).

Recalling (4.12), the variable $W_1:=\Phi (U)W$ solves

4.26 $$\begin{aligned} \partial _t W_1=&\Phi (U) \textrm{Op}^{\scriptscriptstyle BW}_{\texttt {vec}}\!\left( - \textrm{i}|\xi |^\alpha +\textrm{i}\underline{{\mathtt V}}(U) \xi +\textrm{i}\underline{{\mathtt d}}(U) \right) \Phi (U)^{-1} W_1 \end{aligned}$$

4.27 $$\begin{aligned}&+ \left( \partial _t \Phi (U)\right) \Phi (U)^{-1} W_1 \end{aligned}$$

4.28 $$\begin{aligned}&+ \Phi (U)\left[ R_2(U) + B_{\ge 4}(U)\right] \Phi (U)^{-1} W_1 \ . \end{aligned}$$

We now compute each term, starting from (4.26). By Proposition B.1–2 (with $\varrho \leadsto \varrho +\alpha$) we get

$$\begin{aligned} \Phi (U)&\textrm{Op}^{\scriptscriptstyle BW}_{\texttt {vec}}\!\left( - \textrm{i}|\xi |^\alpha \right) \Phi (U)^{-1} = \textrm{Op}^{\scriptscriptstyle BW}_{\texttt {vec}}\!\left( - \textrm{i}|\xi |^\alpha + {\textrm{i}a^{(\alpha )}_{2}} + \textrm{i}a^{(\alpha ) }_{\ge 4} \right) +B_{\ge 4}(U)+ R'_2(U) \end{aligned}$$

where $a^{(\alpha )}_{2}$ is a real, zero average, gauge invariant symbol in ${\widetilde{\Gamma }}^\alpha _2$, $a^{(\alpha )}_{\ge 4}$ is a real symbol in $\Gamma _{\ge 4}^\alpha [r]$, $B_{\ge 4 }= \textrm{Op}^{\scriptscriptstyle BW}_{\texttt {vec}}\!\left( \textrm{i}a_{\ge 4}^{(\alpha -2)}\right) + R_{\ge 4}$ (see (B.3)) is a real-to-real matrix of 0-operators in $\mathcal {M}^0_{\ge 4}[r]$ and finally $R'_2(U)$ is a real-to-real, gauge invariant matrix of smoothing operators in ${\widetilde{{{\mathcal {R}}}}}_2^{-\varrho }$.

Then, by Proposition B.1–1, we get

$$\begin{aligned} \Phi (U)&\textrm{Op}^{\scriptscriptstyle BW}_{\texttt {vec}}\!\left( \textrm{i}\underline{{\mathtt V}}\xi + \textrm{i}\, \underline{{\mathtt d}}\right) \Phi (U)^{-1} = \textrm{Op}^{\scriptscriptstyle BW}_{\texttt {vec}}\!\left( \textrm{i}\underline{{\mathtt V}}\xi +\textrm{i}V'_{\ge 4 } \xi + \textrm{i}\, \underline{{\mathtt d}}\right) + B_{\ge 4}(U) \end{aligned}$$

with $V'_{\ge 4 } \in {{\mathcal {F}}}^{{\mathbb {R}}}_{\ge 4}[r]$ and, thanks to $\varrho \ge 1$, $B_{\ge 4 }$ a real-to-real matrix of 0-operators in $\mathcal {M}^0_{\ge 4}[r]$.

Next we consider the term in (4.27). We apply Proposition B.1–4 and get

$$\begin{aligned} \big ( \partial _t \Phi (U) \big ) \Phi (U)^{-1}= \textrm{Op}^{\scriptscriptstyle BW}_{\texttt {vec}}\!\left( 2\textrm{i}\beta _2(-\textrm{i}{\mathbf {\Omega }}(D)U, U) \xi + \textrm{i}V''_{\ge 4}(U)\xi \right) + B_{\ge 4 }(U) \end{aligned}$$

where $V''_{\ge 4} \in {{\mathcal {F}}}^{{\mathbb {R}}}_{\ge 4}[r]$ and, using again $\varrho \ge 1$, $B_{\ge 4 }$ a real-to-real matrix of 0-operators in $\mathcal {M}^0_{\ge 4}[r]$.

Finally we consider line (4.28). By Proposition B.1–3 and Remark 2.12—(2)

$$\begin{aligned} (4.28) = R_2(U) + B_{\ge 4}(U) \end{aligned}$$

with $$R_2(U) $$ the same real-to-real, gauge invariant matrix of smoothing operators in $${\widetilde{{{\mathcal {R}}}}}_2^{{- \varrho }}$$ of Proposition 4.5 and with $$B_{\ge 4 }$$ a real-to-real matrix of 0-operators in $$\mathcal {M}^0_{\ge 4}[r]$$.

Altogether we have the expansion

$$\begin{aligned} \partial _t W_1&= \textrm{Op}^{\scriptscriptstyle BW}_{\texttt {vec}}\!\left( - \textrm{i}|\xi |^\alpha + \textrm{i}\underline{{\mathtt V}}\xi + 2\textrm{i}\beta _2(-\textrm{i}{\mathbf {\Omega }}(D)U, U) \xi + \textrm{i}a^{(\alpha )}_2 \right) W_{1} \\  &\quad + ( R_2(U)+ R_2'(U)) W_{1} \\&+ \textrm{Op}^{\scriptscriptstyle BW}_{\texttt {vec}}\!\left( \textrm{i}V_{\ge 4} \xi + \textrm{i}a^{(\alpha ) }_{\ge 4} \right) W_{1} + B_{\ge 4}(U)W_{1} \ . \end{aligned}$$

One verifies that $\beta _2$ in (4.25) solves the homological equation

$$\begin{aligned} 2 \beta _2(-\textrm{i}{\mathbf {\Omega }}(D)U,U;x)+ \underline{{\mathtt V}}(U;x)= \langle \, \underline{{\mathtt V}}\, \rangle ({U};x) \ , \end{aligned}$$

using the expressions of $\underline{{\mathtt V}}$ in (4.4), ${\mathbf {\Omega }}(D)$ in (3.17), and $\langle \, \underline{{\mathtt V}}\, \rangle$ in (4.8). This proves the expansion in (4.23), renaming $R_2 + R_2'\leadsto R_2$; note that we proved that it is gauge invariant being sum of gauge invariant operators. $\square$

### The weak $\Lambda$-normal form

In this section we perform a Poincaré normal form, with the goal of putting the smoothing operator $R_2(U)W_1$ in (4.23) into weak-$\Lambda$ normal form (see Definition (4.1)).

#### Proposition 4.8

(Weak-$$\Lambda $$ normal form) Let $$\varrho \ge 2-\alpha $$. There are $$ s_0, r > 0$$ and a 0-admissible transformation $$\Upsilon (U) \in {{\mathcal {M}}}_{\ge 0}^0[r]$$ with gain $$\varrho -1+ \alpha $$ (see Definition 2.11) such that if $$U(t) \in B_{ s_0, {{\mathbb {R}}}}(I;r)$$ solves (4.3), then the variable

4.29 $$\begin{aligned} Z:= \Upsilon (U)W_1 {\mathop {=}\limits ^{(4.22), (4.11)}} \Upsilon (U)\Phi (U)\Psi (U)U \quad \text{ solves } \end{aligned}$$

4.30 $$\begin{aligned} \partial _t Z=&- \textrm{i}{\mathbf {\Omega }}(D)Z + \left( \textrm{i}\langle \,\underline{{\mathtt V}}\, \rangle (U;x) \xi + \textrm{i}V_{\ge 4 }(U;x) \xi + \textrm{i}{ a}^{(\alpha ) }_2(U;x,\xi ) \right. \nonumber \\  &\quad \left. { + \textrm{i}a^{(\alpha ) }_{\ge 4} (U;x,\xi )}\right) Z + {R}_2^{(\Lambda )}({U})Z + {B}_{\ge 4}({U}) Z, \end{aligned}$$

where $\langle \,\underline{{\mathtt V}}\, \rangle$, $V_{\ge 4 }$, ${ a}^{(\alpha ) }_2$ and $a^{(\alpha ) }_{\ge 4}$ are the same symbols of Proposition 4.7, whereas

$\bullet$ ${R}_2^{(\Lambda )}({U})$ is a real-to-real, gauge invariant matrix of smoothing operators in ${\widetilde{{{\mathcal {R}}}}}_2^{-\varrho }$ such that the cubic vector field $X^{(\Lambda )}(Z):= {R}_2^{(\Lambda )}(Z)Z$ is in weak-$\Lambda$ normal form, namely, it fulfills that

4.31 $$\begin{aligned} \Pi _{{\mathcal {P}}^{(n)}_\Lambda } X^{(\Lambda )} = \Pi _{\textsf{R}^{(n)}_\Lambda } X^{(\Lambda )} \ , \quad n = 0,1,2 \ . \end{aligned}$$

$\bullet$ $B_{\ge 4}(U)$ is a real-to-real matrix of 0-operators in ${{\mathcal {M}}}_{\ge 4 }^0[r]$.

#### Proof

We look for a transformation $\Upsilon (U)$ as the time-1 flow of the equation

$$\begin{aligned} \partial _\tau \Upsilon ^\tau (U) = \texttt {Q}_2(U) \Upsilon ^\tau (U) , \quad \Upsilon ^0(U) = \textrm{Id}\end{aligned}$$

where $\texttt {Q}_2$ is a matrix of smoothing operators in ${\widetilde{\mathcal {R}}}^{-\varrho +1-\alpha }_{2}$ to be determined. By Lemma 2.17, the map $\Upsilon ^\tau$ is a 0-admissible transformation with gain $\varrho -1+ \alpha$ which is non-negative. Recalling (4.23), the variable $Z:= \Upsilon (U) W_1$ fulfills

$$\begin{aligned} \begin{aligned} \partial _t Z=&\Upsilon (U) \left( - \textrm{i}{\mathbf {\Omega }}(D) \right) \Upsilon (U)^{-1} Z + \Upsilon (U) \textrm{Op}^{\scriptscriptstyle BW}_{\texttt {vec}}\!\left( \textrm{i}\, \texttt{m}^{(1)}\right) \Upsilon (U)^{-1} Z \\&+ \Upsilon (U) \big ( R_2({U}) + B_{\ge 4}(U) \big ) \Upsilon (U)^{-1} Z +\big (\partial _t \Upsilon (U) \big ) \Upsilon (U)^{-1} Z, \end{aligned} \end{aligned}$$

where we set $\texttt{m}^{(1)}:= \langle \, \underline{{\mathtt V}}\, \rangle \xi + a_2^{(\alpha )} + {V}_{\ge 4 } \xi + { a^{(\alpha )}_{\ge 4}}\in \Sigma \Gamma ^1_{2}[r]$. By Proposition B.2 (with $\varrho \leadsto \varrho -(1-\alpha )$), we get

4.32 $$\begin{aligned} \begin{aligned} \partial _t Z=&- \textrm{i}{\mathbf {\Omega }}(D)Z + \textrm{Op}^{\scriptscriptstyle BW}_{\texttt {vec}}\!\left( \textrm{i}\texttt{m}^{(1)}\right) Z\\&+2 {\mathtt Q}_2\left( - \textrm{i}{\mathbf {\Omega }}(D) U, U\right) Z + [ {\mathtt Q}_2(U), - \textrm{i}{\mathbf {\Omega }}(D) ] Z + R_2({U})Z \\&+ B_{\ge 4}(U) Z+ R_{\ge 4}(U)Z , \end{aligned} \end{aligned}$$

where $B_{\ge 4}({U})$ is a real-to-real matrix of 0-operators in ${{\mathcal {M}}}^0_{\ge 4}[r]$ and $R_{\ge 4}(U)$ is a real-to-real matrix of smoothing operators in $\mathcal {R}^{-\varrho +2-\alpha }_{\ge 4}[r]$ which we shall regard as a 0-operator in $\mathcal {M}_{\ge 4}^0[r]$ since $\varrho \ge 2-\alpha$.

To determine ${\mathtt Q}_2(U)$, expand the vector field $R_2(U) Z$ in (4.23) in Fourier components as

$$\begin{aligned} ( R_2(U) Z)^\sigma _k = \sum _{ \mathcal {P}_4 } R_{j_1, j_2, j, k}^{\sigma _1, \sigma _2, \sigma ', \sigma } u_{j_1}^{\sigma _1} \, u_{j_2}^{\sigma _2} z_{j}^{\sigma '}, \end{aligned}$$

where with the sum over $\mathcal {P}_4$ we mean that the indexes $(j_1, j_2, j, k,\sigma _1, \sigma _2, \sigma ', -\sigma )$ belong to $\mathcal {P}_4$. Below we use the same notation. Note that this writing is possible since $R_2(U)$ is gauge invariant.

Then we define

$$\begin{aligned}(R^{(\Lambda )}_2(U)Z)^\sigma _k:= &   \sum _{\mathcal {P}_4 } \Lambda _{j_1, j_2, j, k}^{\sigma _1, \sigma _2, \sigma ', \sigma } u_{j_1}^{\sigma _1} \, u_{j_2}^{\sigma _2} z_{j}^{\sigma '}, \quad \\ \Lambda _{j_1, j_2, j, k}^{\sigma _1, \sigma _2, \sigma ', \sigma }:= &   R_{j_1, j_2, j, k}^{\sigma _1, \sigma _2, \sigma ', \sigma } \delta \Big ( (j_1, j_2, j, k,\sigma _1, \sigma _2, \sigma ', -\sigma )\in \mathcal {C}\Big ), \end{aligned}$$

where $\mathcal {C}:=\bigcup \limits _{n =0}^2 \textsf{R}^{(n)}_\Lambda \cup \bigcup \limits _{n=3}^4 {\mathcal {P}}^{(n)}_\Lambda$, and the sets ${\mathcal {P}}^{(n)}_\Lambda$ , $\textsf{R}^{(n)}_\Lambda$ defined in (3.4), (3.5). We choose ${\mathtt Q}_2(U)$ so that

4.33 $$\begin{aligned} 2{\mathtt Q}_2\left( - \textrm{i}{\mathbf {\Omega }}(D) U,U\right) + [ {\mathtt Q}_2(U), - \textrm{i}{\mathbf {\Omega }}(D) ] + R_2({U}) = R^{(\Lambda )}_2(U). \end{aligned}$$

We claim that one can set, denoting that ${\jmath }=(j_1, j_2)$, $\mathbf {\sigma }=(\sigma _1, \sigma _2)$, that

4.34 $$\begin{aligned} ( {\mathtt Q}_2(U)Z )^\sigma _k := \sum _{ {{\mathcal {P}}}_4} {\mathtt Q}_{{\jmath } , j, k}^{\mathbf {\sigma }, \sigma ', \sigma } \, u_{j_1}^{\sigma _1} \, u_{j_2}^{\sigma _2} z_{j}^{\sigma '}, \end{aligned}$$

where

4.35 $$\begin{aligned} {\mathtt Q}_{{\jmath }, j, k}^{\mathbf {\sigma }, \sigma ', \sigma } := {\left\{ \begin{array}{ll} \dfrac{R_{{\jmath }, j, k}^{\mathbf {\sigma }, \sigma ', \sigma }}{\textrm{i}(\sigma _1 |j_1|^\alpha +\sigma _2 |j_2|^\alpha + {\sigma '} |j|^\alpha - \sigma |k|^\alpha )} \ , &  ({\jmath },j, k, \mathbf {\sigma }, \sigma ', -\sigma ) \in \bigcup \limits _{n =1}^2 \left( {\mathcal {P}}^{(n)}_\Lambda \setminus \textsf{R}^{(n)}_\Lambda \right) \\ 0 \ , &  ({\jmath }, j, k, \mathbf {\sigma }, \sigma ', -\sigma ) \in \mathcal {C}\end{array}\right. }.\nonumber \\ \end{aligned}$$

#### Lemma 4.9

${\mathtt Q}_2(U)$ in (4.34)–(4.35) is a matrix of smoothing operators in ${\widetilde{{{\mathcal {R}}}}}^{-\varrho + 1-\alpha }_2$ fulfilling (4.33).

#### Proof

As $R_2(U)$ is a smoothing operator in ${\widetilde{{{\mathcal {R}}}}}^{-\varrho }_2$, its coefficients fulfill the estimate: for some $\mu \ge 0$, $C>0$,

4.36 $$\begin{aligned} \left| R_{{\jmath }, j, k}^{\mathbf {\sigma }, \sigma ', \sigma } \right| \le C \frac{ \textrm{max}_2\{ \langle j_1\rangle , \langle j_2 \rangle , \langle j\rangle \}^{\mu }}{ \max \{ \langle j_1\rangle , \langle j_2 \rangle , \langle j\rangle \}^{\varrho }} \, , \quad \forall ({\jmath }, j, k, \mathbf {\sigma }, \sigma ', -\sigma ) \in {\mathcal {P}}_4 , \qquad \end{aligned}$$

and satisfy the symmetric and reality properties (2.10) and (2.11).

Consider now the coefficients ${\mathtt Q}_{{\jmath }, j, k}^{\mathbf {\sigma }, \sigma ', \sigma }$ in (4.35). Clearly they satisfy the symmetric and reality properties (2.10) and (2.11). We now bound them. By (4.36), Lemma 3.3 and the momentum relation $\sigma k= \sigma _1 j_1 + \sigma _2 j_2 + \sigma ' j$,

$$\begin{aligned} \left| {\mathtt Q}_{{\jmath }, j, k}^{\mathbf {\sigma }, \sigma ', \sigma } \right| \le C \frac{ \textrm{max}_2\{ \langle j_1\rangle ,\langle j_2 \rangle , \langle j_3\rangle \}^{\mu }}{ \max \{ \langle j_1\rangle ,\langle j_2 \rangle , \langle j_3\rangle \}^{\varrho - (1-\alpha )}}\qquad \forall ({\jmath } , j, k, \mathbf {\sigma }, \sigma ', -\sigma ) \in {\mathcal {P}}^{(1)}_{\Lambda } \cup ({\mathcal {P}}^{(2)}_\Lambda \setminus \textsf{R}^{(2)}_\Lambda ) \end{aligned}$$

(recall that $\textsf{R}^{(1)}_\Lambda = \emptyset$). This shows that ${\mathtt Q}_2(U)$ is a matrix of smoothing operators in ${\widetilde{{{\mathcal {R}}}}}_2^{-\varrho + 1-\alpha }$.

It is clear that ${\mathtt Q}_2(U)$ fulfills (4.33), also noting that $\Pi _{{\mathcal {P}}^{(0)}_\Lambda }(R_2(Z)Z) =\Pi _{\textsf{R}^{(0)}_\Lambda }(R_2(Z)Z)$ in view of Lemma 3.3 (*i*). $\square$

With such ${\mathtt Q}_2(U)$, system (4.32) reduces to (4.30).

We prove now that the vector field $X^{(\Lambda )}(Z)= {R}_2^{(\Lambda )}(Z)Z$ is in weak-$\Lambda$ normal form, i.e. it fulfills (4.31). Indeed the coefficients of the vector field $X^{(\Lambda )}$ are obtained as in (2.37) and, being the set $\mathcal {C}$ symmetric with respect to the first three indexes, they have the form

$$\begin{aligned} X_{j_1, j_2, j_3, k}^{\sigma _1, \sigma _2, \sigma _3, \sigma }= &   \frac{1}{3} \Big ( R_{j_1, j_2, j_3, k}^{\sigma _1, \sigma _2, \sigma _3, \sigma } + R_{j_3, j_2, j_1, k}^{\sigma _3, \sigma _2, \sigma _1, \sigma }+ R_{j_1, j_3, j_2, k}^{\sigma _1, \sigma _3, \sigma _2, \sigma } \Big ) \\  &   \, \delta \Big ( (j_1, j_2, j_3, k,\sigma _1, \sigma _2, \sigma _3, -\sigma ) \in \mathcal {C}\Big ) \ . \end{aligned}$$

Proposition 4.8 is proven. $\square$

### Identification and proof of Theorem 4.4

With the aid of paradifferential normal form, we have conjugated the original system (4.1) to the new system (4.30). The next steps are: (*i*) to write (4.30) as a system in the single variable *Z*(*t*), and (*ii*) to compute explicitly $\Pi _{{\mathcal {P}}^{(n)}_\Lambda }X^{(\Lambda )}$ in (4.31) for $n = 0,1,2$, deducing (4.10).

To achieve (*i*), recall that the map in (4.29) has the form

4.37 $$\begin{aligned} Z = {{\mathcal {F}}}(U)= \textbf{F}(U)U , \quad \textbf{F}(U):= \Upsilon (U) \Phi (U) \Psi (U) \ . \end{aligned}$$

Since $\Upsilon (U)$ is 0-admissible with gain $\varrho - 1 + \alpha$, $\Phi (U)$ is 2-admissible with gain 5 and $\Psi (U)$ is 0-admissible with gain 5 (Propositions 4.5, 4.7, 4.8), by Lemma 2.13 the map $\textbf{F}(U)$ a 2-admissible with gain $\min (3, \varrho - 1 + \alpha ) = 3$ provided $\varrho \ge 4- \alpha$.

Then Lemma 2.14 ensures that ${{\mathcal {F}}}$ is locally invertible in a small ball $B_{s_0'}(r')$ for some $s_0', r'>0$, with inverse map ${{\mathcal {F}}}^{-1}$ having the structure

4.38 $$\begin{aligned} U = {{\mathcal {F}}}^{-1}(Z) = \textbf{G}(Z) Z , \quad \text{ with } \textbf{G}(Z) = \textrm{Id}+ \textbf{G}_{\ge 2}(Z) , \ \ \textbf{G}_{\ge 2}(Z) \in \Sigma {{\mathcal {M}}}^{4}_{2}[r'] ,\nonumber \\ \end{aligned}$$

for some $r' >0$. We then substitute *U* in the internal variables of the operators in (4.30). Consider first the 2-homogeneous operators. We have, using Lemma 2.10–1,

$$\begin{aligned}  &   \langle \,\underline{{\mathtt V}}\, \rangle ({{\mathcal {F}}}^{-1}(Z);x) \xi - \langle \,\underline{{\mathtt V}}\, \rangle (Z;x) \xi \in \Gamma ^{1}_{\ge 4}[r'] , \quad \\    &   {a}^{(\alpha )}_2({{\mathcal {F}}}^{-1}(Z);x,\xi ) - {a}^{(\alpha )}_2(Z;x,\xi ) \in \Gamma ^{\alpha }_{\ge 4}[r'], \end{aligned}$$

and, using Lemma 2.10–2, ${R}_2^{(\Lambda )}({{\mathcal {F}}}^{-1}(Z)) - {R}_2^{(\Lambda )}(Z) \in {{\mathcal {R}}}_{\ge 4}^{- \varrho +4}[r']$. Then we substitute $U={{\mathcal {F}}}^{-1}(Z)$ in the non-homogeneous operators $\textrm{Op}^{\scriptscriptstyle BW}_{\texttt {vec}}\!\left( \textrm{i}V_{\ge 4 }(U;x) \xi + \textrm{i}a^{(\alpha ) }_{\ge 4} (U;x,\xi )\right)$ and $B_{\ge 4}(U)$, applying Lemma 2.10–1& 5. In conclusion, setting $\varrho :=4$, we obtain the following:

#### Proposition 4.10

There are $$ s_0, r > 0$$ such that if $$U(t) \in B_{ s_0, {{\mathbb {R}}}}(I;r)$$ solves (4.3), then the variable *Z*(*t*) in (4.37) solves the system

4.39 $$\begin{aligned} \partial _t Z=&- \text {i}{\mathbf {\Omega }}(D)Z + \text {Op}^{\scriptscriptstyle BW}_{\texttt {vec}}\!\left( \text {i}\langle \,\underline{{\mathtt V}}\, \rangle (Z;x) \xi + \text {i}{ a}^{(\alpha ) }_2(Z;x,\xi )\right) Z + {X}^{(\Lambda )}(Z) \nonumber \\  &+ \text {Op}^{\scriptscriptstyle BW}_{\texttt {vec}}\!\left( \text {i}{\widetilde{V}}_{\ge 4 }(Z;x) \xi + \text {i}{\widetilde{a}}^{(\alpha ) }_{\ge 4} (Z;x,\xi )\right) Z + {\widetilde{B}}_{\ge 4}(Z) Z \end{aligned}$$

where $\langle \,\underline{{\mathtt V}}\, \rangle$ and ${ a}^{(\alpha ) }_2$ are the quadratic symbols in Proposition 4.7, ${X}^{(\Lambda )}(Z)$ is the cubic vector field in weak-$\Lambda$ normal form of Proposition 4.8 is , whereas

- ${\widetilde{V}}_{\ge 4 }(Z;x)$ is a real function in $\mathcal {F}_{\ge 4}^{{{\mathbb {R}}}}[r]$;
- ${\widetilde{a}}^{(\alpha ) }_{\ge 4} (Z;x,\xi )$ is a real non-homogeneous symbol in $\Gamma ^\alpha _{\ge 4}[r]$;
- ${\widetilde{B}}_{\ge 4}(Z)$ is a real-to-real matrix of 0-operators in ${{\mathcal {M}}}_{\ge 4 }^0[r]$.

The next step (*ii*) is to compute explicitly $$\Pi _{{\mathcal {P}}^{(n)}_\Lambda }{X}^{(\Lambda )}$$, $$ n = 0,1,2$$:

#### Proposition 4.11

The vector field ${X}^{(\Lambda )}(Z)$ of Proposition 4.8 is actually in strong-$\Lambda$ normal form (Definition 4.1) and fulfills (4.10).

#### Proof

We combine the abstract identification argument of Proposition  3.6 with the characterization of the resonant monomials of the original vector field $X_3$ in Lemma 3.4.

Precisely, we apply the identification result of Proposition  3.6 to the starting NLS equation (4.1) (which has the required structure in (3.18) in view of (4.2)) and with the admissible transformation $\textbf{F}(U)$ in (4.37), getting that *Z* fulfills an equation of the form (3.19). Identifying the cubic vector field of (3.19) with the one of (4.39) we get the identity

$$\begin{aligned} \text {Op}^{\scriptscriptstyle BW}_{\texttt {vec}}\!\left( \text {i}\langle \,\underline{{\mathtt V}}\, \rangle (Z;x) \xi + \text {i}{ a}^{(\alpha ) }_2(Z;x,\xi )\right) Z + {X}^{(\Lambda )}(Z) = {\widetilde{X}}_3(Z) \ . \end{aligned}$$

In addition, in view of (3.20), we have

4.40 $$\begin{aligned} \Pi _{\textsf{R}^{(n)}_{\Lambda } }\left( \textrm{Op}^{\scriptscriptstyle BW}_{\texttt {vec}}\!\left( \textrm{i}\langle \,\underline{{\mathtt V}}\, \rangle (Z;x) \xi + \textrm{i}{ a}^{(\alpha ) }_2(Z;x,\xi )\right) Z+ {X}^{(\Lambda )} \right) =\Pi _{\textsf{R}^{(n)}_{\Lambda } } X_ 3 \ , \quad n = 0,1,2 \ .\nonumber \\ \end{aligned}$$

Now we apply Lemma 3.5 to the cubic vector field $\textrm{Op}^{\scriptscriptstyle BW}_{\texttt {vec}}\!\left( \textrm{i}\langle \,\underline{{\mathtt V}}\rangle \, \xi + \textrm{i}{ a}^{(\alpha )}_2\right) Z$; this can be done since the symbols $\langle \,\underline{{\mathtt V}}\, \rangle (Z;x) \xi$ and ${ a}^{(\alpha ) }_2(Z;x,\xi )$ have both zero-average (Proposition 4.7) and are gauge invariant (i.e. fulfills the first of (2.26)). We conclude that

4.41 $$\begin{aligned} \Pi _{\textsf{R}^{(n)}_{\Lambda } } \left[ \textrm{Op}^{\scriptscriptstyle BW}_{\texttt {vec}}\!\left( \textrm{i}\langle \,\underline{{\mathtt V}}\, \rangle (Z;x) \xi + \textrm{i}{ a}^{(\alpha ) }_2(Z;x,\xi )\right) Z \right] = 0 \ , \quad n = 0,1,2 \ , \end{aligned}$$

from which we immediately get that

$$\begin{aligned}&\Pi _{{\mathcal {P}}^{(n)}_{\Lambda } }{X}^{(\Lambda )} {\mathop {=}\limits ^{(4.31)}} \Pi _{\textsf{R}^{(n)}_{\Lambda } }{X}^{(\Lambda )} {\mathop {=}\limits ^{(4.40), (4.41)}} \Pi _{\textsf{R}^{(n)}_{\Lambda }} {X}_3 \ , \quad n = 0,1,2 \ . \end{aligned}$$

This last vector field is computed in Lemma 3.4, proving (4.10).

$$\square $$

#### Proof of Theorem 4.4

This follows from Proposition 4.10 and 4.11. $\square$

## The Effective Equation

The goal of this section is to study the long-time dynamics of solutions of equation (4.7) fulfilling certain upper-bounds, that we call *long-time controlled*, see Definition 5.2. In view of the reality of system (4.7), we regard it as a scalar equation in *z*(*t*). We study separately the dynamics of the modes supported on $\Lambda$, namely $z_{\pm 1}(t)$, and those supported on $\Lambda ^c$. More specifically, we decompose

5.1 $$\begin{aligned} z(t)= &   z^\top (t) + z^\perp (t) \ , \quad z^\top (t) := z_1(t) \, e^{\textrm{i}x} + z_{-1}(t) \, e^{-\textrm{i}x} \ , \quad \nonumber \\ z^\perp (t):= &   \sum _{|j| \ne 1} z_j(t) \, e^{\textrm{i}j x}. \end{aligned}$$

$$\bullet $$ **Parameters:** From now on we **fix** $$\mathfrak {s}_0, {\mathfrak {r}}>0$$ as follows: $${\mathfrak {s}}_0:=\max \{ s_0, s_0'\}$$ and $${\mathfrak {r}}:=\min \{r,r'\}$$ where $$s_0, r >0 $$ are given in Theorem 4.4 whereas $$s_0', r'>0$$ are the parameters required to invert the map $$ \mathcal {F}$$ in (4.6), see (4.38). We also fix that

5.2 $$\begin{aligned} s> 3 {\mathfrak {s}}_0 , \quad \theta \in (0, \theta _*), \ \ \theta _* := \min \left( \frac{s-3 {\mathfrak {s}}_0}{2s - {\mathfrak {s}}_0}, \frac{1}{5} \right) \ . \end{aligned}$$

The first step is the following one:

### Lemma 5.1

If $$Z(t)=\Big ({\begin{smallmatrix}z(t) \\ {\overline{z}}(t)\end{smallmatrix}}\Big ) \in B_{{\mathfrak {s}}_0, {{\mathbb {R}}}}(I;{\mathfrak {r}})$$ solves (4.7), then the variables $\big (z^\top (t), z^\perp (t)\big )$ defined in (5.1) fulfill the system

5.3 $$\begin{aligned} \partial _t z^\top =&- \textrm{i}|D|^\alpha z^\top + Y_3^{(\Lambda )}(z^\top ) + Y^\top _3 (z) + Y^\top _{\ge 5}(z) \end{aligned}$$

5.4 $$\begin{aligned} \partial _t z^\perp =&- \textrm{i}|D|^\alpha z^\perp + \textrm{Op}^{\!\scriptscriptstyle BW}\!\left( \textrm{i}\, \texttt{m}(z; x, \xi ) \right) z^\perp +Y^\perp _3(z) + Y^\perp _{\ge 5}(z) \end{aligned}$$

where

$\bullet$ $Y_3^{(\Lambda )}(z)$ is the integrable vector field

5.5 $$\begin{aligned} Y^{(\Lambda )}_3 (z) := Y^{(\Lambda )}_3 (z^\top ) = - \textrm{i}|z_1|^2 z_1 \, e^{\textrm{i}x} + \textrm{i}|z_{-1}|^2 z_{-1} \, e^{-\textrm{i}x} \ ; \end{aligned}$$

$\bullet$ $Y^\top _3(z)$ and $Y^\perp _3(z)$ are cubic smoothing vector fields fulfilling: for any ${\mathtt s}\ge {\mathfrak {s}}_0$

5.6 $$\begin{aligned} \Vert Y^\top _3(z) \Vert _{{\mathtt s}} \lesssim \Vert z^\perp \Vert _{{\mathfrak {s}}_0}^3 \, \ , \qquad \Vert Y^\perp _3(z) \Vert _{{\mathtt s}+ 4} \lesssim \left( \Vert z^\top \Vert _{{\mathfrak {s}}_0} + \Vert z^\perp \Vert _{{\mathfrak {s}}_0} \right) \, \Vert z^\perp \Vert _{{\mathfrak {s}}_0} \, \Vert z^\perp \Vert _{\mathtt s}\ ;\nonumber \\ \end{aligned}$$

$\bullet$ $\texttt{m}(z;x, \xi )$ is the symbol in $\Sigma \Gamma ^1_{\ge 2}[{\mathfrak {r}}]$ given by

5.7 $$\begin{aligned} \texttt{m}(z;x, \xi ):= \langle \,\underline{{\mathtt V}}\, \rangle (Z;x) \xi + {a}^{(\alpha )}_2(Z;x,\xi ) + {\widetilde{V}}_{\ge 4 }(Z;x) \xi + {\widetilde{a}}^{(\alpha ) }_{\ge 4} (Z;x,\xi ) \ ,\qquad \end{aligned}$$

with $\langle \,\underline{{\mathtt V}}\, \rangle (Z;x)$ defined in (4.8).

$\bullet$ $Y^\top _{\ge 5}(z)$ and $Y^\perp _{\ge 5}(z)$ are non-homogeneous vector fields fulfilling the estimate: for any ${\mathtt s}\ge {\mathfrak {s}}_0$ there are $C>0$, ${\mathtt r}:={\mathtt r}({\mathtt s}) \in (0,{\mathfrak {r}})$ and for any $z\in B_{{\mathfrak {s}}_0}({\mathtt r})\cap {H}^{{\mathtt s}}({{\mathbb {T}}},{{\mathbb {C}}})$,

5.8 $$\begin{aligned} \Vert Y^\top _{\ge 5}(z) \Vert _{{\mathtt s}} + \Vert Y^\perp _{\ge 5}(z) \Vert _{{\mathtt s}} \le C \Vert z \Vert ^4_{{\mathfrak {s}}_0} \Vert z \Vert _{\mathtt s}\ . \end{aligned}$$

### Proof

We introduce the projectors

$$\begin{aligned} \Pi ^\top z := \sum _{j = \pm 1} z_j \, e^{\textrm{i}j x} \ , \quad \Pi ^\perp z := \sum _{j \ne \pm 1} z_j \, e^{\textrm{i}j x} \ \end{aligned}$$

and compute the projections of the first component of each term in system (4.7). Since $(-\textrm{i}{\mathbf {\Omega }}(D))^+ = -\textrm{i}|D|^\alpha$ is a Fourier multiplier, it commutes with the projectors. So consider the paradifferential vector field $\big (\textrm{Op}^{\scriptscriptstyle BW}_{\texttt {vec}}\!\left( \textrm{i}\texttt{m}\right) Z \big )^+ = \textrm{Op}^{\!\scriptscriptstyle BW}\!\left( \textrm{i}\, \texttt{m}\right) z$. We decompose

$$\begin{aligned} \textrm{Op}^{\!\scriptscriptstyle BW}\!\left( \textrm{i}\, \texttt{m}\right)&= \Pi ^\top \textrm{Op}^{\!\scriptscriptstyle BW}\!\left( \textrm{i}\, \texttt{m}\right) \Pi ^\top + \Pi ^\top \textrm{Op}^{\!\scriptscriptstyle BW}\!\left( \textrm{i}\, \texttt{m}\right) \Pi ^\perp + \Pi ^\perp \textrm{Op}^{\!\scriptscriptstyle BW}\!\left( \textrm{i}\, \texttt{m}\right) \Pi ^\top + \Pi ^\perp \textrm{Op}^{\!\scriptscriptstyle BW}\!\left( \textrm{i}\, \texttt{m}\right) \Pi ^\perp \ . \end{aligned}$$

Writing $\texttt{m}_2(z; x, \xi ):= \langle \,\underline{{\mathtt V}}\, \rangle (Z;x) \xi + {a}^{(\alpha )}_2(Z;x,\xi )$, $\texttt{m}_{\ge 4}(z; x, \xi ):= {\widetilde{V}}_{\ge 4 }(Z;x) \xi + {\widetilde{a}}^{(\alpha ) }_{\ge 4} (Z;x,\xi )$, we claim that

5.9 $$\begin{aligned}&\Pi ^\top \textrm{Op}^{\!\scriptscriptstyle BW}\!\left( \textrm{i}\, \texttt{m}\right) \Pi ^\top = \Pi ^\top \textrm{Op}^{\!\scriptscriptstyle BW}\!\left( \textrm{i}\, \texttt{m}_{\ge 4} \right) \Pi ^\top \ , \end{aligned}$$

5.10 $$\begin{aligned}&\Pi ^\top \textrm{Op}^{\!\scriptscriptstyle BW}\!\left( \textrm{i}\, \texttt{m}\right) \Pi ^\perp = \Pi ^\perp \textrm{Op}^{\!\scriptscriptstyle BW}\!\left( \textrm{i}\, \texttt{m}\right) \Pi ^\top = 0 \ , \end{aligned}$$

5.11 $$\begin{aligned}&\Pi ^\perp \textrm{Op}^{\!\scriptscriptstyle BW}\!\left( \textrm{i}\, \texttt{m}\right) \Pi ^\perp = \textrm{Op}^{\!\scriptscriptstyle BW}\!\left( \textrm{i}\, \texttt{m}\right) \Pi ^\perp \ . \end{aligned}$$

Proof of (5.9). We shall exploit that the symbol $\texttt{m}_2(z; x, \xi )$ has zero average in *x* (see Theorem 4.4). Using the definition (2.22) for 2-homogeneous paradifferential operators applied to the quadratic, gauge invariant, zero-average symbol $\texttt{m}_2(z; \cdot )$ we get

$$\begin{aligned} \begin{aligned} \Pi ^\top \textrm{Op}^{\!\scriptscriptstyle BW}\!\left( \textrm{i}\, \texttt{m}_2 (z; x, \xi )\right) \Pi ^\top z =&\!\!\!\!\!\! \sum _{ j_1-j_2 +j=k \atop j_1 \ne j_2, \ j, k \in \Lambda } \!\!\! \chi _2 \left( j_1, j_2,\frac{j+k}{2}\right) \textrm{i}\, \texttt{m}_{j_1, j_2}^{+,-}\left( \frac{j+k}{2}\right) z_{j_1} {\overline{z}}_{j_2} z_j \, {e^{\textrm{i}k x}} \ . \end{aligned} \end{aligned}$$

We show that the cut-off is always vanishing. Indeed, recalling that $$\chi _2(\xi ', \xi ) \equiv 0$$ when $$|\xi '| \equiv \max (|\xi '_1|, |\xi '_2|) \ge \langle \xi \rangle /10$$, and using $$\max (|j_1|, |j_2|) \ge 1$$ (as $$j_1, j_2$$ cannot be both 0), $$ j = k-j_1 +j_2$$ and $$k \in \Lambda =\{\pm 1\}$$, one has

5.12 $$\begin{aligned}  &   \frac{1}{10}{\left\langle \frac{j_1 - j_2 \pm 2}{2} \right\rangle } = \frac{1}{10}\left( 1+ \frac{|j_1 - j_2 \pm 2|}{2} \right) \le \frac{4 + 2 \max (|j_1|, |j_2|)}{20} \nonumber \\  &   \quad \le \frac{3 \max (|j_1|, |j_2|)}{10} \le \max (|j_1|, |j_2|) \ , \end{aligned}$$

proving that $\chi _2 \left( j_1, j_2,\frac{j+k}{2}\right) \equiv 0$. Consequently $\Pi ^\top \textrm{Op}^{\!\scriptscriptstyle BW}\!\left( \textrm{i}\, \texttt{m}_2 \right) \Pi ^\top =0$ and (5.9) follows.

Proof of (5.10). Again we write explicitly the action of $\Pi ^\top \textrm{Op}^{\!\scriptscriptstyle BW}\!\left( \textrm{i}\, \texttt{m}\right) \Pi ^\perp$, using the quantization (2.22) for the 2-homogeneous symbol $\texttt{m}_2(z; \cdot )$ and (2.23) for the non-homogeneous symbol $\texttt{m}_{\ge 4}(z; \cdot )$, getting

5.13 $$\begin{aligned} \begin{aligned} \Pi ^\top \textrm{Op}^{\!\scriptscriptstyle BW}\!\left( \textrm{i}\, \texttt{m}(z; \cdot )\right) \Pi ^\perp z =&\!\!\!\!\!\! \sum _{ j_1-j_2 +j=k \atop j_1 \ne j_2, \ j \in \Lambda ^c, k \in \Lambda } \!\!\! \chi _2 \left( j_1, j_2,\frac{j+k}{2}\right) \textrm{i}\, \texttt{m}_{j_1, j_2}^{+,-}\left( \frac{j+k}{2}\right) z_{j_1} {\overline{z}}_{j_2} z_j \, {e^{\textrm{i}k x}} \\&+ \!\!\!\!\!\! \sum _{j \in \Lambda ^c, \, k \in \Lambda } \!\!\! \chi \left( k-j,\frac{j+k}{2} \right) \textrm{i}\, \hat{\texttt{m}}_{\ge 4}\left( z; k-j, \frac{k+j}{2}\right) \, z_j \, {e^{\textrm{i}k x}} \ . \end{aligned}\nonumber \\ \end{aligned}$$

Arguing as in (5.12), the first line of (5.13) vanishes. To deal with the second line, recall that also $\chi (\xi ', \xi ) \equiv 0$ when $|\xi '| \ge \langle \xi \rangle /10$, so when $k \in \Lambda$ and $j \in \Lambda ^c$ (so $|j-k|\ge 1$)

$$\begin{aligned} \frac{1}{10}{\langle \frac{j + k }{2} \rangle } = \frac{1}{10}\big (1+ \frac{|j \pm 1|}{2} \big ) \le \frac{3 + |j|}{20} \le \frac{4 + |j - k| }{20} \le \frac{ |j - k| }{4} \le |j-k| \ , \end{aligned}$$

proving that $\chi \left( k-j, \frac{j+k}{2}\right) \equiv 0$. In conclusion, also the second line of (5.13) vanishes, proving the first of (5.10). The second identity is analogous exchanging the roles of *j* and *k*.

Proof of (5.11). It follows writing $\Pi ^\perp = \textrm{Id}- \Pi ^\top$ and using the first of (5.10).

This concludes the analysis of the projection of the paradifferential vector field $\textrm{Op}^{\!\scriptscriptstyle BW}\!\left( \textrm{i}\, \texttt{m}\right) z$.

We pass to the cubic vector field $X^{(\Lambda )}(Z)$ in (4.9). We set

$$\begin{aligned} Y_3^{(\Lambda )}(z):= (\Pi _{{{\mathcal {P}}}^{(0)}_\Lambda } X^{(\Lambda )})(Z)^+ \ , \end{aligned}$$

which has the claimed form (5.5) in view of (4.10). Then we set that

$$\begin{aligned} Y_3^\top (z) := \Pi ^\top \left( X^{(\Lambda )}(Z)^+ - (\Pi _{{{\mathcal {P}}}^{(0)}_\Lambda } X^{(\Lambda )})(Z)^+ \right) \ , \quad Y_3^\perp (z) := \Pi ^\perp X^{(\Lambda )}(Z)^+ . \end{aligned}$$

To prove estimates (5.6) we exploit that $X^{(\Lambda )}(Z)$ is in strong-$\Lambda$ normal form, see (4.10).

$$\underline{\hbox {Estimate of }Y_3^\top (z).}$$ By definition,

$$\begin{aligned} Y_3^\top (z) = \sum _{k \in \Lambda } \ \ \sum _{({\jmath }, k, \mathbf {\sigma }, -) \in {{\mathcal {P}}}\setminus {{\mathcal {P}}}^{(0)}_\Lambda } X_{{\jmath }, k }^{\mathbf {\sigma }, +} \, z_{{\jmath } }^{\mathbf {\sigma }} \, e^{\textrm{i}k x} \ , \quad {\jmath } = (j_1, j_2, j_3) , \quad \mathbf {\sigma }=(\sigma _1, \sigma _2, \sigma _3) \ . \end{aligned}$$

By (4.10), $\Pi _{{\mathcal {P}}_\Lambda ^{(1)}} X^{(\Lambda )}= \Pi _{{\mathcal {P}}_\Lambda ^{(2)}} X^{(\Lambda )} = 0$, so, since $k \in \Lambda$, the only possibly remaining monomials are those with $({\jmath }, k, \mathbf {\sigma }, -) \in {{\mathcal {P}}}^{(3)}_\Lambda$ and in addition ${\jmath } \in (\Lambda ^c) ^3$. Then, recalling (4.9), $Y_3^\top (z) = \Pi ^\top \left( R^{(\Lambda )}(Z^\perp )Z^\perp \right) ^+$, $$Z^\perp :=\Big ({\begin{smallmatrix}z^\perp \\ {\overline{z}}^\perp \end{smallmatrix}}\Big )$$, and the first estimate (5.6) follows from $\Vert Y_3^\top (z) \Vert _{{\mathtt s}} \lesssim \Vert Y_3^\top (z) \Vert _{L^2}$ and estimate (2.36).

$\underline{\hbox {Estimate of } Y^\perp _3(z).}$ Again by (4.10), we expand $Y^\perp _3(z)$ as

$$\begin{aligned} Y_3^\perp (z) = \sum _{k \in \Lambda ^c} \ \ \sum _{({\jmath }, k, \mathbf {\sigma }, -) \in {{\mathcal {P}}}^{(3)}_\Lambda \cup {{\mathcal {P}}}^{(4)}_\Lambda } X_{{\jmath }, k }^{\mathbf {\sigma }, +} \, z_{{\jmath } }^{\mathbf {\sigma }} \, e^{\textrm{i}k x} \ . \end{aligned}$$

Then either (*i*) two indexes among $(j_1, j_2, j_3)$ belong to $\Lambda ^c$ and one to $\Lambda$, or (*ii*) all three indexes belong to $\Lambda ^c$. Consequently $Y_3^\perp (z) = \Pi ^\perp \left( R^{(\Lambda )}(Z^\perp )Z^\perp + R^{(\Lambda )}\right. \left. (Z^\perp )Z^\top + 2 R^{(\Lambda )}(Z^\perp , Z^\top )Z^\perp \right) ^+$, $$Z^\top :=\Big ({\begin{smallmatrix}z^\top \\ {\overline{z}}^\top \end{smallmatrix}}\Big )$$. The second estimate (5.6) follows again from estimate (2.36) (with $m \leadsto -4$), using also the trivial bound $\Vert z^\top \Vert _{{\mathtt s}} \le C_{{\mathtt s}, {\mathfrak {s}}_0} \Vert z^\top \Vert _{{\mathfrak {s}}_0}$. This concludes the analysis of the projection of $X^{(\Lambda )}(Z)$.

Finally we consider the projections of the vector field ${\widetilde{B}}_{\ge 4}(Z)Z$ in (4.7). We put

$$\begin{aligned}&Y^\top _{\ge 5}(z) := \Pi ^\top \big ({\widetilde{B}}_{\ge 4}(Z) Z\big )^+ + \Pi ^\top \textrm{Op}^{\!\scriptscriptstyle BW}\!\left( \textrm{i}\, \texttt{m}_{\ge 4} \right) \Pi ^\top z \ , \quad Y^\perp _{\ge 5}(z) := \Pi ^\perp \big ({\widetilde{B}}_{\ge 4}(Z) Z\big )^+ . \end{aligned}$$

$\underline{\hbox {Estimate of }Y^\perp _{\ge 5}(z).}$ This follows, since ${\widetilde{B}}_{\ge 4}(Z)$ is a matrix of non-homogeneous 0-operators in ${{\mathcal {M}}}^0_{\ge 4}[r]$, see (2.33).

$\underline{\hbox {Estimate of } Y^\top _{\ge 5}(z).}$ This proceeds as with in the previous one, using (2.30) and $\Vert \Pi ^\top z \Vert _{\mathtt s}\lesssim \Vert z \Vert _{{\mathtt s}-1}$ as well. $\square$

The next step is to extract an effective system driving the dynamics of particular solutions of (5.3)–(5.4) which we call *long-time controlled*, see Definition 5.2 below. These solutions have two main features: (*i*) the initial data is supported mostly on $\Lambda$ and (*ii*) they have a large a-priori bound on the high norm $\Vert \cdot \Vert _s$ for long times. These features allow us to propagate smallness of both tangential and normal modes in the low norm $\Vert \cdot \Vert _{{\mathfrak {s}}_0}$ for long times, and moreover to ensure that the normal modes keep having a size much smaller than the tangential ones, i.e. $\Vert z^\perp (t) \Vert _{{\mathfrak {s}}_0} \ll \Vert z^\top (t) \Vert _{L^2}$, see (5.17), (5.18). This is possible because of the normal form procedure of the previous section, and in particular because

(i) the leading term in the dynamics of the low modes $z^\top (t)$ in (5.3) is the cubic integrable vector field $Y_3^{(\Lambda )}(z^\top )$ (the non-explicit cubic term $Y^\top _3(z) = {{\mathcal {O}}}((z^\perp )^3)$, hence its size is much smaller);
(ii) in equation (5.4) for $z^\perp (t)$, the term $\textrm{Op}^{\!\scriptscriptstyle BW}\!\left( \textrm{i}\, \texttt{m}(z; x, \xi ) \right) z^\perp$ is skew-adjoint, hence it vanishes in a $L^2$-energy estimate; consequently the dominant term becomes $Y_3^\perp (z)$ which, in view of (5.6), fulfills the quadratic estimate $\Vert Y_3^\perp (z) \Vert _{{\mathfrak {s}}_0} \lesssim \Vert z^\top \Vert _{{\mathfrak {s}}_0} \Vert z^\perp \Vert _{{\mathfrak {s}}_0}^2$ and therefore has a very small size. To obtain such estimate is the reason why we put $X^{(\Lambda )}(Z)$ in (4.9) in strong-$\Lambda$ normal form, namely it does not contain monomials of the form $z_{j_1}^{\sigma _1} z_{j_2}^{\sigma _2} z_{j_3}^{\sigma _3} e^{\textrm{i}j x}$ supported in ${{\mathcal {P}}}^{(2)}_\Lambda$. Otherwise, $Y^\perp _3(z)$ would have had monomials with exactly two frequencies among $(j_1, j_2, j_3)$ in $\Lambda$ and one in $\Lambda ^c$, and the estimate in (5.6) would have had an additional term $\Vert z^\top \Vert _{{\mathfrak {s}}_0}^2 \Vert z^\perp \Vert _{s}$, which is too large for the bootstrap lemma 5.3 below.

We now introduce precisely, the notion of long-time controlled solutions.

### Definition 5.2

(**Long-time controlled solutions**) Let $s, \theta$ as in (5.2). Let also $T_\star >0$ and ${\epsilon }\in (0,{\mathfrak {r}})$. We say that a solution $z(t) \in H^s({{\mathbb {T}}}, {{\mathbb {C}}})$ of system (5.3)–(5.4) is *long-time controlled* with parameters $(s, \theta , T_\star , {\epsilon })$ if

- (*A*1) at time 0 fulfills
  5.14 $$\begin{aligned} \Vert z^\top (0, \cdot ) \Vert _{L^2} \le {\epsilon }\ , \qquad \Vert z^\perp (0, \cdot ) \Vert _{L^2} \le {\epsilon }^{3} \ ; \end{aligned}$$
- (*A*2) it exists over the time interval $[0, T_\star ]$ where it fulfills the large a-priori bound
  5.15 $$\begin{aligned} \sup _{0 \le t \le T_\star } \Vert z(t) \Vert _s \le {\epsilon }^{-\theta } \ . \end{aligned}$$

One crucial property of any long-time controlled solution is that its low norm $\Vert \cdot \Vert _{{\mathfrak {s}}_0}$ is automatically small for all $0 \le t \le T_\star$, as we shall now prove.

### Lemma 5.3

(Bootstrap lemma) Let $s, \theta$ as in (5.2). Also, fix that $T_0 >0$. There exists ${\epsilon }_\star = {\epsilon }_\star (\theta , T_0) >0$ such, that for any ${\epsilon }\in (0, {\epsilon }_\star )$, the following holds true.

Let *z*(*t*) be a solution of (5.3)–(5.4) which is long-time controlled with parameters $(s, \theta , T_\star , {\epsilon })$ (according to Definition 5.2) and with

5.16 $$\begin{aligned} T_\star \le \frac{T_0}{{\epsilon }^2}\log \left( \frac{1}{{\epsilon }} \right) \ . \end{aligned}$$

Then *z*(*t*) fulfills the improved $L^2$-bound

5.17 $$\begin{aligned} \Vert z^\top (t) \Vert _{L^2} \le 2 {\epsilon }\ , \qquad \Vert z^\perp (t) \Vert _{L^2} \le {\epsilon }^{3- \frac{ 3}{2}\theta } \ , \quad \forall 0\le t \le T_\star \end{aligned}$$

and the improved low-norm bound

5.18 $$\begin{aligned} \Vert z(t) \Vert _{{\mathfrak {s}}_0} \le 3 {\epsilon }\ , \qquad \Vert z^\perp (t) \Vert _{{\mathfrak {s}}_0} \le {\epsilon }^{2} \ \ , \quad \forall 0 \le t \le T_\star \ . \end{aligned}$$

### Proof

The proof is by a bootstrap argument. We assume the bound

5.19 $$\begin{aligned} \Vert z^\top (t) \Vert _{L^2} \le 10 {\epsilon }, \quad \Vert z^\perp (t) \Vert _{L^2} \le {\epsilon }^{3- 2 \theta } \ , \quad \forall 0 \le t \le T_\star , \end{aligned}$$

and show that, provided ${\epsilon }\in (0, {\epsilon }_\star )$ with ${\epsilon }_\star$ sufficiently small, the better bound (5.17) holds.

First we bound $\Vert z^\perp (t) \Vert _{{\mathfrak {s}}_0}$. This is done interpolating the bound on $\Vert z^\perp (t) \Vert _{L^2}$ that we have by the bootstrap assumption (5.19) and the large bound that we have on $\Vert z^\perp (t) \Vert _{s}$ in (5.15), being *z*(*t*) long-time controlled by assumption. We obtain

5.20 $$\begin{aligned} \Vert z^\perp (t) \Vert _{{\mathfrak {s}}_0}&\le \Vert z^\perp (t) \Vert _{L^2}^{1-\frac{{\mathfrak {s}}_0}{s}} \, \Vert z^\perp (t) \Vert _{s}^{\frac{{\mathfrak {s}}_0}{s}} {\mathop {\le }\limits ^{(5.19), (5.15) }} {\epsilon }^{3 - \theta (2-\frac{{\mathfrak {s}}_0}{s}) - 3 \frac{{\mathfrak {s}}_0}{s}} \le {\epsilon }^2 , \end{aligned}$$

which is possible for $s, \theta$ as in (5.2). Using the first part of (5.19) again, we also get

5.21 $$\begin{aligned} \Vert z(t) \Vert _{{\mathfrak {s}}_0} \le 11 {\epsilon }\ , \qquad \forall 0\le t \le T_\star \ . \end{aligned}$$

Next we consider $\Vert z^\top (t) \Vert _{L^2}$ and prove the improved estimate (5.17). Recall that the function $z^\top (t)$ fulfills equation (5.3); since $Y^{(\Lambda )}_3(z)$ is integrable, we get that, for all times $0 \le t \le T_\star$,

$$\begin{aligned} \frac{\textrm{d}}{\textrm{d}t} \Vert z^\top (t) \Vert _{L^2}^2&= 2\underbrace{ \textrm{Re }\langle -\textrm{i}|D|^\alpha z^\top +Y^{(\Lambda )}_3(z) , z^\top \rangle }_{ = 0} + 2 \textrm{Re }\langle Y^{\top }_3(z) + Y^\top _{\ge 5}(z), z^\top \rangle \\&{\mathop { \le }\limits ^{(5.6), (5.8)}} C \big ( \Vert z^\perp (t) \Vert _{{\mathfrak {s}}_0}^3 + \Vert z(t) \Vert _{{\mathfrak {s}}_0}^5 \big ) \, \Vert z^\top (t) \Vert _{L^2} {\mathop {\le }\limits ^{(5.20), (5.21), (5.19)}} C {\epsilon }^6 . \end{aligned}$$

Then, since *z*(*t*) is long-time controlled, its initial datum $z^\top (0)$ is bounded by (5.14); hence, for all times $0 \le t \le T_\star \le \frac{T_0}{{\epsilon }^2}\log \left( \frac{1}{{\epsilon }} \right)$,

5.22 $$\begin{aligned} \Vert z^\top (t) \Vert _{L^2}^2 \le \Vert z^\top (0) \Vert _{L^2}^2 + |t| C {\epsilon }^6 \le {\epsilon }^2 + C T_0 {\epsilon }^4 \log ({\epsilon }^{-1}) \le 4{\epsilon }^2 , \end{aligned}$$

provided that $0< {\epsilon }\le {\epsilon }_\star$ and that ${\epsilon }_\star$ is sufficiently small. This proves the first estimate in (5.17).

Next we bound $\Vert z^\perp (t) \Vert _{L^2}$. We exploit that the paradifferential operator in equation (5.4) is skew-adjoint, so we get, for all times $0 \le t \le T_\star \le \frac{T_0}{{\epsilon }^2}\log \left( \frac{1}{{\epsilon }} \right)$,

$$\begin{aligned} \frac{\textrm{d}}{\textrm{d}t} \Vert z^\perp (t) \Vert _{L^2}^2&= 2\underbrace{ \textrm{Re }\langle \Big (-\textrm{i}|D|^\alpha +\textrm{Op}^{\!\scriptscriptstyle BW}\!\left( \textrm{i}\texttt{m}(z;\cdot )\right) \Big ) z^\perp , z^\perp \rangle }_{ = 0}+ 2 \textrm{Re }\langle Y^\perp _3(z) + Y^\perp _{\ge 5}(z), z^\perp \rangle \\&{\mathop { \le }\limits ^{(5.6), (5.8)}} C \left( \Vert z(t) \Vert _{{\mathfrak {s}}_0} \, \Vert z^\perp (t) \Vert _{{\mathfrak {s}}_0}^2 + \Vert z(t) \Vert _{{\mathfrak {s}}_0}^5 \right) \Vert z^\perp (t) \Vert _0 \\&{\mathop {\le }\limits ^{(21), (20),(19)}} C {\epsilon }^{8- 2 \theta } \ . \end{aligned}$$

Again, that *z*(*t*) is long-time controlled, its initial datum $z^\perp (0)$ fulfills (5.14); hence, for all times $0 \le t \le T_\star \le \frac{T_0}{{\epsilon }^2}\log \left( \frac{1}{{\epsilon }} \right)$, we bound

5.23 $$\begin{aligned} \Vert z^\perp (t) \Vert _{L^2}^2 \le \Vert z^\perp (0) \Vert _{L^2}^2 + |t| C {\epsilon }^{8-2\theta } \le {\epsilon }^6 + C T_0 {\epsilon }^{6- 2\theta } \log ({\epsilon }^{-1}) \le {\epsilon }^{2(3-\frac{3}{2} \theta )} \ ,\nonumber \\ \end{aligned}$$

which is true shrinking ${\epsilon }_\star$. Estimates (5.22) and (5.23) prove (5.17). This verifies the bootstrap assumption and so, by (5.20), also the second of (5.18). Together with (5.17), we get also the first of (5.18). $\square$

A second important property of any long-time controlled solution is that it fulfills an *effective equation* with a very precise structure: up to higher order corrections, for long times, the modes $z_{\pm 1}(t)$ rotate with constant speed, whereas $z^\perp (t)$ fulfills a linear Schrödinger equation whose Hamiltonian $- \textrm{i}|D|^\alpha + \textrm{i}\textrm{Op}^{\!\scriptscriptstyle BW}\!\left( {\mathtt v}(x-{\mathtt J}_1 t) \xi \right)$ *does not have constant coefficients*. We shall show, in the next section, that this Hamiltonian is actually responsible for the growth of Sobolev norms of the solution. More precisely, we prove the following result:

### Proposition 5.4

Let $s, \theta$ as in (5.2). Fix also $T_0 >0$. There exists ${\epsilon }_\star = {\epsilon }_\star (s, \theta , T_0) >0$ such that for any ${\epsilon }\in (0, {\epsilon }_\star )$ the following holds true. Let *z*(*t*) be a solution of (5.3)–(5.4) which is long-time controlled with parameters $(s, \theta , T_\star , {\epsilon })$ (see Definition 5.2) and with $T_\star$ fulfilling (5.16). Then $z(t) = (z_{1}(t), z_{-1}(t), z^\perp (t))$ fulfills the system

5.24 $$\begin{aligned} {\left\{ \begin{array}{ll} \partial _t z_1 = - \textrm{i}\big ( 1+ |z_1(0)|^2\big ) z_1 + {\mathtt d}_1(t) \\ \partial _t z_{-1} = - \textrm{i}(1- |z_{-1}(0)|^2 \big ) z_{-1} + {\mathtt d}_{-1}(t) \\ \partial _t z^\perp = - \textrm{i}|D|^\alpha z^\perp + \textrm{i}\textrm{Op}^{\!\scriptscriptstyle BW}\!\left( \mathfrak {v}(x - {\mathtt J}_1 t) \xi + {\mathtt V}(t;x) \xi + {\mathtt b}(t;x, \xi )\right) z^\perp + Y(t), \end{array}\right. } \end{aligned}$$

where

$\bullet$ ${\mathtt J}_1$ is the real number

5.25 $$\begin{aligned} {\mathtt J}_1:= \frac{|z_1(0)|^2 + |z_{-1}(0)|^2}{2} \ , \end{aligned}$$

$\bullet$ the real valued function $\mathfrak {v}(x)$ is given by

5.26 $$\begin{aligned} \mathfrak {v}(x) := 2 \textrm{Re }\left( z_1(0) \, \overline{z_{-1}(0)} \, e^{\textrm{i}2 x} \right) \end{aligned}$$

whereas the real valued, time dependent function ${\mathtt V}(t;x)$ fulfills the estimate

5.27 $$\begin{aligned} \Vert {\mathtt V}(t; \cdot ) \Vert _{W^{2,\infty }} \le C {\epsilon }^{{4}- \theta } \ , \quad \forall 0 \le t \le T_\star \ ; \end{aligned}$$

$\bullet$ the real valued symbol ${\mathtt b}(t;x, \xi ) \in \Gamma ^\alpha _{W^{2,\infty }}$ fulfills the estimate (recall (2.13)): for every $n \in {{\mathbb {N}}}_0$, there is $C_n >0$ such that

5.28 $$\begin{aligned} \, \left| {\mathtt b}(t; \cdot ) \right| _{\alpha , W^{2, \infty }, n} \le C_n {\epsilon }^2 \ , \quad \forall 0 \le t \le T_\star \ ; \end{aligned}$$

$\bullet$ the functions ${\mathtt d}_{\pm 1}(t)$ fulfill the estimates

5.29 $$\begin{aligned} \left| {\mathtt d}_{\pm 1}(t) \right| \le {\epsilon }^{5-\theta } \ , \quad \forall 0 \le t \le T_\star \ ; \end{aligned}$$

$\bullet$ the vector field $Y(t) \equiv Y(t, x)$ fulfills the estimate

5.30 $$\begin{aligned} \Vert Y(t; \cdot ) \Vert _{s } \le C {\epsilon }^{3-\theta } \ , \quad \forall 0 \le t \le T_\star \ . \end{aligned}$$

### Proof

We shall use that *z*(*t*), being long-time controlled with parameters $(s, \theta , T_\star , {\epsilon })$ and with $T_\star$ fulfilling (5.16), satisfies the bounds (5.17), (5.18).

$\underline{\hbox {Equations for } z_{\pm 1}(t).}$ Write equation (5.3) in components, using the explicit expression of $Y_3^{(\Lambda )}$ in (5.5), to get the coupled system

5.31 $$\begin{aligned} {\left\{ \begin{array}{ll} \partial _t z_1 = - \textrm{i}z_1 - \textrm{i}|z_1|^2 z_1 + \langle Y_3^\top (z) + Y^\top _{\ge 5}(z), e^{\textrm{i}x} \rangle \\ \partial _t z_{-1} = - \textrm{i}z_{-1} + \textrm{i}|z_{-1}|^2 z_{-1} + \langle Y_3^\top (z) + Y^\top _{\ge 5}(z), e^{-\textrm{i}x} \rangle \ . \end{array}\right. } \end{aligned}$$

Consider the equation for $z_1$. We write this as

5.32 $$\begin{aligned} \begin{aligned} \partial _t z_1&= - \textrm{i}( 1+ |z_1(0)|^2) z_1 + {\mathtt d}_1(t) , \\&\quad {\mathtt d}_1(t) := -\textrm{i}\left( |z_1(t)|^2 - |z_1(0)|^2 \right) z_1(t) + \langle Y_3^\top (z) + Y^\top _{\ge 5}(z), e^{\textrm{i}x} \rangle , \end{aligned} \end{aligned}$$

giving the first equation in (5.24). We prove now that ${\mathtt d}_1(t)$ fulfills the bound claimed in (5.29). First, using the first of (5.31) and assumption (5.16), we get, for all times $0 \le t \le T_\star \le \frac{T_0}{{\epsilon }^2}\log \left( \frac{1}{{\epsilon }} \right)$,

$$\begin{aligned} \frac{\textrm{d}}{\textrm{d}t} |z_1(t)|^2&= 2 \textrm{Re }\left( \langle Y_3^\top (z)+ Y^\top _{\ge 5}(z), e^{\textrm{i}x} \rangle \, {\overline{z}}_1 \right) \\&{\mathop { \le }\limits ^{(5.6), (5.8)}} C \big ( \Vert z^\perp (t) \Vert _{{\mathfrak {s}}_0}^3 \!+\! \Vert z(t) \Vert _{{\mathfrak {s}}_0}^5 \big ) \Vert z^\top (t) \Vert _{0} {\mathop {\le }\limits ^{(5.18), (5.21), (5.17)}} C {\epsilon }^6 \ , \end{aligned}$$

which implies, on the same time scale,

5.33 $$\begin{aligned} \left| \big ( |z_1(t)|^2 - |z_1(0)|^2 \big ) \right| \le C |t| {\epsilon }^6 \le C T_0 \, {\epsilon }^4 \log ({\epsilon }^{-1}) \ . \end{aligned}$$

Hence we get that ${\mathtt d}_1(t)$ in (5.32) is bounded for $0 \le t \le T_\star \le \frac{T_0}{{\epsilon }^2}\log ({\epsilon }^{-1})$ by

5.34 $$\begin{aligned} \left| {\mathtt d}_1(t) \right|\le &   \left| \big ( |z_1(t)|^2 - |z_1(0)|^2 \big ) z_1(t) \right| + \left| \langle Y_3^\top (z)+ Y^\top _{\ge 5}(z), e^{\textrm{i}x} \rangle \right| \nonumber \\  &   {\mathop {\le }\limits ^{(5.33), (5.17)}} C T_0 \, {\epsilon }^5 \log ({\epsilon }^{-1}) + C {\epsilon }^5 \ , \end{aligned}$$

proving (5.29), provided that ${\epsilon }_\star$ is sufficiently small. An analogous argument proves that $z_{-1}(t)$ fulfills the second of (5.24).

A consequence, which we shall use in a moment, is that

5.35 $$\begin{aligned} z_{\pm 1}(t) = {\mathtt z}_{\pm 1}(t) + r_{\pm 1}(t) \ , \quad \text{ where } {\mathtt z}_{\pm 1}(t):= e^{- \textrm{i}t(1\pm |z_{\pm 1}(0)|^2)} z_{\pm 1}(0),\qquad \end{aligned}$$

whereas

$$\begin{aligned} r_{\pm 1} (t) := \int _0^t e^{- \textrm{i}(t - \tau ) (1 \pm |z_{\pm 1}(0)|^2)} \, {\mathtt d}_{\pm 1} (\tau ) \, \textrm{d}\tau \ \end{aligned}$$

fulfills, by (5.34), (5.16) and eventually shrinking ${\epsilon }_\star$ again, the bounds

5.36 $$\begin{aligned} |r_{\pm 1} (t)| \le {\epsilon }^{{3}-\theta } , \qquad \forall 0 \le t \le T_\star \ . \end{aligned}$$

$\underline{\hbox {Equation for }z^\perp (t).}$ We start from equation (5.4) and we substitute the explicit expression of $z_{\pm 1}(t)$ in (5.35). Consider first the symbol $\texttt{m}(z;x, \xi )$ in (5.7). We shall extract from its component $\langle \, \underline{{\mathtt V}}\, \rangle (Z; x)$, defined in (4.8), the main contribution which is the one supported on $z_{\pm 1}(t)$. More precisely,

$$\begin{aligned} \langle \, \underline{{\mathtt V}}\, \rangle (Z(t); x)&= 2\, \textrm{Re }\left( z_{1}(t) \, \overline{z_{-1}(t)} \, e^{\textrm{i}2 x} \right) + 2\, \textrm{Re }\Big ( \sum _{n \ge 2} z_{n}(t) \, \overline{z_{-n}(t)} \, e^{\textrm{i}2n x} \Big )\\&{\mathop {= }\limits ^{(5.35)}} \underbrace{ 2\, \textrm{Re }\left( z_{1}(0) \, \overline{z_{-1}(0)} \, e^{\textrm{i}2 x- 2{\mathtt J}_1 t} \right) }_{=\mathfrak {v}(x-{\mathtt J}_1 t) \text{ by } (5.26), (5.25)} \\  &+ \underbrace{2\, \textrm{Re }\left( \Big ( {\mathtt z}_{1}(t) \, \overline{r_{-1}(t)} + r_1(t) \overline{{\mathtt z}_{-1}(t)} + r_1(t) \overline{r_{-1}(t)} \Big ) \, e^{\textrm{i}2 x} \right) }_{=: {\mathtt V}_1(t; x)} \\&+\underbrace{ 2\, \textrm{Re }\Big ( \sum _{n \ge 2} z_{n}(t) \, \overline{z_{-n}(t)} \, e^{\textrm{i}2 n x} \Big )}_{=: {\mathtt V}_2(t; x)}\ . \end{aligned}$$

The functions ${\mathtt V}_1(t; x)$ and ${\mathtt V}_2(t;x)$ fulfill, by (5.14), (5.36) and (5.18), the bounds

5.37 $$\begin{aligned} \Vert {\mathtt V}_1(t; \cdot ) \Vert _{W^{2,\infty }} \le C {\epsilon }^{4-\theta } \ , \quad \Vert {\mathtt V}_2(t; \cdot ) \Vert _{W^{2,\infty }} \le C {\epsilon }^{4} \ , \quad \forall 0 \le t \le T_\star \ . \end{aligned}$$

Then we write $\texttt{m}(z;\cdot )$ in (5.7) as

$$\begin{aligned} \texttt{m}(z(t); x, \xi )= &   \mathfrak {v}(x-{\mathtt J}_1 t)\xi + \underbrace{({\mathtt V}_1(t; x) + {\mathtt V}_2(t; x) + {\widetilde{V}}_{\ge 4}(z(t); x) )}_{=: {\mathtt V}(t; x)}\xi \\    &   + \underbrace{ {a}^{(\alpha )}_2(z(t);x,\xi ) + {\widetilde{a}}^{(\alpha ) }_{\ge 4} (z(t);x,\xi ) }_{=: {\mathtt b}(t; x, \xi )} \end{aligned}$$

We bound ${\mathtt V}(t;x)$ using estimates (5.37) for ${\mathtt V}_1$ and ${\mathtt V}_2$, and that

$$\begin{aligned} \Vert {\widetilde{V}}_{\ge 4}(z(t); \cdot ) \Vert _{W^{2, \infty }} {\mathop {\le }\limits ^{(2.16)}} C \Vert z(t) \Vert _{{\mathfrak {s}}_0}^4 {\mathop {\le }\limits ^{(5.18)}} C {\epsilon }^4 \ , \quad \forall 0 \le t \le T_\star \ , \end{aligned}$$

getting the claimed bound (5.27).

The bound (5.28) for ${\mathtt b}(t;x, \xi )$ follows from (2.20), (2.16) and (5.18).

Finally we get that

$$\begin{aligned} Y(t, z):= Y_3^\perp (z(t)) + Y^\perp _{\ge 5}(z(t)), \end{aligned}$$

which fulfills the estimates (5.30) by (5.6), (5.8) and using (5.18) and (5.15). $\square$

## Instability via Paradifferential Mourre theory

The goal of this section is to give sufficient conditions on the initial datum *z*(0) ensuring that, if the corresponding solution *z*(*t*) is long-time controlled, then its high $H^s$-norm undergoes Sobolev norm explosion, becoming larger than ${\epsilon }^{-\theta }$. We will achieve this via a positive commutator estimate.

We will focus on the third equation in (5.24); actually it is more convenient to work with the translated variable

6.1 $$\begin{aligned} { \zeta (t,x):= z^\perp \big ( t, x +{\mathtt J}_1 t \big ) }\ , \quad {\mathtt J}_1 \text{ in } (5.25) \ . \end{aligned}$$

Clearly, one has

6.2 $$\begin{aligned} \Vert \zeta (t, \cdot ) \Vert _s = \Vert z^\perp (t, \cdot ) \Vert _s \ , \quad \forall t , \quad \forall s \in {{\mathbb {R}}}\ , \end{aligned}$$

so it is equivalent to prove growth of Sobolev norms for $\zeta (t)$ and $z^\perp (t)$. The equation fulfilled by $\zeta (t)$ is easily derived from the third of (5.24) as

6.3 $$\begin{aligned} \partial _t \zeta =&- \textrm{i}|D|^\alpha \zeta + \textrm{i}\textrm{Op}^{\!\scriptscriptstyle BW}\!\left( {({\mathtt J}_1+\mathfrak {v}(x ))} \xi \right) \zeta + \textrm{i}\textrm{Op}^{\!\scriptscriptstyle BW}\!\left( {\widetilde{{\mathtt V}}}(t; x) \xi + {\widetilde{{\mathtt b}}}(t; x, \xi )\right) \zeta + {\widetilde{Y}}(t) \end{aligned}$$

where we defined the real valued function ${\widetilde{{\mathtt V}}}(t; x)$, the real valued symbol ${\widetilde{{\mathtt b}}}(t; x, \xi )$ and the vector field ${\widetilde{Y}}(t; x)$ as

$$\begin{aligned} {\widetilde{{\mathtt V}}}(t; x) := {\mathtt V}(t, x+ {\mathtt J}_1 t) \ , \quad {\widetilde{{\mathtt b}}}(t; x, \xi ):= {\mathtt b}(t; x+ {\mathtt J}_1 t, \xi ) \ , \quad {\widetilde{Y}}(t; x) := Y(t; x+ {\mathtt J}_1 t) \ . \end{aligned}$$

It follows, by (5.27), (5.28) and (5.30), that we have the estimates

6.4 $$\begin{aligned}  &   \Vert {\widetilde{{\mathtt V}}}(t; \cdot ) \Vert _{W^{2,\infty }} \le C {\epsilon }^{{4}- \theta } \ , \quad \vert {\widetilde{{\mathtt b}}}(t; \cdot ) \vert _{\alpha , W^{2, \infty }, n} \le C_n {\epsilon }^2 \ , \nonumber \\    &   \quad \Vert {\widetilde{Y}}(t; \cdot ) \Vert _{s } \le C {\epsilon }^{3-\theta } \ , \quad \forall \ 0 \le t \le T_\star \ . \end{aligned}$$

### The Mourre operator

The leading term in equation (6.3) is the *non-constant coefficient transport* operator

6.5 $$\begin{aligned} \textrm{Op}^{\!\scriptscriptstyle BW}\!\left( \big ({\mathtt J}_1+\mathfrak {v}(x)\big ) \xi \right) \ , \quad {\mathtt J}_1 \text{ in } (5.25) \ , \ \ \ \mathfrak {v}(x) \text{ in } (5.26) \ . \end{aligned}$$

The crucial point is that, provided that $z_{1}(0)$ and $z_{-1}(0)$ fulfill

$$\begin{aligned} {\mathtt J}_1\equiv \frac{|z_1(0)|^2 + |z_{-1}(0)|^2}{2} < 2 |z_1(0) | \, |{z_{-1}(0)} | \ , \end{aligned}$$

corresponding to the function ${\mathtt J}_1 + \mathfrak {v}(x)$ having a zero, the operator $\textrm{Op}^{\!\scriptscriptstyle BW}\!\left( \big ({\mathtt J}_1+\mathfrak {v}(x)\big ) \xi \right)$ admits a Mourre-conjugate operator, namely an operator $\textsf{A}$ such that the commutator $\textrm{i}[\textsf{A}, \textrm{Op}^{\!\scriptscriptstyle BW}\!\left( \big ({\mathtt J}_1+\mathfrak {v}(x)\big ) \xi \right) ]$ is positive. Actually this also shows that the operator in (6.5) has a non-trivial absolutely continuous spectrum, although we shall not exploit directly this property.

More precisely, take *s* as in (5.2) and ${\mathtt R}\gg 1$ (to be fixed later) and define the (formally) self-adjoint operator

6.6 $$\begin{aligned} \begin{aligned} \textsf{A}:= \textsf{A}_{s,{\mathtt R}}&:= \textrm{Op}^{\!\scriptscriptstyle BW}\!\left( {\mathfrak {a}}(x, \xi )\right) , \quad {\mathfrak {a}}(x, \xi ):={\mathtt a}(x) \, |\xi | ^{2s} \, \eta ^2_{{\mathtt R}}(\xi ) \\&\text{ where } {\mathtt a}(x):= - \textrm{Im }\, \left( z_{1}(0) \, \overline{z_{-1}(0)} \, e^{\textrm{i}2 x }\right) \end{aligned} \end{aligned}$$

and $\eta _{\mathtt R}(\xi )$ the smooth step function

6.7 $$\begin{aligned} \eta _{\mathtt R}(\xi ):= \eta \left( \frac{\xi }{{\mathtt R}}\right) ,\quad \eta (y):= {\left\{ \begin{array}{ll} 0&  \text{ if } y\le 1\\ \dfrac{e^{-\frac{1}{y-1}}}{e^{-\frac{1}{y-1}} + e^{-\frac{1}{2-y}}}&  \text{ if } y \in (1,2) \\ 1&  \text{ if } y \ge 2 \end{array}\right. } \ . \end{aligned}$$

Note that ${\mathfrak {a}}(x, \xi )$ is a symbol in $\Gamma ^{2s}_{W^{2, \infty }}$, and for any $n \in {{\mathbb {N}}}_0$, there is $C_n >0$ such that

6.8 $$\begin{aligned} | {\mathfrak {a}}|_{2s , W^{2,\infty }, n } \le C_{s,n} \, |z_1(0)|\,|z_{-1}(0)| \ , \qquad | {\mathfrak {a}}|_{ 2s +1 , W^{2,\infty }, n } \le C_{s,n} \frac{ |z_1(0)|\,|z_{-1}(0)| }{{\mathtt R}} \ ,\nonumber \\ \end{aligned}$$

as it follows from its definition and from Lemma A.1 with $a \leadsto {\mathtt a}(x)|\xi |^{2s} \eta _{\mathtt R}(\xi )$, $m \leadsto 2s$, $N \leadsto 2$ and $\nu \leadsto 1$. Moreover we will ensure that $|z_1(0) z_{-1}(0)| >0$, so that $\textsf{A}$ is non trivial, see Remark 6.4.

The choice of the function ${\mathfrak {a}}(x,\xi )$ in (6.6) is motivated by the fact that it is an escape function for the symbol $({\mathtt J}_1 + \mathfrak {v}(x))\xi$ of the operator in (6.5); precisely one has the following result:

#### Lemma 6.1

Fix $s, {\mathtt R}>1$. Let ${\mathfrak {a}}(x,\xi )$ as in (6.6) and ${\mathtt J}_1$, $\mathfrak {v}(x)$ as in (5.25), (5.26). Then

6.9 $$\begin{aligned} \{ {\mathfrak {a}}(x,\xi ) , \, \big ({\mathtt J}_1 + \mathfrak {v}(x) \big )\xi \} = {\mathtt I}_1\, |\xi |^{2s} \, \eta _{\mathtt R}^2(\xi ) + a(x,\xi ) \end{aligned}$$

where ${\mathtt I}_1$ is the real number

6.10 $$\begin{aligned} {\mathtt I}_1:= 2 |z_1(0)| \, |z_{-1}(0)| \, \Big ( 2 |z_1(0)| \, |z_{-1}(0)| - \frac{|z_1(0)|^2 + |z_{-1}(0)|^2}{2} \Big ) \end{aligned}$$

whereas $a(x,\xi )$ is a smooth, non-negative symbol having the structure

6.11 $$\begin{aligned} a(x,\xi ) = a_1(x) \psi _1(\xi )^2 + a_2(x) \psi _2(\xi )^2 \ . \end{aligned}$$

Here $$ a_j(x)$$, $$j=1,2$$, are smooth, real valued, non-negative functions fulfilling

6.12 $$\begin{aligned} \Vert a_j(x) \Vert _{W^{3,\infty }} \le C \left( |z_1(0)|^4 + \, |z_{-1}(0)|^4 \right) \ , \end{aligned}$$

and $\psi _j(\xi )$, $j = 1,2$, are smooth, real valued symbols in ${\widetilde{\Gamma }}^s_0$ with support in $[{\mathtt R}, +\infty )$.

#### Proof

We compute, using (2.39), (6.6), (5.25), (5.26) and denoting $(\eta ')_{\mathtt R}(\xi ):= \eta '(\xi /{\mathtt R})$,

6.13 $$\begin{aligned}&\{ {\mathfrak {a}}(x, \xi ) , \big ({\mathtt J}_1 + \mathfrak {v}(x)\big )\xi \} = \left( 2 s\, {\mathtt a}\, \mathfrak {v}_x - \mathfrak {v}\, {\mathtt a}_x - {\mathtt J}_1 {\mathtt a}_x \right) |\xi |^{2s} \, \eta _{\mathtt R}^2 + \frac{2}{{\mathtt R}}{\mathtt a}\mathfrak {v}_x \, |\xi |^{2s}\xi \, \eta _{\mathtt R}\, (\eta ')_{\mathtt R}\nonumber \\&\quad = \left( {\mathtt a}\, \mathfrak {v}_x - \mathfrak {v}\, {\mathtt a}_x - {\mathtt J}_1 {\mathtt a}_x \right) |\xi |^{2s} \, \eta _{\mathtt R}^2 + (2s-1) {\mathtt a}\mathfrak {v}_x |\xi |^{2s} \eta _{\mathtt R}^2 + 2 {\mathtt a}\mathfrak {v}_x \, |\xi |^{2s}\eta _{\mathtt R}\, \frac{\xi }{{\mathtt R}} \, (\eta ')_{\mathtt R}\ . \end{aligned}$$

Now, using the explicit definition of ${\mathtt a}(x)$ in (6.6), of $\mathfrak {v}(x)$ in (5.26) and of ${\mathtt J}_1$ in (5.25) and that ${\mathtt a}_x(x)=- 2 \textrm{Re }\, \left( z_{1}(0) \, \overline{z_{-1}(0)} \, e^{\textrm{i}2 x }\right)$, $\mathfrak {v}_x(x)=-4 \textrm{Im }\big (z_1(0) \, \overline{z_{-1}(0)} e^{\textrm{i}2 x} \big )$, we get the lower bound

6.14 $$\begin{aligned} {\mathtt a}\mathfrak {v}_x - \mathfrak {v}{\mathtt a}_x - {\mathtt J}_1 {\mathtt a}_x&= 4\, \textrm{Im }\left( z_{1}(0) \, \overline{z_{-1}(0)} \, e^{\textrm{i}2 x } \right) ^2 + 4 \, \textrm{Re }\left( z_{1}(0) \, \overline{z_{-1}(0)} \, e^{\textrm{i}2 x } \right) ^2 - {\mathtt a}_x {\mathtt J}_1 \nonumber \\&\ge 4 \, | z_{1}(0)|^2 \, | z_{-1}(0)|^2 - 2 {\mathtt J}_1 |z_1(0)| \, |z_{-1}(0)| \nonumber \\&\ge 2 |z_1(0)| \, |z_{-1}(0)| \ \Big ( 2 |z_1(0)| \, |z_{-1}(0)| - {\mathtt J}_1 \Big ) \equiv {\mathtt I}_1 \ , \end{aligned}$$

where to pass from the first to the second line we also used that

$$\begin{aligned} |{\mathtt a}_x| \le 2 |z_1(0)| \, |z_{-1}(0)| \ . \end{aligned}$$

Hence, adding and subtracting ${\mathtt I}_1 |\xi |^{2s} \eta _{\mathtt R}^2(\xi )$ in (6.13), we get the claimed formula (6.9) with

$$\begin{aligned} a(x, \xi ) :=&\underbrace{\left( {\mathtt a}\mathfrak {v}_x - \mathfrak {v}{\mathtt a}_x - {\mathtt J}_1 {\mathtt a}_x - {\mathtt I}_1 +(2s-1) {\mathtt a}\mathfrak {v}_x \right) }_{=:a_1(x)} \underbrace{|\xi |^{2s} \eta _{\mathtt R}^2 }_{=:\psi _1(\xi )^2} + \underbrace{2 {\mathtt a}\mathfrak {v}_x }_{=:a_2(x)}\, \underbrace{|\xi |^{2s}\eta _{\mathtt R}\, \frac{\xi }{{\mathtt R}} \, (\eta ')_{\mathtt R}}_{=:\psi _2(\xi )^2} \ . \end{aligned}$$

Note that both $a_1(x)$ and $a_2(x)$ are non-negative functions in view of (6.14) and the fact that ${\mathtt a}\mathfrak {v}_x = 4\, \textrm{Im }\left( z_{1}(0) \, \overline{z_{-1}(0)} \, e^{\textrm{i}2 x } \right) ^2 \ge 0$. They clearly are smooth, and estimate (6.12) follows from the definitions of ${\mathtt a}(x), \mathfrak {v}(x)$ in (6.6), (5.26), of ${\mathtt J}_1$ in (5.25) and ${\mathtt I}_1$ in (6.10).

We claim that the functions $\psi _1(\xi )= |\xi |^s \eta _{\mathtt R}$ and $\psi _2(\xi ) = |\xi |^s \sqrt{\eta _{\mathtt R}\, \frac{\xi }{{\mathtt R}} (\eta ')_{\mathtt R}}$ are smooth symbols in ${\widetilde{\Gamma }}^{s}_0$ supported in $[{\mathtt R}, \infty )$. We prove the claim only for $\psi _2$ since the one for $\psi _1$ is trivial. First notice that $\psi _2$ is well defined since, by (6.7), one has $\xi (\eta ')_{\mathtt R}\ge 0$. Define that

$$\begin{aligned} f(y):= \sqrt{\eta (y) \, y \eta '(y)} \ , \quad \text {supp}(f) \subset [1,2] \ . \end{aligned}$$

Then $\psi _2(\xi )=|\xi |^s f(\xi /{\mathtt R})$ and is supported in $[{\mathtt R}, 2{\mathtt R}]$. Thus we are left to prove that *f*(*y*) is a smooth function. It is easy to see that $\sqrt{y \eta (y)}$ is smooth on its support. The function

$$\begin{aligned} \sqrt{\eta '(y)} = {\left\{ \begin{array}{ll} 0 \ , &  y\le 1\\ \dfrac{\sqrt{2 y^2-6 y+5}}{e^{-\frac{1}{2-y}}+e^{-\frac{1}{y-1}} } \cdot \dfrac{e^{-\frac{1}{2(y-1)}}}{y-1} \cdot \dfrac{e^{-\frac{1}{2(2-y)}}}{2-y} \ , &  y \in (1,2) \\ 0 \ ,&  y\ge 2 \end{array}\right. } \end{aligned}$$

is smooth by direct inspection. $\square$

Thanks to Lemma 6.1, we now prove that the commutator between $\textsf{A}$ in (6.6) and $\textrm{Op}^{\!\scriptscriptstyle BW}\!\left( ({\mathtt J}_1 + \mathfrak {v}(x))\xi \right)$ is a non-negative operator up to a small remainder. In the following, given two operators $\textsf{A}, \textsf{B}$, we write $\textsf{A}\ge \textsf{B}$ with the meaning $\langle \textsf{A}u, u \rangle \ge \langle \textsf{B}u, u \rangle$ for any $u \in \bigcap _s H^s$. More precisely, we have

#### Lemma 6.2

Fix $s, {\mathtt R}> 1$. Let $\textsf{A}\equiv \textsf{A}_{s, {\mathtt R}}$ be defined in (6.6). Then:

(i) **Positive commutator:** Let ${\mathtt J}_1$ in (5.25) and $\mathfrak {v}(x)$ in (5.26). One has
  6.15 $$\begin{aligned} \textrm{i}\big [\textsf{A}, \textrm{Op}^{\!\scriptscriptstyle BW}\!\left( \big ( {\mathtt J}_1 + \mathfrak {v}(x)\big ) \xi \right) \big ] \ge {\mathtt I}_1 \, \textrm{Op}^{\!\scriptscriptstyle BW}\!\left( |\xi |^{2 s} \eta ^2_{\mathtt R}(\xi )\right) + \textsf{R}\end{aligned}$$
  with ${\mathtt I}_1$ in (6.10) and the operator $\textsf{R}:H^{s} \rightarrow H^{-s}$ with estimate
  6.16 $$\begin{aligned} \Vert \textsf{R}u \Vert _{-s } \le C_{s} \frac{ |z_1(0)|^4 +|z_{-1}(0)|^4}{{\mathtt R}} \Vert u \Vert _s \ . \end{aligned}$$
(ii) **Upper bound:** One has
  6.17 $$\begin{aligned} \textsf{A}\le 2 |z_1(0)|\,|z_{-1}(0)|\, \, \textrm{Op}^{\!\scriptscriptstyle BW}\!\left( |\xi |^{2s} \eta _{\mathtt R}^2(\xi )\right) + \textsf{R}\end{aligned}$$
  with $\textsf{R}:H^{s} \rightarrow H^{-s}$ satisfying the estimate
  6.18 $$\begin{aligned} \Vert \textsf{R}u \Vert _{-s } \le C_{s} \frac{ |z_1(0)|^2 + |z_{-1}(0)|^2}{{\mathtt R}^2} \Vert u \Vert _s \ . \end{aligned}$$

#### Proof

(*i*) First note that $\big ({\mathtt J}_1 +\mathfrak {v}(x)\big ) \xi$ is a symbol in $\Gamma ^1_{W^{2, \infty }}$ with seminorm

6.19 $$\begin{aligned} | \big ( {\mathtt J}_1 + \mathfrak {v}(x)\big ) \xi |_{1, W^{2, \infty }, 7} \le C \left( |z_1(0)|^2 + |z_{-1}(0)|^2 \right) \ . \end{aligned}$$

We now compute the commutator between $\textsf{A}$ and $\textrm{Op}^{\!\scriptscriptstyle BW}\!\left( \big ( {\mathtt J}_1+ \mathfrak {v}(x)\big ) \xi \right)$. We use the composition Theorem 2.8 (*i*) regarding ${\mathfrak {a}}(x, \xi )$ as a symbol in $\Gamma ^{2s+1}_{W^{2, \infty }}$ (so putting $m \leadsto 2s+1$, $m' \leadsto 1$, $\varrho \leadsto 2$), and we get

6.20 $$\begin{aligned} \textrm{i}[\textsf{A}, \textrm{Op}^{\!\scriptscriptstyle BW}\!\left( \big ( {\mathtt J}_1 + \mathfrak {v}(x) \big ) \xi \right) ] = \textrm{Op}^{\!\scriptscriptstyle BW}\!\left( \{ {\mathfrak {a}}(x, \xi ) \, , \big ( {\mathtt J}_1+ \mathfrak {v}(x)\big ) \xi \}\right) + \breve{R} , \end{aligned}$$

where the operator $\breve{R} :H^{s} \rightarrow H^{ -s}$ satisfies

$$\begin{aligned} \Vert \breve{R} u \Vert _{-s}&\lesssim | {\mathfrak {a}}|_{2s +1, W^{2,\infty }, 7} \, | \big ( {\mathtt J}_1 + \mathfrak {v}(x) \big ) \xi |_{1, W^{2,\infty }, 7} \,\\&\Vert u \Vert _{s} {\mathop {\lesssim }\limits ^{(6.8), (6.19)}} \frac{ |z_1(0)|^4+ |z_{-1}(0)|^4}{{\mathtt R}} \Vert u \Vert _{s} \ . \end{aligned}$$

Back to formula (6.20), as the Poisson bracket $\{ {\mathfrak {a}}(x, \xi ) \,, \big ( {\mathtt J}_1+ \mathfrak {v}(x)\big ) \xi \}$ was already computed in (6.9), we have that

6.21 $$\begin{aligned} \textrm{Op}^{\!\scriptscriptstyle BW}\!\left( \{ {\mathfrak {a}}(x, \xi )\, , \big ({\mathtt J}_1 + \mathfrak {v}(x) \big ) \xi \}\right) = {\mathtt I}_1 \textrm{Op}^{\!\scriptscriptstyle BW}\!\left( |\xi |^{2{\mathtt s}} \eta ^2_{\mathtt R}\right) + \textrm{Op}^{\!\scriptscriptstyle BW}\!\left( a(x,\xi )\right) ,\nonumber \\ \end{aligned}$$

with $a(x, \xi )$ a smooth, non-negative symbol having the structure (6.11). Thanks to these properties we bound the operator $\textrm{Op}^{\!\scriptscriptstyle BW}\!\left( a\right)$ from below using the strong Garding inequality A.2, getting

6.22 $$\begin{aligned} \langle \textrm{Op}^{\!\scriptscriptstyle BW}\!\left( a\right) u, u \rangle\ge &   - C \frac{\Vert a_1 \Vert _{W^{3,\infty }} + \Vert a_2 \Vert _{W^{3,\infty }}}{{\mathtt R}^2} \Vert u \Vert _s^2\nonumber \\  &   {\mathop {\ge }\limits ^{(6.12) }} -C \frac{|z_1(0)|^4 + |z_{-1}(0)|^4}{{\mathtt R}^2} \langle \langle D\rangle ^{2s} u, u \rangle \ . \end{aligned}$$

We conclude by (6.20), (6.21), (6.22) that

$$\begin{aligned} \textrm{i}[\textsf{A}, \textrm{Op}^{\!\scriptscriptstyle BW}\!\left( \big ( {\mathtt J}_1 + \mathfrak {v}(x) \big ) \xi \right) ]\ge &   {\mathtt I}_1 \, \textrm{Op}^{\!\scriptscriptstyle BW}\!\left( |\xi |^{2{\mathtt s}} \eta ^2_{\mathtt R}\right) + \textsf{R}\ , \quad \\ \textsf{R}:= &   \breve{R} -C \frac{|z_1(0)|^4 + |z_{-1}(0)|^4}{{\mathtt R}^2}\langle D\rangle ^{2s}, \end{aligned}$$

where the operator $\textsf{R}:H^{s} \rightarrow H^{-s }$ fulfills the estimate (6.16).

(*ii*) Define the positive symbol ${\tilde{a}}(x,\xi ):= \left( 2 |z_1(0)|\,|z_{-1}(0)| -{\mathtt a}(x)\right) |\xi |^{2s} \eta _{\mathtt R}^2(\xi )$ and again apply Garding’s inequality A.2. $\square$

### Growth of Sobolev norms

We now give sufficient conditions on the initial data of a long-time controlled solution *z*(*t*) ensuring growth of Sobolev norms.

#### Definition 6.3

(**Well-prepared data**) Fix $s, \theta$ as in (5.2). Fix also $\nu _0\in (0, \frac{1}{2})$, ${\epsilon }>0$.

We say that an initial datum $z(0) \in H^s({{\mathbb {T}}}, {{\mathbb {C}}})$ is *well prepared* with parameters $(s, \theta , \nu _0, {\epsilon })$ if

1. On the modes on $\Lambda$
  6.23 $$\begin{aligned} 2 |z_1(0) | \, |{z_{-1}(0)} | - \frac{|z_1(0)|^2 + |z_{-1}(0)|^2}{2} \ge \nu _0 {\epsilon }^2 \ ; \end{aligned}$$
2. On the modes on $\Lambda ^c$
  6.24 $$\begin{aligned} \langle \textsf{A}_{s,{\mathtt R}} z^\perp (0), z^\perp (0) \rangle > {\epsilon }^{3-3\theta } \ , \quad \text{ with } {\mathtt R}:= {\epsilon }^{-(3+\theta )/(1-\alpha )} \ \end{aligned}$$
  and $$\textsf{A}_{s,{\mathtt R}}$$ in (6.6).

#### Remark 6.4

Condition (6.23) ensures that $|z_1(0) z_{-1}(0)| >0$, hence both $\mathfrak {v}(x)$ in (5.26) and the symbol ${\mathfrak {a}}(x,\xi )$ in (6.6) are non-trivial.

The next result proves that a solution *z*(*t*) which is long-time controlled for times ${T_0}{{\epsilon }^{-2}} \log \left( {{\epsilon }^{-1}} \right)$ with $T_0$ sufficiently large and whose initial datum is well-prepared, undergoes growth of Sobolev norms. Precisely:

#### Proposition 6.5

Fix $s, \theta$ as in (5.2). Fix also $\nu _0\in (0, \frac{1}{2})$. There exists ${\epsilon }_1 = {\epsilon }_1(s, \theta , \nu _0)>0$ such that for any ${\epsilon }\in (0, {\epsilon }_1)$, the following holds true. Let $z(t)\in H^s({{\mathbb {T}}}, {{\mathbb {C}}})$ be a solution of system (5.3)–(5.4) such that

(i) it is long-time controlled with parameters $(s, \theta , T_\star , {\epsilon })$ (see Definition 5.2), with
  6.25 $$\begin{aligned} T_\star = \frac{T_0}{{\epsilon }^2} \log \left( \frac{1}{{\epsilon }} \right) \ , \quad T_0 := \frac{1}{\nu _0} \ ; \end{aligned}$$
(ii) its initial datum $z(0)\in H^s({{\mathbb {T}}}, {{\mathbb {C}}})$ is well-prepared with parameters $(s, \theta , \nu _0, {\epsilon })$ (see Definition 6.3).

Then the solution *z*(*t*) undergoes growth of Sobolev norms, i.e.

6.26 $$\begin{aligned} \sup _{|t | \le T_\star } \Vert z(t) \Vert _s \ge \frac{1}{ {\epsilon }^{\theta }} \ . \end{aligned}$$

The first step to prove such result is to define the ${{\mathcal {A}}}$-functional

6.27 $$\begin{aligned} {{\mathcal {A}}}(t):= \langle \textsf{A}_{s,{\mathtt R}} \, \zeta (t), \zeta (t) \rangle , \quad \textsf{A}_{s,{\mathtt R}} \text{ in } (6.6) \ ,\quad \zeta (t) \text{ in } (6.1) \end{aligned}$$

and exploit Lemma 6.2 to give a *lower bound* on the time derivative $\frac{\textrm{d}}{\textrm{d}t} {{\mathcal {A}}}(t)$. More precisely, we have

#### Lemma 6.6

Under the same assumptions of Propositon 6.5, there are a constant $C>0$ and ${\epsilon }_1={\epsilon }_1(s,\theta ,\alpha ,\nu _0)>0$ such that if ${\epsilon }\in \mathrm{(}0,{\epsilon }_1)$ the ${{\mathcal {A}}}$- functional in (6.27), with ${\mathtt R}$ in (6.24) fulfills: then

6.28 $$\begin{aligned} \frac{\textrm{d}}{\textrm{d}t} {{\mathcal {A}}}(t)\ge&\, {{\epsilon }^2 \nu _0} \left( {{\mathcal {A}}}(t) - C {\epsilon }^{3-2\theta } \right) \ , \qquad \forall 0\le t \le \frac{T_0}{{\epsilon }^2}\log \left( \frac{1}{{\epsilon }} \right) \ . \end{aligned}$$

#### Proof

First note that if *z*(*t*) is a long-time controlled solution with parameters $(s, \theta , T_\star , {\epsilon })$ and has initial datum well prepared with parameters $(s, \theta , \nu _0, {\epsilon })$ then the translated solution $\zeta (t)$ defined in (6.1) is long-time controlled and has initial data well-prepared with the same parameters.

From now on we shall simply denote $\textsf{A}\equiv \textsf{A}_{s,{\mathtt R}}$. Since $\zeta (t)$ fulfills (6.3), we compute that

6.29 $$\begin{aligned} \frac{\textrm{d}}{\textrm{d}t} {{\mathcal {A}}}(t) =&\langle \textrm{i}[ \textsf{A}, \textrm{Op}^{\!\scriptscriptstyle BW}\!\left( \big ( {\mathtt J}_1 + \mathfrak {v}(x) \big ) \xi \right) ] \zeta , \zeta \rangle \end{aligned}$$

6.30 $$\begin{aligned}&+ \langle \textrm{i}[ \textsf{A},\textrm{Op}^{\!\scriptscriptstyle BW}\!\left( {\widetilde{{\mathtt V}}}(t;x) \xi \right) ] \zeta , \zeta \rangle \end{aligned}$$

6.31 $$\begin{aligned}&+ \langle \textrm{i}[ \textsf{A},\textrm{Op}^{\!\scriptscriptstyle BW}\!\left( - |\xi |^\alpha + {\widetilde{{\mathtt b}}}(t; x, \xi )\right) ] \zeta , \zeta \rangle \end{aligned}$$

6.32 $$\begin{aligned}&+ 2\textrm{Re }\, \langle \textsf{A}{\widetilde{Y}}(t) , \zeta \rangle \end{aligned}$$

We shall use that, for well-prepared data, the number ${\mathtt I}_1$ in (6.10) fulfills (see (6.23))

6.33 $$\begin{aligned} {\mathtt I}_1 \ge 2 | z_1(0)|\, | z_{-1}(0)| \nu _0 \, {\epsilon }^2, \end{aligned}$$

whereas, for long-time controlled solutions (see (5.14)), one has

6.34 $$\begin{aligned} |z_1(0)|^2+ |z_{-1}(0)|^2\le {\epsilon }^2. \end{aligned}$$

We first estimate the term (6.29) from below using Lemma 6.2. More precisely, we get

6.35 $$\begin{aligned} \langle \textrm{i}[ \textsf{A}, \textrm{Op}^{\!\scriptscriptstyle BW}\!\left( ({\mathtt J}_1+\mathfrak {v}(x)) \xi \right) ] \zeta ,&\zeta \rangle {\mathop {\ge }\limits ^{(6.15),(6.16)}} {\mathtt I}_1 \, \, \langle \textrm{Op}^{\!\scriptscriptstyle BW}\!\left( |\xi |^{2 s} \eta ^2_{\mathtt R}(\xi )\right) \zeta , \zeta \rangle - C_s \frac{{\epsilon }^{4}}{{\mathtt R}} \Vert \zeta \Vert _s^2\nonumber \\&{\mathop {\ge }\limits ^{(6.33)}} 2 | z_1(0)|\, | z_{-1}(0)| \nu _0 \, {\epsilon }^2 \, \langle \textrm{Op}^{\!\scriptscriptstyle BW}\!\left( |\xi |^{2 s} \eta ^2_{\mathtt R}(\xi )\right) \zeta , \zeta \rangle - C_s \frac{{\epsilon }^{4}}{{\mathtt R}} \Vert \zeta \Vert _s^2 \nonumber \\&{\mathop { \ge }\limits ^{(6.17), (6.18)}} {\nu _0} \, {\epsilon }^2 {{\mathcal {A}}}(t) - C_s \frac{{\epsilon }^{4}}{{\mathtt R}}\Vert \zeta \Vert _s^2 \ . \end{aligned}$$

Next we estimate (6.30) from above. We first use estimate (A.2) (with $\nu =0$, $m' = 1$, $m = 2s$),

6.36 $$\begin{aligned} \left| (6.30) \right| \le | {\mathfrak {a}}|_{2s, W^{2,\infty }, 7} \, |{\widetilde{{\mathtt V}}}(t,\cdot ) |_{1, W^{2,\infty }, 7} \, \Vert \zeta \Vert _s^2 {\mathop {\le }\limits ^{(6.8), (6.4), (6.34)}} C_s {\epsilon }^{6-\theta }\Vert \zeta \Vert _s^2 \ .\nonumber \\ \end{aligned}$$

Next we estimate (6.31) from above. We use again estimate (A.2) (this time with $\nu = 1-\alpha$, $m' = \alpha$, $m = 2s$, thinking ${\mathfrak {a}}(x,\xi )$ as a symbol in $\Gamma ^{2s + 1-\alpha }_{W^{2, \infty }}$ supported on high frequencies) to bound

6.37 $$\begin{aligned} \left| (6.31) \right| \le \frac{1}{{\mathtt R}^{1-\alpha }} \, | {\mathfrak {a}}|_{2s, W^{2,\infty }, 7} \ | |\xi |^\alpha + {\widetilde{{\mathtt b}}}(t, \cdot ) |_{\alpha , W^{2,\infty }, 7} \, \Vert \zeta \Vert _s^2 {\mathop {\le }\limits ^{(6.8), (6.4) }} C_s \frac{{\epsilon }^{2}}{{\mathtt R}^{1-\alpha }}\Vert \zeta \Vert _s^2 \ .\nonumber \\ \end{aligned}$$

Finally we estimate (6.32) from above. We use estimate (2.28) to bound

6.38 $$\begin{aligned} \left| (6.32) \right|&\le \Vert \textsf{A}{\widetilde{Y}}(t) \Vert _{-s} \Vert \zeta \Vert _{s} {\le } C_s | {\mathfrak {a}}|_{2s, L^{\infty }, 7} \Vert {\widetilde{Y}}(t) \Vert _{s}\Vert \zeta \Vert _{s} {\mathop {\le }\limits ^{(6.8), (6.4)}} C_s {\epsilon }^{5-\theta } \Vert \zeta \Vert _{s} \ . \end{aligned}$$

Then (6.28) follows from (6.35), (6.36), (6.37) and (6.38), choosing ${\mathtt R}$ as in (6.24), and using that $\zeta (t)$, being long-time controlled, fulfills $\Vert \zeta (t) \Vert _s \le {\epsilon }^{-\theta }$ and provided ${\epsilon }$ is sufficiently small. $\square$

We are finally able to prove Proposition 6.5.

#### Proof of Proposition 6.5

Let $z(t)\in H^s({{\mathbb {T}}}, {{\mathbb {C}}})$ be a solution of system (5.3)–(5.4) whose initial datum $z(0)\in H^s({{\mathbb {T}}};{{\mathbb {C}}})$ is well-prepared with parameters $(s, \theta , \nu _0, {\epsilon })$ and which is long-time controlled with parameters $(s, \theta , T_\star , {\epsilon })$, $T_\star$ in (6.25). By Lemma 6.6, provided ${\epsilon }>0$ is sufficiently small, the functional ${{\mathcal {A}}}(t)$ in (6.27) fulfills the inequality (6.28). Integrating in time, we get

6.39 $$\begin{aligned} {{\mathcal {A}}}(t) \ge e^{ \nu _0 {\epsilon }^2 t} \, \left( {{\mathcal {A}}}(0) - C {\epsilon }^{3-2\theta } \right) + C {\epsilon }^{3-{2\theta }} \ , \qquad 0\le t \le \frac{T_0}{{\epsilon }^2}\log \left( \frac{1}{{\epsilon }} \right) \ .\qquad \end{aligned}$$

A sufficient condition for ${{\mathcal {A}}}(t)$ to grow in time is that ${{\mathcal {A}}}(0) > C {\epsilon }^{3-2\theta }$; this condition is fulfilled for well-prepared initial data provided ${\epsilon }$ is sufficiently small; indeed by (6.24)

$$\begin{aligned}{{\mathcal {A}}}(0) = \langle \textsf{A}\zeta (0), \zeta (0) \rangle = \langle \textsf{A}z^\perp (0), z^\perp (0) \rangle> {\epsilon }^{3-3 \theta } > 2 C {\epsilon }^{3-2\theta } \ . \end{aligned}$$

Then, using also the penultimate of the above inequalities, ${{\mathcal {A}}}(0) - C {\epsilon }^{3-2\theta }>{\epsilon }^{3-3 \theta }- C {\epsilon }^{3-2\theta } > \frac{1}{2}{\epsilon }^{3-3\theta }$, and we get from (6.39), the definition (6.27) and the continuity Theorem 2.4

$$\begin{aligned} \begin{aligned} \frac{1}{2} {\epsilon }^{3-3\theta } \, e^{{\nu _0}{\epsilon }^2 t}&\le {{\mathcal {A}}}(t) \le \Vert \textsf{A}_{s,{\mathtt R}}\zeta (t) \Vert _{-s} \Vert \zeta (t) \Vert _s {\mathop {\le }\limits ^{(6.6), (6.8)}} C_s {\epsilon }^2\\&\Vert \zeta (t) \Vert _s^2 {\mathop {\le }\limits ^{(6.2)}} C_s {\epsilon }^2 \Vert z(t) \Vert _s^2 \end{aligned} \end{aligned}$$

for some $C_s >1$. Hence, when $t = \frac{T_0}{{\epsilon }^2} \log \left( \frac{1}{{\epsilon }}\right)$, eventually shrinking ${\epsilon }$, one gets

$$\begin{aligned} \Vert z(t) \Vert _s^2 \ge \frac{1}{2 C_s} {\epsilon }^{1-3\theta } \, e^{ \nu _0 T_0 \log ({\epsilon }^{-1})} {\mathop {\ge }\limits ^{(6.25)}} \frac{1}{{\epsilon }^{2\theta }}, \end{aligned}$$

yielding (6.26). $$\square $$

### Conclusion and proof of Theorem 1.1

Fix $s, \theta$ as in (5.2). We give now an example of a well-prepared initial data.

#### Lemma 6.7

Let $\rho _1, \rho _{-1}>0$ in the non-empty region limited by

6.40 $$\begin{aligned} \rho _1^2 + \rho _{-1}^2 \le 1 \ , \qquad \nu _0 := 2 \rho _1 \rho _{-1} - \frac{\rho _1^2 + \rho _{-1}^2}{2} > 0 \ . \end{aligned}$$

There exists ${\epsilon }_0 >0$ and, for any ${\epsilon }\in (0, {\epsilon }_0)$, an interval $I({\epsilon })$ such that the initial datum

6.41 $$\begin{aligned} z(0):= {\epsilon }\rho _1 e^{\textrm{i}x } + {\epsilon }\rho _{-1} e^{-\textrm{i}x} + \rho \, e^{\textrm{i}3{\mathtt N}x} + \textrm{i}\rho \, e^{\textrm{i}(3{\mathtt N}+ 2) x} \ , \quad {\mathtt N}:= \lceil {\mathtt R}\rceil \end{aligned}$$

with ${\mathtt R}= {\epsilon }^{-(3+\theta )/(1-\alpha )}$ and $\rho \in I({\epsilon })$, fulfills:

- **well-prepared:** *z*(0) in (6.41) is a well-prepared initial datum with parameters $(s, \theta , \nu _0, {\epsilon })$ (according to Definition  6.3);
- $L^2$-**smallness:** the bounds in (5.14) holds true;
- $H^s$-**smallness:** *z*(0) fulfills the high norm bound
  6.42 $$\begin{aligned} \Vert z(0) \Vert _s \le {\epsilon }^\theta \ . \end{aligned}$$

#### Proof

We first prove that each of the three claimed properties gives a restriction on the choice of $\rho$. Then we prove that such conditions are compatible.

$\underline{\mathbf{Well - prepared}}$: Condition (B1) follows immediately from (6.40). We now check condition (B2). Using the definition of paradifferential operator in (2.22), the form of $\textsf{A}$ in (6.6) and of *z*(0) in (6.41), we get

$$\begin{aligned} \langle \textsf{A}\Pi ^\perp z(0), \Pi ^\perp z(0) \rangle&= \sum _{k } {{\epsilon }^2 \rho _{1} \rho _{-1}} \, \left| k+1 \right| ^{2s} \, \eta _{\mathtt R}^2\big (k+1\big ) \, \chi _2 \big (1, - 1, k+1 \big ) \, \textrm{Im }\big ({\overline{z}}_{k}^\perp (0) \, z_{k+2}^\perp (0)\big ) \\&= {{\epsilon }^2 \rho _{1} \rho _{-1}} \, \left| 3{\mathtt N}+1 \right| ^{2s} \, \underbrace{\eta _{\mathtt R}^2\big (3{\mathtt N}+1\big )}_{=1} \, \underbrace{\chi _2 \big (1, -1, 3{\mathtt N}+1 \big )}_{=1} \, \rho ^2 = {\epsilon }^2 \rho _{1} \rho _{-1} \left| 3{\mathtt N}+1 \right| ^{2s} \, \rho ^2 \ . \end{aligned}$$

Then (6.24) is fulfilled provided $\rho _{1} \rho _{-1} 3^{2s} {\mathtt R}^{2s} \rho ^2 \ge {\epsilon }^{1-3\theta }$, which using (6.24) gives

6.43 $$\begin{aligned} \rho \ge \, \frac{{\epsilon }^{\frac{1}{2}-\frac{3}{2} \theta + s\frac{3+\theta }{1-\alpha }}}{3^s \sqrt{\rho _{1} \rho _{-1}}} . \end{aligned}$$

This proves that *z*(0) is well prepared.

$\underline{L^2-\textbf{smallness}}$: The first condition in (5.14) is satisfied thanks to the first assumption in (6.40) and the second condition in (5.14) is satisfied provided that

6.44 $$\begin{aligned} \rho \le \frac{{\epsilon }^3}{\sqrt{2}}. \end{aligned}$$

$\underline{H^s -\textbf{smallness}}$: The condition (6.42) is satisfied provided that

$$\begin{aligned}(\rho _1^2+\rho _{-1}^2){\epsilon }^2\le \frac{{\epsilon }^{2\theta }}{2} \quad \text {and} \quad \rho ^2 (3{\mathtt N}+1)^{2s}+ \rho ^2 (3{\mathtt N}+3)^{2s}\le \frac{{\epsilon }^{2\theta }}{2}.\end{aligned}$$

The first condition follows automatically from (6.40) and taking ${\epsilon }$ sufficiently small, while the second one, using ${\mathtt N}\le {\mathtt R}+1$ and (6.24), is fulfilled for example for

6.45 $$\begin{aligned} \rho \le \frac{{\epsilon }^{\theta +s\frac{3+\theta }{1-\alpha }}}{6^{s} 2 }. \end{aligned}$$

Note also that, since $s\ge 3{\mathfrak {s}}_0 \ge 1$, for ${\epsilon }$ small enough the second condition (6.44) is less restrictive than the third one (6.45). Note that, provided ${\epsilon }$ is small enough and using $\theta <\frac{1}{5}$, conditions (6.43) and (6.45) are compatible. Then, taking

$$\begin{aligned} \rho \in I({\epsilon }):=\Big ( \frac{1}{3^s \sqrt{\rho _{1} \rho _{-1}}} {\epsilon }^{\frac{1}{2}-\frac{3}{2} \theta + s\frac{3+\theta }{1-\alpha } } ,\, \frac{{\epsilon }^{\theta +s\frac{3+\theta }{1-\alpha }}}{6^{s} 2 } \Big ), \end{aligned}$$

the datum *z*(0) satisfies all the claimed conditions. $\square$

We now show that any solution of system (4.7) with a well prepared initial datum as in Lemma 6.7 undergoes Sobolev norm explosion. More precisely we have

#### Lemma 6.8

Fix $s, \theta$ as in (5.2). There exists ${\epsilon }_2 >0$ such that, provided ${\epsilon }\in (0, {\epsilon }_2)$ the following holds true. Let $z(0) \in H^s({{\mathbb {T}}}, {{\mathbb {C}}})$ as in Lemma 6.7 and so well-prepared with parameters $(s, \theta , \nu _0, {\epsilon })$, for some $\nu _0 \in (0, \tfrac{1}{2})$. Consider the solution *z*(*t*) of system (5.3)–(5.4) with initial datum *z*(0). Denote that

6.46 $$\begin{aligned} 0<T_1:= T_1({\epsilon }; z(0)) := \inf \left\{ t \ge 0 :\ \ \Vert z(t) \Vert _s \ge {\epsilon }^{-\theta } \right\} \ . \end{aligned}$$

Then $T_1$ is finite and bounded by $T_1 \le \frac{T_0}{{\epsilon }^2}\log \left( \frac{1}{{\epsilon }} \right)$, $T_0 = \nu _0^{-1}$. Moreover one has that

6.47 $$\begin{aligned} \sup _{0 \le t \le T_1} \Vert z(t) \Vert _{{\mathfrak {s}}_0} \le 3{\epsilon }\ , \quad \Vert z(0) \Vert _s \le {\epsilon }^\theta \ , \quad \Vert z(T_1) \Vert _s \ge {\epsilon }^{-\theta } \ . \end{aligned}$$

#### Proof

Define ${\epsilon }_2:= \min ({\epsilon }_\star , {\epsilon }_0, {\epsilon }_1,{\mathfrak {r}})$ with ${\epsilon }_\star$ of Lemma 5.3, ${\epsilon }_0$ of Lemma 6.7 and ${\epsilon }_1$ of Proposition 6.5. First note that the solution *z*(*t*) is long-time controlled with parameters $\big (s, \theta , T_1, {\epsilon }\big )$ (see Definition 5.2); indeed condition (A1) holds true in view of the $L^2$-smallness of Lemma 6.7, whereas condition (A2) holds true with $T_\star \leadsto T_1$ by the minimality of $T_1$.

We now show that $T_1$ is finite and bounded by $\frac{T_0}{{\epsilon }^2}\log \left( \frac{1}{{\epsilon }} \right)$. Assume by contradiction that $T_1 > {T_0}{{\epsilon }^{-2}}\log \left( {\epsilon }^{-1} \right)$. Then, by the very definition of $T_1$,

$$\begin{aligned} \sup _{0 \le t \le {T_0}{{\epsilon }^{-2}}\log \left( {\epsilon }^{-1} \right) } \Vert z(t) \Vert _s \le {\epsilon }^{-\theta } ; \end{aligned}$$

namely, the solution *z*(*t*) is long-time controlled also with parameters $\big (s, \theta , \frac{T_0}{{\epsilon }^2}\log \left( \frac{1}{{\epsilon }} \right) , {\epsilon }\big )$. Then, since by Lemma 6.7 the initial data *z*(0) is well prepared, Proposition 6.5 applies, and therefore

$$\begin{aligned} \sup _{0 \le t \le {T_0}{{\epsilon }^{-2}}\log \left( {\epsilon }^{-1} \right) } \Vert z(t) \Vert _s \ge {\epsilon }^{-\theta } \ , \end{aligned}$$

contradicting the minimality of $T_1$. This proves that $T_1 \le \frac{T_0}{{\epsilon }^2}\log \left( \frac{1}{{\epsilon }} \right)$.

To control the low norm $\Vert z(t) \Vert _{{\mathfrak {s}}_0}$, we apply the bootstrap lemma 5.3 with the parameter $T_\star =T_1$ that we have just proved satisfy the required condition (5.16). The last two inequalities of (6.47) follow by (6.42) and (6.46). $\square$

We conclude with

#### Proof of Theorem 1.1

Recall that the variables *u*(*t*) and *z*(*t*) are related by the admissible transformation $Z(t) ={{\mathcal {F}}}(U(t))\equiv \textbf{F}(U(t))U(t)$ in (4.37). By Lemma 2.14, the map $Z={{\mathcal {F}}}(U)$ is locally invertible provided $\Vert Z \Vert _{{\mathfrak {s}}_0}\le r'$ is sufficiently small, and has the form ${{\mathcal {F}}}^{-1}(Z)= \textbf{G}(Z)Z$ for some $\textbf{G}(Z)$ fulfilling the bound in (2.43).

We consider that $$Z(0) = \Big ({\begin{smallmatrix}z(0) \\ {\overline{z}}(0)\end{smallmatrix}}\Big )$$ with *z*(0) (as in Lemma 6.7) fulfills $\Vert Z(0) \Vert _{{\mathfrak {s}}_0} \le {\epsilon }^\theta \le {\mathfrak {r}}$. We define that

$$\begin{aligned} U(0) := {{\mathcal {F}}}^{-1}(Z(0))=\textbf{G}(Z(0))Z(0) \ . \end{aligned}$$

We take *U*(0) as the initial data for equation (1.1); by (2.55), its Sobolev norm is

$$\begin{aligned} \Vert U(0) \Vert _s \le C_s \Vert Z(0) \Vert _s {\mathop {\le }\limits ^{(6.47)}} C_s {\epsilon }^\theta \ . \end{aligned}$$

Consider now the solution *U*(*t*) of (1.1) with initial data *U*(0). By Theorem 4.4, $Z(t) ={{\mathcal {F}}}(U(t))$ is the solution of equation (4.7) with initial datum *Z*(0) of Lemma 6.7; consequently, in view of Lemma 5.1 and Lemma 6.8, *z*(*t*) has a small $H^{{\mathfrak {s}}_0}$-norm for all times $0 \le t \le T_1$, but large $H^s$-norm at time $T_1$. We deduce that $U(t) = {{\mathcal {F}}}^{-1}(Z(t))$ fulfills the bound

$$\begin{aligned} \Vert U(t) \Vert _{{\mathfrak {s}}_0} \le C_{{\mathfrak {s}}_0} \Vert Z(t) \Vert _{{\mathfrak {s}}_0} \le C_{{\mathfrak {s}}_0} {\epsilon }< {\mathfrak {r}}, \quad \forall 0 \le t \le T_1 \ . \end{aligned}$$

At time $T_1$, we bound from below the $H^s$-norm of $U(T_1)$ using the identity $Z(T_1) = {{\mathcal {F}}}(U(T_1))$, the fact that $\Vert U(T_1) \Vert _{{\mathfrak {s}}_0} \le {\mathfrak {r}}$ and estimate (2.43), to get

$$\begin{aligned} \Vert U(T_1) \Vert _s \ge C_s^{-1} \Vert Z(T_1) \Vert _s {\mathop {\ge }\limits ^{(6.47)}} C_s^{-1} {\epsilon }^{-\theta } \ . \end{aligned}$$

Given arbitrary $\delta \in (0,1)$ and $K \ge 1$, we shrink ${\epsilon }$ to conclude the proof of Theorem 1.1. $\square$

## High Frequency Paradifferential Calculus

In this section we consider paradifferential operators with symbols supported only on high frequencies and prove a commutator estimate and a Garding inequality keeping track of the size of the support of the symbols.

### Lemma A.1

Let $N \in {{\mathbb {N}}}_0$, $m \in {{\mathbb {R}}}$ and ${\mathtt R}\ge 1$. If $a \in \Gamma ^m_{W^{N, \infty }}$, then

$$\begin{aligned} a_{\mathtt R}(x, \xi ):= a(x, \xi )\, \eta _{\mathtt R}(\xi ) , \quad \eta _{\mathtt R} \text{ in } (6.7)\end{aligned}$$

is a symbol in $\Gamma ^{m+\nu }_{W^{N, \infty }}$ for any $\nu \ge 0$ with quantitative bound

A.1 $$\begin{aligned} | a_{\mathtt R}|_{m + \nu , W^{N, \infty }, n} \le C_{n}\, {\mathtt R}^{-\nu } \, | a |_{m , W^{N, \infty }, n} \quad \text {for any } n\in {{\mathbb {N}}}_0 . \end{aligned}$$

In addition, if $N \ge 2$ and $b \in \Gamma ^{m'}_{W^{2, \infty }}$, $m' \in {{\mathbb {R}}}$, one has the commutator estimate

A.2 $$\begin{aligned} \Vert [ \textrm{Op}^{\!\scriptscriptstyle BW}\!\left( a_{\mathtt R}\right) , \textrm{Op}^{\!\scriptscriptstyle BW}\!\left( b\right) ] u \Vert _{s - m - m' - \nu +1 } \le C {\mathtt R}^{- \nu } \, | a |_{m , W^{2, \infty }, 7} \, | b |_{m' , W^{2, \infty }, 7} \, \Vert u \Vert _s \ .\nonumber \\ \end{aligned}$$

### Proof

For any $\alpha , \beta \in {{\mathbb {N}}}_0$, $\alpha \le N$, $\beta \le n$, we have

$$\begin{aligned} \left| ( \partial _x^\alpha \partial _\xi ^\beta a_{\mathtt R}(x, \xi ) \right|&\lesssim \sum _{\beta _1 + \beta _2 = \beta } \left| \partial _x^\alpha \partial _\xi ^{\beta _1} a(x, \xi ) \right| \, \left| \partial _\xi ^{\beta _2} \eta _{\mathtt R}(\xi ) \right| \\&\lesssim \sum _{\beta _1 + \beta _2 = \beta } |a|_{m, W^{N,\infty }, n}\langle \xi \rangle ^{m - \beta _1} \frac{1}{{\mathtt R}^{\beta _2}} \left| \eta ^{(\beta _2)}\big (\frac{\xi }{{\mathtt R}}\big ) \right| \\&\lesssim |a|_{m, W^{N,\infty }, n}\sum _{\beta _1 + \beta _2 = \beta } \langle \xi \rangle ^{m - \beta _1- \beta _2 + \nu } \sup _{\xi } \left| \langle \xi \rangle ^{-\nu } \langle \frac{\xi }{{\mathtt R}} \rangle ^{\beta _2} \eta ^{(\beta _2)}\big (\frac{\xi }{{\mathtt R}}\big ) \right| \\&\lesssim \frac{1}{{\mathtt R}^{\nu }} |a|_{m, W^{N,\infty }, n} \, \langle \xi \rangle ^{m - \beta + \nu }\ , \end{aligned}$$

where in the last step we used that the function $\langle \frac{\xi }{{\mathtt R}} \rangle ^{\beta _2} \eta ^{(\beta _2)}\big (\frac{\xi }{{\mathtt R}}\big )$ is uniformly bounded on ${{\mathbb {R}}}$ and has support on $\xi \ge {\mathtt R}$.

We prove now (A.2). By Proposition 2.8 with $\varrho =2$ we have

$$\begin{aligned} [ \textrm{Op}^{\!\scriptscriptstyle BW}\!\left( a_{\mathtt R}\right) , \textrm{Op}^{\!\scriptscriptstyle BW}\!\left( b\right) ] = \textrm{Op}^{\!\scriptscriptstyle BW}\!\left( \{a_{\mathtt R}, b \} \right) + R^{-2}(a_{\mathtt R}, b). \end{aligned}$$

We now bound both terms in the above equation regarding $a_{\mathtt R}$ as a symbol in $\Gamma ^{m+\nu }_{W^{N,\infty }}$ and $\{a_{\mathtt R}, b \}$ as a symbol in $\Gamma ^{m + m'+\nu -1}_{W^{N-1,\infty }}$. By (2.28) and (2.40), we get

A.3 $$\begin{aligned} \Vert \textrm{Op}^{\!\scriptscriptstyle BW}\!\left( \{a_{\mathtt R}, b \} \right) u \Vert _{s- m - m' -\nu +1}&\lesssim \left| \{a_{\mathtt R}, b \} \right| _{m+m'+\nu -1, L^\infty , 4} \ \Vert u \Vert _s \nonumber \\&\lesssim | a_{\mathtt R}|_{m+ \nu , W^{1,\infty }, 5} | b |_{ m', W^{1,\infty }, 5} \, \Vert u \Vert _s \nonumber \\&{\mathop {\lesssim }\limits ^{(A.1)}} {\mathtt R}^{- \nu } \, | a |_{m , W^{1,\infty }, 5} \, | b |_{m' , W^{1,\infty }, 5} \, \Vert u \Vert _s \ . \end{aligned}$$

Next we estimate the norm of $R^{-2}(a_{\mathtt R}, b)$ using (2.41):

A.4 $$\begin{aligned} \Vert R^{-2}(a_{\mathtt R}, b)u \Vert _{s-m - m' -\nu +2}&\lesssim | a_{\mathtt R}|_{m + \nu , W^{2,\infty }, 7} \, | b|_{m', W^{2,\infty }, 7} \, \Vert u \Vert _s \nonumber \\&{\mathop {\lesssim }\limits ^{(A.1)}} {\mathtt R}^{- \nu } \, | a |_{m , W^{2,\infty }, 7} \, | b |_{m' , W^{2,\infty }, 7} \, \Vert u \Vert _s \end{aligned}$$

In conclusion (A.2) follows from (A.3), (A.4). $\square$

In the following we shall use a well-known cancellation which is a direct consequence of Proposition 2.8: if $a\in \Gamma ^{m}_{W^{2,\infty }}$, $b\in \Gamma ^{m'}_{W^{2,\infty }}$, with $m, m' \in {{\mathbb {R}}}$, then

A.5 $$\begin{aligned} \textrm{Op}^{\!\scriptscriptstyle BW}\!\left( b\right) \circ \textrm{Op}^{\!\scriptscriptstyle BW}\!\left( a\right) \circ \textrm{Op}^{\!\scriptscriptstyle BW}\!\left( b\right) = \textrm{Op}^{\!\scriptscriptstyle BW}\!\left( ab^2\right) + R^{-2}(a,b), \end{aligned}$$

where $R^{-2}(a,b)$ is a bounded operator $H^s \rightarrow H^{s-(m+2m')+2}$, $\, \forall s\in {{\mathbb {R}}}$, satisfying, for any $u \in H^s$,

A.6 $$\begin{aligned} \Vert R^{-2}(a,b)\Vert _{s-(m+m') + 2} \lesssim |a|_{m,W^{2,\infty },8} \ |b|_{m',W^{2,\infty },8}^2 \ \Vert u \Vert _s. \end{aligned}$$

In the next lemma we prove a simplified version of the strong Garding inequality adapted to our setting.

### Lemma A.2

(Strong Garding’s inequality) Let ${\mathtt R}\ge 1$, $a(x) \in W^{3,\infty }$ and $a(x) \ge 0$. Let $\psi (\xi ) \in {\widetilde{\Gamma }}^{m}_0$, $m > 0$, a real valued Fourier multiplier with $supp \psi \subseteq [{\mathtt R}, +\infty )$. Then there is $C >0$ such that

A.7 $$\begin{aligned} \langle \textrm{Op}^{\!\scriptscriptstyle BW}\!\left( a(x) \psi ^2(\xi )\right) u , u \rangle \ge - C \frac{\Vert a \Vert _{W^{3,\infty }}}{{\mathtt R}^2} \Vert u \Vert _{m}^2 \ . \end{aligned}$$

### Proof

Arguing as in Lemma A.1 one shows that, for any $n \in {{\mathbb {N}}}_0$,

A.8 $$\begin{aligned} |\psi |_{m+1, L^\infty , n} \le C_n \frac{1}{{\mathtt R}} |\psi |_{m, L^\infty , n} \ . \end{aligned}$$

We apply now the composition formula (A.5) regarding $\psi (\xi )$ as a symbol in ${\widetilde{\Gamma }}^{m+1}_0$:

A.9 $$\begin{aligned} \textrm{Op}^{\!\scriptscriptstyle BW}\!\left( \psi \right) \circ \textrm{Op}^{\!\scriptscriptstyle BW}\!\left( a\right) \circ \textrm{Op}^{\!\scriptscriptstyle BW}\!\left( \psi \right) = \textrm{Op}^{\!\scriptscriptstyle BW}\!\left( a \psi ^2\right) + \textsf{R}_1 \end{aligned}$$

with $\textsf{R}_1:H^{m} \rightarrow H^{-m}$ fulfilling, by (A.6),

A.10 $$\begin{aligned} \Vert \textsf{R}_1 u \Vert _{-m} {\lesssim } \Vert a \Vert _{W^{2, \infty }} \, |\psi |_{m+1, L^\infty , 8}^2 \Vert u \Vert _m {\mathop {\lesssim }\limits ^{(A.8)}} \frac{1}{{\mathtt R}^2 }\Vert a \Vert _{W^{2,\infty }}\Vert u \Vert _m \ . \end{aligned}$$

Then observe that $\textrm{Op}^{\!\scriptscriptstyle BW}\!\left( \psi \right) = \textrm{Op}^{\scriptscriptstyle W}\!\left( \psi \right) = \psi (D)$ and $\textrm{Op}^{\!\scriptscriptstyle BW}\!\left( a\right) = \textrm{Op}^{\scriptscriptstyle W}\!\left( a\right) + \textrm{Op}^{\scriptscriptstyle W}\!\left( a_\chi - a\right)$, where $a_\chi$ is the cut-offed symbol defined in (2.21), so

A.11 $$\begin{aligned} \textrm{Op}^{\!\scriptscriptstyle BW}\!\left( \psi \right) \circ \textrm{Op}^{\!\scriptscriptstyle BW}\!\left( a\right) \circ \textrm{Op}^{\!\scriptscriptstyle BW}\!\left( \psi \right) = \psi (D) \circ \textrm{Op}^{\scriptscriptstyle W}\!\left( a\right) \circ \psi (D) + \textsf{R}_2 \end{aligned}$$

where $\textsf{R}_2:= \psi (D) \circ \textrm{Op}^{\scriptscriptstyle W}\!\left( a_\chi -a\right) \circ \psi (D) .$ Now we prove that $\textsf{R}_2$ is bounded $H^{m}\rightarrow H^{-m}$. First note that, by the definitions (2.21) and (2.23), for any $v\in H^{-1}$,

$$\begin{aligned} \Vert \textrm{Op}^{\scriptscriptstyle W}\!\left( a_\chi -a\right) v \Vert _{1}^2&\lesssim \sum _{j} \langle j \rangle ^2 \left| \sum _k {\widehat{a}}_{j-k} \, \Big (1- \chi \big (k-j, \frac{j+k}{2} \big ) \Big ) \, v_k \right| ^2 \\&\lesssim \sum _{j} \left| \sum _k \langle j-k \rangle ^2 \, |{\widehat{a}}_{j-k}| \, \Big (1- \chi \big (k-j, \frac{j+k}{2} \big ) \Big )\, \frac{1}{\langle k \rangle }| v_k| \right| ^2\\&\lesssim \sum _{j} \left| \sum _k \langle j-k \rangle ^2 | {\widehat{a}}_{j-k} | \, \frac{1}{\langle k \rangle }| v_k| \right| ^2\\&\lesssim \Vert a \Vert _{3}^2 \Vert v \Vert _{-1}^2 \lesssim \Vert a \Vert _{W^{3,\infty }}^2 \, \Vert v \Vert _{-1}^2 \end{aligned}$$

where to pass from the first to the second line we used that, on the support of $1- \chi \big (k-j, \frac{j+k}{2} \big )$, one has

$$\begin{aligned}\langle k \rangle , \langle j \rangle \lesssim \langle j - k \rangle + \langle j+k \rangle \lesssim \langle j - k \rangle \ , \end{aligned}$$

and to pass from the third to the last line we used Young’s inequality for convolution of sequences.

Thus we get, for any $u \in H^m$,

A.12 $$\begin{aligned} \Vert \textsf{R}_2 u \Vert _{-m}&\lesssim |\psi |_{m+1,L^\infty ,0} \, \Vert \textrm{Op}^{\scriptscriptstyle W}\!\left( a_\chi -a\right) \circ \psi (D) u \Vert _{1}\nonumber \\&\lesssim |\psi |_{m+1,L^\infty ,0} \, \Vert a \Vert _{W^{3,\infty }} \, \Vert \psi (D) u \Vert _{-1} \nonumber \\&\lesssim |\psi |_{m+1,L^\infty ,0}^2 \, \Vert a \Vert _{W^{3,\infty }} \, \Vert u \Vert _{m} {\mathop {\lesssim }\limits ^{(A.8)}} \frac{1}{{\mathtt R}^2} \Vert a \Vert _{W^{3,\infty }} \Vert u \Vert _m \ . \end{aligned}$$

In conclusion, combining (A.9) and (A.11) and since $\textrm{Op}^{\scriptscriptstyle W}\!\left( a\right) = a \ge 0$ and $\psi (D)$ is self-adjoint, we have that

$$\begin{aligned} 0 \le \langle \psi (D) \circ a \circ \psi (D) u, u \rangle = \langle \textrm{Op}^{\!\scriptscriptstyle BW}\!\left( a \psi ^2\right) u, u \rangle + \langle (\textsf{R}_1 - \textsf{R}_2) u, u \rangle , \end{aligned}$$

and (A.7) follows by (A.10) and (A.12). $\square$

## Flows and Conjugations

In this section we collect some results about the conjugation of paradifferential operators and smoothing remainders under flows, following [9, 11, 13, 63].

**Conjugation by a flow generated by a real symbol of order one.** Given a function $\beta \in {\widetilde{\mathcal {F}}}_2^{{\mathbb {R}}}$ gauge invariant, i.e. $\beta ({\mathtt g}_\theta U;\cdot ) = \beta (U;\cdot )$ for any $\theta \in {{\mathbb {T}}}$, consider the flow $\Phi ^\tau (u)$, $\tau \in [-1,1]$ defined by (4.24). It is standard (see e.g. Lemma 3.22 in [9]) that, for any $U \in B_{s_0,{{\mathbb {R}}}}(r)$ with $s_0>0$ sufficiently large and $r>0$ sufficiently small, the operator $\Phi ^\tau (U)\in {{\mathcal {L}}}( H^s({{\mathbb {T}}}, {{\mathbb {C}}}^2))$ for any $s \in {{\mathbb {R}}}$ with the quantitative estimate: there is a constant $C(s)>0$ such that for any $W\in H^{s}({{\mathbb {T}}}, {{\mathbb {C}}}^2)$, $\Vert \Phi ^\tau (U) W \Vert _{s}+\Vert \Phi ^\tau (U)^{-1} W \Vert _{s} \le C ( s ) \Vert W \Vert _{s}.$ Following [9], we define the path of diffeomorphism of ${{\mathbb {T}}}$ via

B.1 $$\begin{aligned}  &   \Psi ( U, \tau ;x ) :=x + \tau \beta ( U; x) \quad \text {with inverse} \quad \Psi ^{-1}( U, \tau ;y ) :=y + \breve{\beta }( U, \tau ;y) , \nonumber \\    &   \quad \ \breve{\beta }\in {{\mathcal {F}}}_{\ge 2}^{{\mathbb {R}}}[ r], \end{aligned}$$

and set that $\Psi (U;x):=\Psi (U, 1;x)$.

### Proposition B.1

(Conjugations for a transport flow) Let $m\in {{\mathbb {R}}}$, $\varrho >0$, and let $\Phi (U)$ be the flow generated by (4.24).

1. **Space conjugation of a para-differential operator:** Let $a \in \Sigma \Gamma ^{m}_2[ r]$ be a real symbol and $a^{( m)}( U;x, \xi ) :=\left. a( U; y, \xi \ \partial _y\Psi ^{-1}( U;y))\right| _{y=\Psi ( U;x)} \in \Sigma \Gamma ^{m}_2[ r]$. Then
  B.2 $$\begin{aligned} \begin{aligned} \Phi (U) \circ \textrm{Op}^{\scriptscriptstyle BW}_{\texttt {vec}}\!\left( a( U;x,\xi )\right) \circ \Phi (U)^{- 1} =&\ \textrm{Op}^{\scriptscriptstyle BW}_{\texttt {vec}}\!\left( a^{( m)}( U;x, \xi ) + a^{( m-2)}_{\ge 4 } ( U;x, \xi ) \right) + R_{\ge 4}(U) \\ =&\ \textrm{Op}^{\scriptscriptstyle BW}_{\texttt {vec}}\!\left( a( U;x,\xi ) + a^{( m)}_{\ge 4 }( U;x, \xi )\right) + R_{\ge 4}(U), \end{aligned}\nonumber \\ \end{aligned}$$
  where $a^{( m-2)}_{\ge 4 }( U;x,\xi )$ and $a^{( m)}_{\ge 4}( U;x,\xi )$ are non-homogeneous real symbols in $\Gamma ^{m-2}_{\ge 4}[ r]$respectively $\Gamma ^m_{\ge 4}[ r]$, whereas $R_{\ge 4}(U)$ is a real-to-real matrix of smoothing operators in ${{\mathcal {R}}}^{-\varrho +m }_{\ge 4}[ r]$. In addition if $a( U;x,\xi ) = V( U;x) \xi$ for some $V\in {\widetilde{{{\mathcal {F}}}}}^{{\mathbb {R}}}_2[ r]$ then in (B.2) $a^{( m-2 )}_{\ge 4 }\equiv 0$ and $a^{( m )}_{\ge 4} ( U;x, \xi ) = {V}'_{\ge 4} ( U;x) \xi$ for a suitable function ${V}'_{\ge 4}\in {{\mathcal {F}}}_{\ge 4}^{{\mathbb {R}}}[ r]$.
2. **Space conjugation of a Fourier multiplier** Let $\omega (\xi ) \in {\widetilde{\Gamma }}^\alpha _0$ be a real Fourier multiplier. Then
  B.3 $$\begin{aligned} \begin{aligned} \Phi (U) \circ \textrm{Op}^{\scriptscriptstyle BW}_{\texttt {vec}}\!\left( \textrm{i}\omega \right) \circ \Phi (U)^{- 1} =&\ \textrm{Op}^{\scriptscriptstyle BW}_{\texttt {vec}}\!\left( \textrm{i}\big ( \omega + a^{( \alpha )}_{2 }( U;x, \xi ) + a^{( \alpha )}_{\ge 4 }( U;x, \xi ) + a_{\ge 4}^{(\alpha -2)}(U; x, \xi )\big )\right) \\&+ R_2(U) + R_{\ge 4}(U), \end{aligned}\nonumber \\ \end{aligned}$$
  where
  $\bullet$ $a^{( \alpha )}_{2 }( U;x, \xi )$ is a real, zero-average, gauge invariant symbol in ${\widetilde{\Gamma }}^\alpha _2$;
  $\bullet$ $a^{( \alpha )}_{\ge 4 }( U;x, \xi )$ is a real non-homogeneous symbol in $\Gamma ^\alpha _{\ge 4}[r]$ and $a_{\ge 4}^{(\alpha -2)}(U; x, \xi )$ is a non-homogeneous symbol in $\Gamma ^{\alpha -2}_{\ge 4}[r]$;
  $\bullet$ $R_2(U)$ is a real-to-real, gauge invariant matrix of smoothing operators in ${\widetilde{{{\mathcal {R}}}}}^{-\varrho +m}_2$, and $R_{\ge 4}(U)$ is a real-to-real matrix of non-homogeneous smoothing operators in ${{\mathcal {R}}}^{-\varrho +m}_{\ge 4}$.
3. **Space conjugation of a smoothing remainder:** If $$ R_2(U)$$ is a real-to-real matrix of smoothing operators in $$ {\widetilde{{{\mathcal {R}}}}}^{-\varrho }_2[ r] $$ then
  $$\begin{aligned} \Phi ( U) \circ R_2 ( U) \circ \Phi ( U)^{-1} = R_2(U)+ R_{\ge 4 }( U), \end{aligned}$$
  where $R_{\ge 4}(U)$ is a real-to-real matrix of smoothing operators in ${{\mathcal {R}}}^{-\varrho +1}_{\ge 4}[ r]$.
4. **Conjugation of** $\partial _t$: If *U* is a solution of (4.1) then
  B.4 $$\begin{aligned} (\partial _t \Phi ( U))\, \Phi ( U)^{-1} = \textrm{i}\ \textrm{Op}^{\scriptscriptstyle BW}_{\texttt {vec}}\!\left( 2 \beta ( -\textrm{i}{\mathbf {\Omega }}(D)U,U; x )\ \xi + \textrm{i}\ \textsf{V}_{\ge 4}( U;x ) \xi \right) + R_{\ge 4} ( U),\nonumber \\ \end{aligned}$$
  where ${\mathbf {\Omega }}(D)$ is the matrix of real Fourier multipliers in (3.17), $\textsf{V}_{\ge 4}( U;x)$ is a real function in ${{\mathcal {F}}}_{\ge 4}^{{\mathbb {R}}}[ r]$ and $R_{\ge 4}( U)$ is a real-to-real matrix of smoothing operators in ${{\mathcal {R}}}^{-\varrho }_{\ge 4}[ r]$.

### Proof

During the proof, we shall denote that $b:= \frac{\beta }{1+\tau \beta _x}$.

1. Follows by Lemmas A4 and A5 in [11].

2. We first define the operator $P^\tau (U):= \Phi ^\tau ( U) \circ \textrm{Op}^{\scriptscriptstyle BW}_{\texttt {vec}}\!\left( \textrm{i}\omega \right) \circ \big (\Phi ^\tau ( U)\big )^{-1}.$ Note that $P^\tau (U)$ is gauge invariant being composition of gauge invariant operators. By Theorem 3.27 in [9] (actually adapting that result when the function $\beta$ is 2-homogeneous rather than 1-homogeneous), we have, for any $\tau \in [0,1]$,

B.5 $$\begin{aligned} \begin{aligned} P^\tau (U) =&\textrm{Op}^{\scriptscriptstyle BW}_{\texttt {vec}}\!\left( \textrm{i}\omega _\Phi ^{(\alpha )} + \textrm{i}\omega ^{(\alpha -2)}\right) + R(U,\tau ) \\ =&\textrm{Op}^{\scriptscriptstyle BW}_{\texttt {vec}}\!\left( \textrm{i}\omega + \textrm{i}\omega _2^{(\alpha )}+ \textrm{i}a_{\ge 4}^{(\alpha )}+\textrm{i}\omega _2^{(\alpha -2)}+\textrm{i}a_{\ge 4}^{(\alpha -2)}\right) + R_2(U,\tau )+ R_{\ge 4}(U,\tau ) \end{aligned},\nonumber \\ \end{aligned}$$

where $\omega _\Phi ^{(m)} = \omega + \omega _2^{(\alpha )}+ a_{\ge 4}^{(\alpha )}$ is a real symbol in $\Sigma \Gamma ^\alpha _0[r]$, $\omega ^{(\alpha -2)}=\omega _2^{(\alpha -2)}+ a_{\ge 4}^{(\alpha -2)}$ is a symbol in $\Sigma \Gamma ^{\alpha -2}_2[r]$ and $R = R_2+ R_{\ge 4} \in \Sigma {{\mathcal {R}}}^{-\varrho +\alpha }_2[r]$.

To identify the quadratic component of $P^1(U)$ we use the Taylor expansion $P^1(U) = P^0(U) + \partial _\tau P^\tau (U)\vert _{\tau =0} +\int _0^1(1-\tau ) \partial _\tau ^2 P^\tau (U) \textrm{d}\tau$ and exploit that $P^\tau (U)$ fulfills the Heisenberg equation $\partial _\tau P^\tau (U)= [G(U,\tau ),{P^\tau (U)}] , \quad P^0(U) = \textrm{Op}^{\scriptscriptstyle BW}_{\texttt {vec}}\!\left( \textrm{i}\omega \right)$ . Using that $G(U,0) = \textrm{Op}^{\scriptscriptstyle BW}_{\texttt {vec}}\!\left( \textrm{i}b(U)\xi \right)$ and the paradifferential structure of $P^\tau (U)$ in (B.5), we obtain

$$\begin{aligned} P^1(U) = \textrm{Op}^{\scriptscriptstyle BW}_{\texttt {vec}}\!\left( \textrm{i}\omega \right) + [\textrm{Op}^{\scriptscriptstyle BW}_{\texttt {vec}}\!\left( \textrm{i}b(U)\xi \right) , \textrm{Op}^{\scriptscriptstyle BW}_{\texttt {vec}}\!\left( \textrm{i}\omega \right) ] + M_{\ge 4}(U), \end{aligned}$$

with $M_{\ge 4}(U)$ a $\alpha$-operator in ${{\mathcal {M}}}_{\ge 4}^{\alpha }[r]$. Now we use the composition Theorem 2.8 (with $\varrho \leadsto \varrho +1$) and formula (2.38) to expand the commutator as

B.6 $$\begin{aligned} P^1(U) = \textrm{Op}^{\scriptscriptstyle BW}_{\texttt {vec}}\!\left( \textrm{i}\omega + \textrm{i}a_2^{(\alpha )}\right) + R_2(U) + M_{\ge 4}(U), \end{aligned}$$

with $a_2^{(\alpha )}(U;x, \xi )$ the real, zero-average symbol

B.7 $$\begin{aligned} a^{( m)}_{2 }( U;x, \xi ) := \sum _{k=1}^{\varrho +1} \frac{(-1)^k -1}{2^k k!} (D_x^k \beta )\, (\partial _\xi ^k \omega )\, \textrm{i}\xi \in {\widetilde{\Gamma }}^\alpha _2 \ , \end{aligned}$$

and $R_2(U) \in {\widetilde{{{\mathcal {R}}}}}^{-\varrho +\alpha }_2$. Identifying the quadratic components of $P^1(U)$ in (B.5) and (B.6) we get that

$$\begin{aligned} \textrm{Op}^{\!\scriptscriptstyle BW}\!\left( \omega _2^{(\alpha )} + \omega _2^{(\alpha -2)}\right) = \textrm{Op}^{\!\scriptscriptstyle BW}\!\left( a_2^{(\alpha )}\right) + {\tilde{R}}_2(U) \end{aligned}$$

and therefore we get the thesis. Since $\beta (U)$ is gauge invariant (fulfills the first of (2.26)), so is $a_2^{(\alpha )}$ in (B.7). Finally, since $P^1(U)$ is gauge invariant, also $R_2(U)$ in (B.6) is gauge invariant by difference.

3. It follows as in [9, Remark at pag. 89] (see also [63, Proposition A.2] for details).

4. Differentiating

$$\begin{aligned} {\left\{ \begin{array}{ll} \partial _\tau \Phi ^\tau (U(t))= G(U(t))\Phi ^\tau (U(t))\\ \Phi ^0(U(t))=\textrm{Id}, \end{array}\right. }\end{aligned}$$

with respect to time, we get that $\partial _t \Phi ^\tau (U(t))$ fulfills the variational equation

B.8 $$\begin{aligned} {\left\{ \begin{array}{ll} \partial _\tau \, \left( \partial _t \Phi ^\tau (U(t))\right) =G(U(t)) \, \left( \partial _t \Phi ^\tau (U(t))\right) + \left( \partial _t G(U(t))\right) \, \Phi ^\tau (U(t)) \\ \partial _t \Phi ^0(U(t))=0 \end{array}\right. } \ , \end{aligned}$$

whose solution is given by the Duhamel formula

B.9 $$\begin{aligned} \left( \partial _t \Phi ^\tau (U(t))\right) =&\Phi ^\tau (U(t)) \int _0^\tau \Phi ^{\tau _1}(U(t))^{-1} \left( \partial _t G(U(t))\right) \Phi ^{\tau _1} (U(t))\, \textrm{d}\tau _1 \ . \end{aligned}$$

Evaluating at $\tau =1$, applying $\Phi ^1(U)^{-1}$ to the right and using that in our case $\partial _t G(U(t)) = \textrm{Op}^{\scriptscriptstyle BW}_{\texttt {vec}}\!\left( \partial _t b(U,\tau _1;x) \textrm{i}\xi \right)$ yields

B.10 $$\begin{aligned} \big (\partial _t \Phi ^1( U) \big ) \Phi ^1( U)^{-1}= \int _0^1 \Phi ^1 ( U) \left[ \Phi ^{\tau _1} ( U)\right] ^{-1} \textrm{Op}^{\scriptscriptstyle BW}_{\texttt {vec}}\!\left( \partial _t b(U,\tau _1;x) \textrm{i}\xi \right) \Phi ^{\tau _1} ( U)[\Phi ^1 ( U)]^{-1}\, \textrm{d}\tau _1.\nonumber \\ \end{aligned}$$

We claim that

B.11 $$\begin{aligned} \partial _t b(U,\tau ;x)= \beta (-\textrm{i}{\mathbf {\Omega }}(D)U;x)+ \textsf{V}_{\ge 4}(U,\tau ;x), \quad \textsf{V}_{\ge 4}\in \mathcal {F}_{\ge 4}^{{{\mathbb {R}}}}[r] \ . \end{aligned}$$

Differentiating $b(U(t),\tau ;x)$ with respect to *t* and using that, by equation (4.1), $\partial _t \beta (U) = 2 \beta (\partial _t U, U) = 2\beta (X(U), U)$ with $X(U) = - \textrm{i}{\mathbf {\Omega }}(D) U + X_3(U)$ we get

$$\begin{aligned}&\partial _t b(U,\tau ;x) = 2\beta ( -\textrm{i}{\mathbf {\Omega }}(D) U, U ; x)\\&+ \underbrace{ 2\beta ( M_{\texttt{NLS}}( U)U, U ; x) - 2\tau \left[ \frac{\beta ( U ; x)\beta _x( X( U),U; x)}{(1+\tau \beta _x( U; x ))^2} + \frac{\beta _x( U; x)\beta ( X( U), U; x)}{(1+\tau \beta _x( U; x ))}\right] }_{=:\textsf{V}_{\ge 4}(U,\tau ;x)} \ . \end{aligned}$$

Then (B.11) follows using Lemma 2.10–1 for each internal composition, getting that $\textsf{V}_{\ge 4}(U,\tau ;x)$ is a function in $\mathcal {F}_{\ge 4}^{{{\mathbb {R}}}}[r]$.

$$\square $$

**Conjugation by flows generated by linear smoothing operators.** In this section we study the conjugation rules for a flow $\Upsilon (U):=\Upsilon ^{\tau }(U)\vert _{\tau =1}$ generated by

B.12 $$\begin{aligned} \partial _\tau \Upsilon (U) = Q(U) \circ \Upsilon ^\tau (U), \ \ \Phi ^0_{Q}(U) = \textrm{Id}\,, \, \end{aligned}$$

with *Q*(*U*) a matrix of smoothing operators in ${\widetilde{{{\mathcal {R}}}}}^{-\varrho }_2$. We denote the inverse of $\Phi _{Q}(u)$ as $\Upsilon (U)^{- 1} = \left. \Upsilon ^\tau (U)\right| _{ \tau = - 1}$.

The following result is a small variation of [63, Proposition A.5] and we omit the proof.

### Proposition B.2

(Conjugation by flows generated by smoothing operators) Let $m\in {{\mathbb {R}}}$, $\varrho , \varrho ', r >0$. Let *Q*(*U*) be a matrix of smoothing operators in $\widetilde{\mathcal {R}}^{-\varrho }_2$ and $\Upsilon (U)$ be the flow generated by *Q*(*U*) as in (B.12). Then the following holds:

- *i*) **Space conjugation:** If $a\in \Sigma \Gamma ^m_2[r]$, then
  $$\begin{aligned} \Upsilon (U)\circ \textrm{Op}^{\scriptscriptstyle BW}_{\texttt {vec}}\!\left( a( U;x,\xi )\right) \circ \Upsilon (U)^{-1} - \textrm{Op}^{\scriptscriptstyle BW}_{\texttt {vec}}\!\left( a ( U;x,\xi )\right)&\in {{\mathcal {R}}}^{-\varrho + \max \{m,0\}}_{\ge 4 }[r], \\ \Upsilon (U)\circ (-\textrm{i}{\mathbf {\Omega }}(D) ) \circ \Upsilon (U)^{-1}- ( - \textrm{i}{\mathbf {\Omega }}(D)+ [Q(U), -\textrm{i}{\mathbf {\Omega }}(D)] )&\in {{\mathcal {R}}}^{-\varrho + \alpha }_{\ge 4 }[r] \ . \end{aligned}$$
  These matrices of operators are real-to-real, provided that *Q*(*U*) is.
- *ii*) **Conjugation of smoothing operators:** If *R*(*U*) is a real-to-real matrix of smoothing operators in $\Sigma {{\mathcal {R}}}^{-\varrho '}_2[r]$, then
  $$\begin{aligned} \Upsilon (U)\circ R( U) \circ \Upsilon (U)^{-1} - R( U) \in {{\mathcal {R}}}^{-\min \{\varrho ,\varrho '\}}_{\ge 4 }[r] \ \end{aligned}$$
  and it is real-to-real.
- *iii*) **Conjugation of** $\partial _t$: If *U* is a solution of (4.1), then
  $$\begin{aligned} (\partial _t \Upsilon ( U))\circ \Upsilon ( U)^{- 1} - 2 Q( -\textrm{i}{\mathbf {\Omega }}(D) U, U) \in {{\mathcal {R}}}^{-\varrho +1}_{\ge 4}[ r] , \end{aligned}$$
  and it is real-to-real.

## Footnotes

1 In particular, the ill-posedness phenomena à la Christ [21], which require non-hyperbolic nonlinearities like $u^{p-1}u_x$, do not happen for (1.1)
